# Total Synthesis and Biological Evaluation of Kakeromamide A and Its Analogues

**DOI:** 10.3389/fchem.2020.00410

**Published:** 2020-05-13

**Authors:** Meng Zhao, Yi Xiao, Satoshi Otsuka, Yoichi Nakao, Yian Guo, Tao Ye

**Affiliations:** ^1^State Key Laboratory of Chemical Oncogenomics, Peking University Shenzhen Graduate School, Shenzhen, China; ^2^Research Institute for Science and Engineering, Waseda University, Tokyo, Japan; ^3^Department of Chemistry and Biochemistry, Graduate School of Advanced Science and Engineering, Waseda University, Tokyo, Japan; ^4^Tsinghua Shenzhen International Graduate School, Xili, Shenzhen, China

**Keywords:** kakeromamide A, total synthesis, biological evaluations, marine cyclopeptide, structural analogs

## Abstract

Kakeromamide A (**1**), the first marine cyclopeptide inducing neural stem cells differentiation into astrocytes, was synthesized in 12 longest linear steps and 14% overall yield. Using this synthetic approach, four analogs of kakeromamide A were prepared and evaluated for neural differentiation- modulating activity.

## Introduction

**Figure 1:**
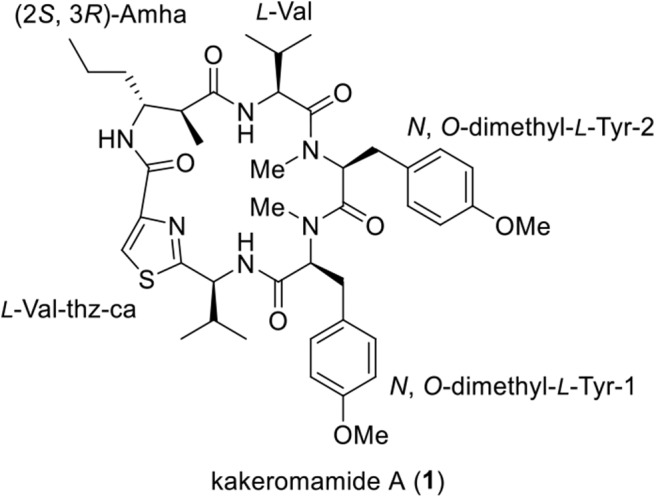
Structure of Kakeromamide A (**1**).

Marine cyanobacteria have been playing a pivotal role in producing structurally diversified and biologically intriguing peptides (Luesch et al., 2001; Guzman-Martinez et al., 2007; Zainuddin et al., 2007). In 2018, we reported the isolation of a novel cyclopeptide, kakeromamide A (Figure 1), from the marine cyanobacterium *Moorea bouillonii* collected near Japan (Nakamura et al., 2018). Preliminary biological tests revealed that kakeromamide A exhibits moderate cytotoxicity (IC_50_ = 10 μM) against Hela cells. To the best of our knowledge, this is the first example of marine cyclopeptide that can induce neural stem cells differentiation into astrocytes (2.5–10 μM). The constitutional information of kakeromamide A, featuring a peptidal macrocycle embedded with a thiazole ring and a β-amino acid, was revealed on the basis of in-depth spectroscopic analysis. The absolute configurations of kakeromamide A (**1**) were established by the advanced Marfey's method. Our laboratory's long-standing interest in the synthesis of bioactive marine natural products (Yang et al., 2000; Lei et al., 2014; Liao et al., 2016; Zhou et al., 2016; Guo et al., 2017; Cheng et al., 2018; Yu et al., 2019) prompted us to launch a synthetic program targeting kakeromamide A and its structural variants.

## Materials and Methods

Experimental procedure and compound characterization data are furnished in the Supplementary Material (Data Sheet 1).

## Results and Discussion

**Figure 2:**
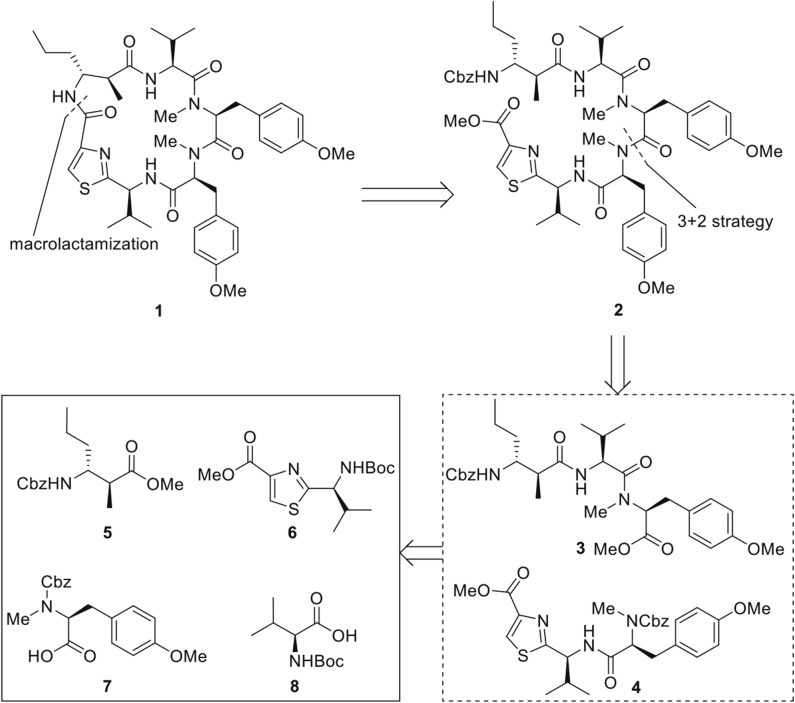
Retrosynthetic Plan of Kakeromamide A (**1**).

The retrosynthetic plan is depicted below (Figure 2). Strategic disconnection of the macrocycle at the least sterically demanding amide bond results in the linear pentapeptide **2**. Further dissection of **2** reveals that the pentapeptide may be most conveniently constructed by the assembly of tripeptide **3** and dipeptide **4**, derived from the disconnection of the bonds between two tyrosines. Tripeptide **3** and dipeptide **4** could be constructed from amino acid derivatives **5**–**8**. The 3+2 strategy has the advantage in terms of convergence and efficiency. However, the amidation between polypeptidyl acids and *N*-methyl amines is sometimes unattainable because of notoriously latent epimerization (Humphrey and Chamberlin, 1997; Teixidó et al., 2005; Nabika et al., 2014). Therefore, as an alternative plan, we envisioned that the pentapeptide **2** could also be assembled in a linear fashion with corresponding amino acid synthons **5**–**8**, which could circumvent the potential loss of stereo-integrity via oxazolone mechanism.

**Figure 3:**
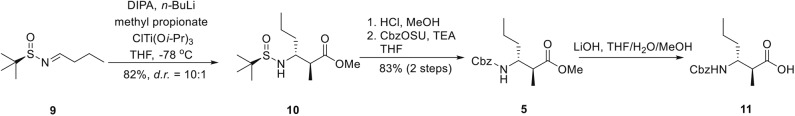
Synthesis of β-amino acid **11**.

As shown in Figure 3, the synthesis of the nonconventional β-amino acid (**11**) commenced with the known *N*-*tert*-butanesulfinimine **9** (Staas et al., 2002). Following Ellman's protocol (Tang and Ellman, 1999, 2002), methyl propionate was treated with lithium diisopropylamide (LDA) and the following transmetalation with chlorotriisopropoxytitanium provided the titanium enolate, which added to sulfinimine **9** in THF at −78°C to furnish the α,β-disubstituted β-amino ester **10** in 82% yield. The high (10:1) diastereoselectivity observed in the addition process may be explained by analysis of Zimmerman–Traxler transition state Zimmerman and Traxler, 1957 during the nucleophilic addition step. Acidic cleavage of the chiral auxiliary was achieved by exposure of **10** to methanolic HCl solution and the resulting amine was protected by Cbz group to afford **5** in 83% yield over two steps. Saponification of methyl ester **5** with lithium hydroxide underwent smoothly to afford the desired acid **11**, which was readily used in the following fragment coupling process without further purification.

**Figure 4:**
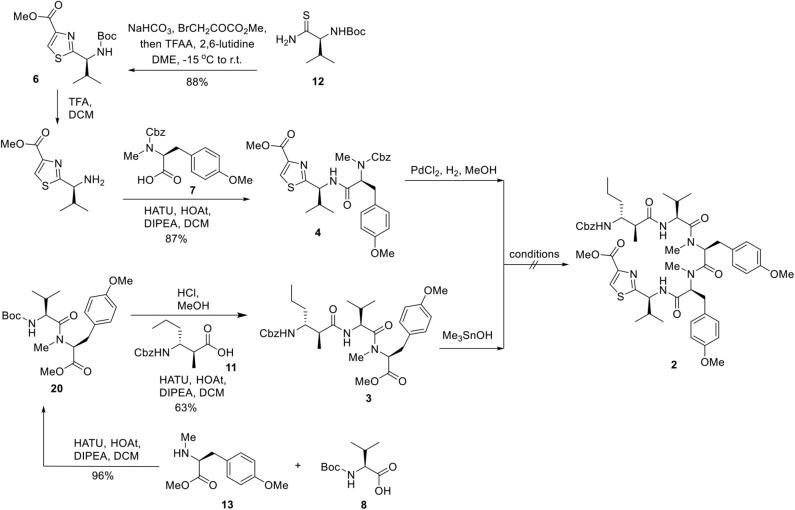
3+2 Strategy for the Synthesis of Pentapeptide **2**.

We next embarked on the assembly of the pentapeptide **2** (Figure 4). The synthesis began with the preparation of thiazole amino acid **6** from the known thioamide **12** (Aguilar and Meyers, 1994) following the modified Hantzsch conditions reported by Aguilar and Meyers (1994). Thus, thioamide **12** was treated with sodium bicarbonate and methyl bromopyruvate in dimethoxyethane and generated the intermediary hydroxythiazoline, which underwent dehydrative aromatization under the mediation of trifluoroacetic anhydride and 2,6-lutidine to afford thiazole amino acid **6** in 88% yield. Removal of the Boc group was accomplished by the treatment of thiazole amino acid **6** with trifluoroacetic acid to provide the corresponding ammonium salt, which was then subjected to 1-[bis(dimethylamino)methylene]-1*H*-1,2,3-triazolo[4,5-*b*]pyridinium 3-oxid hexafluorophosphate (HATU) promoted amidation conditions and successfully incorporated the known *L*-tyrosine derivative **7** (Ramanjulu et al., 1997) to afford the dipeptide **4** in 77% yield over three steps. In parallel, the known *L*-tyrosine derivative **13** (Chen et al., 2002) was converted into tripeptide **3** via a three-step sequence of transformations including HATU/HOAt promoted coupling of amino ester **13** with *N*-Boc-*L*-valine (**8**) to provide dipeptide **20** in 96% yield; Cleavage of the *N*-terminal Boc protective group of **20** (HCl, MeOH) to give rise to the corresponding amine followed by coupling to Cbz protected beta amino acid **11** to provided **3** in 63% yield. With both dipeptide **4** and tripeptide **3** in hand, the stage was now set for their assembly to afford the corresponding macrocyclization precursor **2**. In the event, methyl ester of **3** was saponified, in quantitative yield, to its corresponding carboxylic acid by the mild action of Me_3_SnOH. Reaction of this carboxylic acid with the amine derived from PdCl_2_ deprotection of Cbz-protected **4** under a variety of peptide coupling procedures including EDCI–HOAt, HATU, PyAOP, BOPCl, and Mukaiyama reagent, afforded at low yields of pentapeptide **2** as a diastereomeric mixtures. Apparently, somewhere along the line, most likely at the fragment coupling step, the conditions employed caused epimerization. This outcome, a consequence of the sensitivity of these molecules toward epimerization, forced upon us a change in strategy and tactics toward pentapeptide **2**, as will be described below.

**Figure 5:**
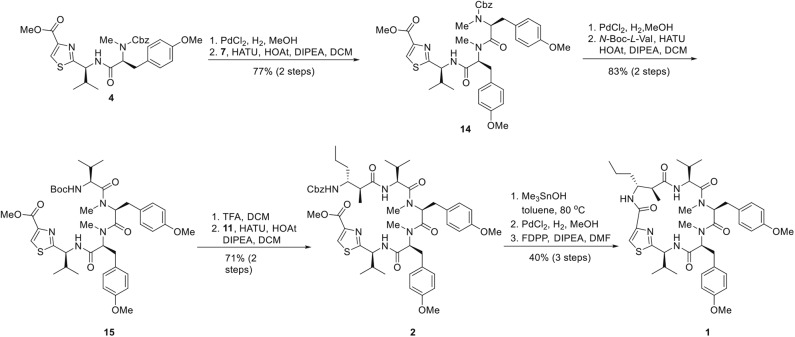
Total Synthesis of Kakeromamide A (**1**).

In order to circumvent the problems encountered in the 3+2 route (Figure 4), a more stepwise approach starting from dipeptide **4** was pursued. Thus, hydrogenolysis of the Cbz group in **4** using palladium chloride smoothly afforded the corresponding amine as its HCl salt, which was then condensed with the known *L*-tyrosine derivative **7** to provide tripeptide **14** in 77% yield over two steps. Removal of the Cbz group of **14** under identical conditions as for **4**, and the resulting amine salt was coupled with *N*-Boc-*L*-valine in the presence of HATU/HOAt and DIPEA tetrapeptide **15** in 83% yield. To continue the linear chain elongation, Boc group was selectively removed with TFA/DCM, further coupling with the β-amino acid **11** promoted by HATU/HOAt and DIPEA to give rise to the desired linear pentapeptide **2** in 71% yield. To set the stage for the final macrocyclization (Figure 5), the C-terminal methyl ester was saponified with trimethyltin hydroxide (Furlán et al., 1996; Nicolaou et al., 2005) in toluene at 80°C. Cleavage of the N-terminal Cbz protective group under identical conditions as for **4** provided the corresponding amino acid cyclization precursor, which was immediately subjected to macrolactamization as promoted by pentafluorophenyl diphenylphosphinate (FDPP) (Chen and Xu, 1991; Pradhan et al., 2013) and DIPEA in DMF to provide kakeromamide A (**1**) in 40% yield over three steps. The spectroscopic data of the synthetic material were consistent with those for the natural kakeromamide A, as evident from the ^1^H and ^13^C NMR spectra and optical rotation ${\left[\alpha \right]}_{D}^{22}$ = +6.15 (c 0.065, MeOH); versus the reported ${\left[\alpha \right]}_{D}^{23.8}$ = +6.2 (c 0.065, MeOH); (Tables 1, 2). The synthesis therefore confirmed the relative and absolute configuration of the natural product and also sets the stage for a study of the structure–activity relationship of kakeromamide A, with analogs containing “point mutations” at every site within the cyclic compounds.

**Table 1:** Comparison of the ^1^H NMR Data of Synthesized **1** with Literature Data.

**No**.	**^1^H NMR (δ_H_)**	
	**Kakeromamide A*^***a***^***	**Compound 1*^***b***^***
NH-28	8.60 d (9.3)	8.60 d (9.0)
NH-34	8.51 d (10.4)	8.51 d (10.3)
H-26	8.01 s	8.01 s
H-19/H-23	6.99 d (8.6)	6.99 d (8.6)
H-10/H-14	6.88 d (8.7)	6.88 d (8.6)
H-11/H-13	6.77 d (8.7)	6.77 d (8.7)
H-20/H-22	6.56 d (8.6)	6.55 d (8.7)
NH-2	6.53 d (7.3)	6.53 d (4.7)
H-7	5.53 dd (11.3, 4.6)	5.53 dd (11.3, 4.7)
H-28	5.33 dd (9.3, 5.5)	5.33 dd (9.0, 5.4)
H-16	5.25 dd (9.7, 5.2)	5.25 dd (9.7, 5.2)
H-2	4.3 dd (9.8, 7.3)	4.29 dd (9.8, 7.1)
H-34	4.09 dddd (12.0, 10.4, 3.4, 2.5)	4.09 m
*O*-CH_3_	3.73 s	3.73 s
*O*-CH_3_	3.48 s	3.48 s
*N*-CH_3_	3.05 s	3.04 s
H-18	2.97 dd (14.4, 5.2)	2.96 dd (14.4, 5.1)
*N*-CH_3_	2.86 s	2.85 s
H-9	2.7 dd (16.4, 11.3)	2.69 dd (16.3, 11.3)
H-17	2.61 dd (14.4, 9.7)	2.61 m
H-33	2.60 dq (7.0, 3.4)	
H-29	2.01 dqq (6.8, 6.8, 5.5)	2.01 m
H-3	1.8 dqq (9.8, 6.8, 6.7)	1.80 m
H-35	1.7 dddd (14.1, 9.2, 7.0, 2.5)	1.70 m
H-36	1.45 m	1.46 m
H-8	1.38 dd (16.4, 4.6)	1.37 dd (16.3, 4.6)
H-36	1.27 m	1.26 m
H-38	1.09 d (7.0)	1.09 d (7.0)
H-35	1.07 m	1.07 m
H-37	0.97 t (7.5)	0.96 t (7.3)
H-31	0.94 d (6.8)	0.95 d (6.8)
H-5	0.89 d (6.7)	0.89 d (6.6)
H-4	0.79 d (6.8)	0.79 d (6.9)
H-30	0.78 d (6.8)	0.78 d (6.8)

a *Data obtained from the isolation paper (Nakamura et al., 2018)*.

b *Data recorded in CD_3_CN*.

**Table 2:** Comparison of the ^13^C NMR Data of Synthesized **1** with Literature Data.

**No**.	**^13^C NMR (δ_C_)**	
	**Kakeromamide A*^***a***^***	**Compound 1*^***b***^***
C-1	176.8	177.0
C-6	174.0	173.8
C-32	173.3	173.3
C-27	170.0	170.2
C-15	169.9	170.0
C-24	161.4	161.6
C-26	159.5	159.7
C-12	159.2	159.4
C-25	150.4	150.5
C-19/ C-23	131.3	131.5
C-18	131.0	131.1
C-9	130.1	130.3
C-10/C-14	130.0	130.1
C-26	123.6	123.8
C-20/C-22	115.2	115.3
C-11/C-33	114.9	115.0
C-16	63.7	63.9
C-2	57.2	57.4
C-28	57.2	57.4
*O*-CH_3_	55.9	56.1
*O*-CH_3_	55.7	55.8
C-34	52.8	53.0
C-7	52.3	52.4
C-33	44.6	44.7
C-29	36.8	36.9
C-17	34.6	34.7
C-8	33.4	33.5
C-3	32.0	32.1
C-35	31.9	32.1
*N*-CH_3_	31.7	31.8
*N*-CH_3_	29.7	29.9
C-31	20.8	21.0
C-36	20.2	20.4
C-5	20.2	20.4
C-4	18.9	19.0
C-30	17.8	18.0
C-37	14.6	14.8
C-38	14.4	14.5

a *Data obtained from the isolation paper (Nakamura et al., 2018)*.

b *Data recorded in CD_3_CN*.

**Figure 6:**
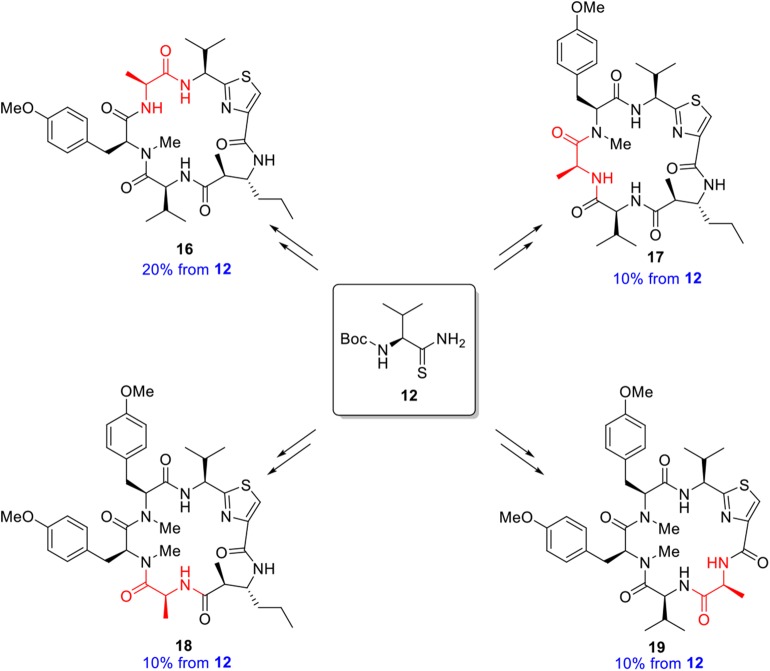
Synthesis of Structural Variants of Kakeromamide A.

With completion of the total synthesis of kakeromamide A, we decided to employ the same approach in our analog synthesis. An alanine scan (Morrison and Weiss, 2001; Shaheen et al., 2012) of each residue of the cyclopeptide may identify key sites responsible for or contributing to the biological properties. The preparation of a complete set of individual residue analogs should allow an assessment of each structural feature of kakeromamide A and provide a detailed account of the structure function relationships. In the event, four analogs **16**–**19** (Figure 6) of kakeromamide A, constituting the alanine scan, were constructed from thioamide **12** (Figure 5). Attempts to replace the thiazole amino acid with alanine failed to deliver the desired cyclopeptide from the corresponding linear precursor, presumably due to the added conformational constraints of the linear precursor during macrolactamization.

**Figure 7:**
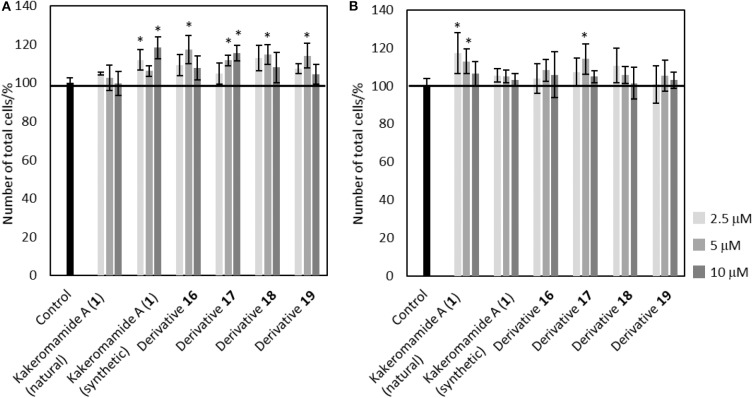
Cytotoxicity of Kakeromamide A and Its Analogs. **(A)** Percentages of cell numbers calculated by Hoechst 33342 positive cells, compared to that of control, in evaluating effects of kakeromamide A and its analogs for astrocytes (*n* = 3, means ± S.D., *p < 0.05). **(B)** Percentages of cell numbers calculated by Hoechst 33342 positive cells, compared to that of control, in evaluating effects of kakeromamide A and its analogs for neurons (*n* = 3, means ± S.D., *p < 0.05).

Bioactive compounds modulating proliferation and differentiation of neural stem cells are required as chemical probes to study about mechanism of neural development or lead compounds for regenerative medicine. In order to evaluate neural differentiation-modulating activities of kakeromamide A and its analogs, we elected to perform the biological tests using the same procedure that we reported in the isolation paper (Nakamura et al., 2018). Neural stem cells were obtained following the procedure (Nakayama and Inoue, 2006; Iwata et al., 2016) with modification. 2.5, 5, and 10 μM of kakeromamide A or its respective derivative was added to astrocytes or neurons. The cell cytotoxicity of kakeromamide A and its analogs toward astrocytes or neurons was carried out using Hoechst 33342 staining (Figure 7). No significant cytotoxicity was observed upon the treatment with kakeromamide A and its analogs.

**Figure 8:**
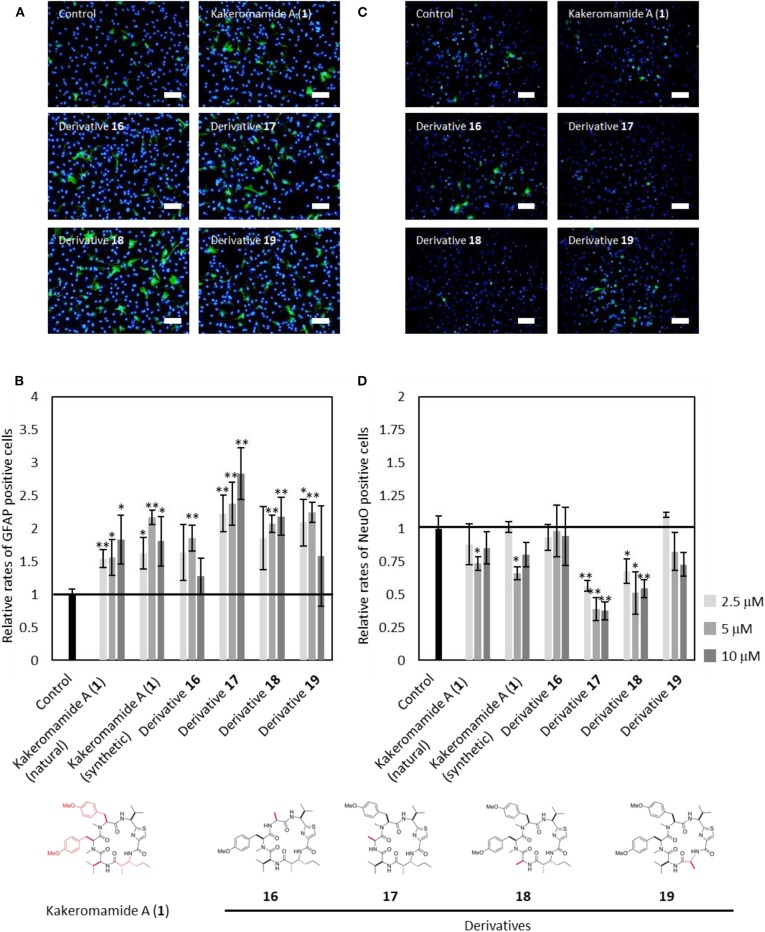
Neural Differentiation-Modulating Activity of Kakeromamide A and Its Analogs. **(A)** Fluorescent microscopic images of differentiation of neural stem cells (NSCs) into astrocyte. Astrocytes and nuclei were colored by anti-GFAP (glial fibrillary acidic protein, green) and Hoechst 33342 (blue), respectively (5 μM, Scale bar; 100 μm). **(B)** Rates for astrocytic differentiation of NSCs compared to that of control (*n* = 3, means ± S.D., *p < 0.05, **p < 0.01). **(C)** Fluorescent microscopic images of differentiation of NSCs into neuron. Neurons and nuclei were stained by NeuO (green) and Hoechst 33342 (blue), respectively (5 μM, Scale bar; 100 μm). **(D)** Rates for neuronal differentiation of NSCs compared to that of control (*n* = 3, means ± S.D., *p < 0.05, **p < 0.01).

Next, kakeromamide A or its respective derivative was exposed at three concentrations (2.5, 5, and 10 μM) during differentiation of neural stem cells (NSCs). The effects of kakeromamide A and its analogs on the neural differentiation into neurons or astrocytes were evaluated by using NeuO (Er et al., 2015) a fluorescent probe which could selectively stain neurons, or immunostaining of an astrocyte marker GFAP (glial fibrillary acidic protein), respectively. The nuclei were stained by using Hoechst 33342 followed by the microscopic analysis. Their activities were determined by calculating a ratio NeuO or GFAP positive cells to whole cells and comparing with that of the control. As a result, the synthetic product of kakeromamide A (**1**) showed the same activity as the sample isolated from natural source, promoting differentiation of NSCs into astrocytes while inhibiting neuronal differentiation of NSCs (Figure 8). Furthermore, the astrocytic or neuronal differentiation were promoted or inhibited, respectively, by treatment with four analogs of compound **1**. It was found that derivative **17** modulated the differentiation most strongly among derivatives. These results suggested that kakeromamide A and its analogs modulated neural differentiation of NSCs via common target (s) and especially derivative **17** had the highest affinity to the target (s).

## Conclusions

In summary, we have achieved the first synthesis of a new cyclopeptide kakeromamide A (**1**) from the known thioamide **12** in 14% overall yield with the longest linear sequence being 12 steps. The present work confirms the originally assigned structure of kakeromamide A (**1**) and also allows access to four analogs. Neural differentiation-modulating activities of kakeromamide A and four analogs were performed, which revealed that kakeromamide A and its analogs promoted astrocytic differentiation while inhibited neuronal differentiation of NSCs and derivative **17** showed the strongest activity. Further analyses of the detailed mechanism for their biological activity are currently under investigation in our laboratories and will be described in due course.

## Data Availability Statement

All datasets presented in this study are included in the article/Supplementary Material.

## Author Contributions

MZ: designed and conducted chemical synthesis experiments, analyzed data. YX: contributed to analyzed data. SO: designed and conducted biological activity experiments, analyzed data. YG: contributed to chemical data analysis and writing of the paper. YN: contributed to biological data analysis and writing of the paper. TY: designed experiments, analyzed data, and wrote the paper.

## Conflict of Interest

The authors declare that the research was conducted in the absence of any commercial or financial relationships that could be construed as a potential conflict of interest.
