# Supplementary material for: Total Synthesis and Biological Evaluation of Kakeromamide A and Its Analogues
Source: Front Chem. 2020 May 13;8:410. doi: 10.3389/fchem.2020.00410 (PMC7237762; doi:10.3389/fchem.2020.00410)

Supplementary Material

Total Synthesis and Biological Evaluation of Kakeromamide A and Its Analogues

Meng Zhao,[1] Yi Xiao,[1] Satoshi Otsuka,[3] Yoichi Nakao,[3,4] Yian Guo[1*], Tao Ye[1,2*]

State Key Laboratory of Chemical Oncogenomics, Peking University Shenzhen Graduate School, [1] Tsinghua Shenzhen International Graduate School, [2] Xili, Shenzhen, China, 518055.

Research Institute for Science and Engineering,[3] Department of Chemistry and Biochemistry, Graduate School of Advanced Science and Engineering,[4] Waseda University, 3-4-1 Okubo, Shinjuku-ku, Tokyo 169-8555, Japan

Table of Contents:

I. General Information S3

II. Spectroscopy, Spectrometry, and Data Collection S3

III. Experimental Details and Spectral Data for Total Synthesis of **Kakeromamide A (1)** S3

A. Procedure and Spectral Data for the Synthesis of Compound **6** S3

B. Procedure and Spectral Data for the Synthesis of Compound **10** S4

C. Procedure and Spectral Data for the Synthesis of Compound **5** S5

D. Procedure and Spectral Data for the Synthesis of Compound **4** S6

E. Procedure and Spectral Data for the Synthesis of Compound **20** S7

F. Procedure and Spectral Data for the Synthesis of Compound **3** S8

G. Procedure and Spectral Data for the Synthesis of Compound **14** S8

H. Procedure and Spectral Data for the Synthesis of Compound **15** S9

I. Procedure and Spectral Data for the Synthesis of Compound **2** S11

J. Procedure and Spectral Data for the Synthesis of **Kakeromamide A (1)** S12

IV. Spectral Data for Synthesis of Analogue**s** S13

A. Spectral Data for Synthesis of Analogue **16** S13

B. Spectral Data for Synthesis of Analogue **17** S15

C. Spectral Data for Synthesis of Analogue **18** S18

D. Spectral Data for Synthesis of Analogue **19** S20

V. Procedures for Biological Tests S21

VI. Comparison of NMR Data and Spectra of Natural and Synthetic Kakeromamide A S23

VII. NMR Spectra for Compounds **6**, **10**, **5**, **4**, **20, 3, 14**, **15**, **2**, **SI-1**, **SI-2**, **SI-3**, **SI-4**, **16**, **SI-5**, **SI-6**, **SI-7**, **17**, **SI-8**, **SI-9**, **18**, **SI-10, 19** S25

I. General Information

All reactions were conducted in flame-dried or oven-dried glassware under an atmosphere of dry nitrogen or argon. Oxygen and/or moisture sensitive solids and liquids were transferred appropriately. Concentration of solutions *in vacuo* was accomplished using a rotary evaporator fitted with a water aspirator. Residual solvents were removed under high vacuum (0.1-0.2 mm Hg). All reaction solvents were purified before use: Tetrahydrofuran was distilled from sodium benzophenone. Toluene was distilled over molten sodium metal. Dichloromethane, dimethylformamide, 1,2-dimethoxyethane and diisoproylethylamine were distilled from CaH_2. Methanol was distilled from Mg/I_2. Flash column chromatography was performed using the indicated solvents on E. Qingdao silica gel 60 (230-400 mesh ASTM). TLC was carried out using pre-coated sheets (Qingdao silica gel 60-F250, 0.2 mm). Compounds were visualized with UV light, iodine, *p*-anisaldehyde stain, ceric ammonium molybdate stain, or phosphomolybdic acid in EtOH.

II. Spectroscopy, Spectrometry, and Data Collection

Proton nuclear magnetic resonance (1H NMR) spectra were recorded on Bruker DPX 300 MHz, Avance 400 MHz or AV 500 MHz spectrometers. Chemical shifts are reported in delta (δ) units, parts per million (ppm), relative to either a tetramethylsilane (TMS) internal standard or the signals due to the solvent. The following abbreviations are used to describe spin multiplicity: s = singlet, d = doublet, t = triplet, q = quartet, qn = quintet, m = multiplet, br = broad, dd = doublet of doublets, dt = doublet of triplets, dq = doublet of quartets, ddd = doublet of doublet of doublets; other combinations are derived from those listed above. AB quartet relationships are noted, but listed as a pair of doublets. Coupling constants (J) are reported in Hertz. Carbon-13 nuclear magnetic resonance (^{13}C NMR) spectra were completely heterodecoupled and measured at 125, 100, or 75 MHz. Chemical shifts are reported in delta (δ) units, ppm relative to the center of the triplet at 77.16 ppm for deuteriochloroform. Low- and high- resolution EI and ESI mass spectra were obtained using an AB QSTAR Elite mass spectrometer. Optical rotations were recorded on a Rudolph AutoPol-I polarimeter at 589 nm, 100 mm cell or 50 mm cell at 20 °C.

III. Experimental Details and Spectral Data for Total Synthesis of Kakeromamide A (1)

Synthesis of Compound 6:

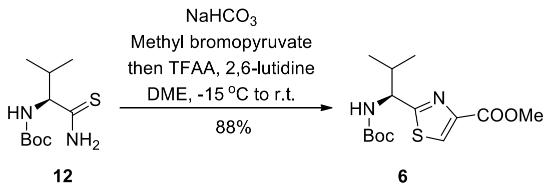

To a solution of thioamide **12** (0.50 g, 2.15 mmol) in anhydrous DME (13 mL) $NaHCO_3$ (1.44 g, 17.14 mmol) was added at -15 °C. The reaction mixture was stirred vigorously for 15 min at -15 °C, and methyl bromopyruvate (0.69 mL, 6.45 mmol) was added dropwise via syringe. The mixture was stirred at -15 °C for 30 min and then allowed to warm up to ambient temperature and stirred for additional 30 min. The reaction mixture was re-cooled to -15 °C and successively added dropwise trifluoroacetic anhydride (1.21 mL, 8.58 mmol) and 2,6-lutidine (2.12 mL, 18.3 mmol), and then allowed to warm to room temperature and stirred for additional 12 h. The reaction mixture was concentrated and the residue was partitioned between H_2O (25 mL) and EtOAc (50 mL). The phases were separated, and the aqueous phase was extracted with EtOAc (3 x 10 mL). The combined organic layers were dried over anhydrous Na_2SO_4 and concentrated under vacuum. The residue was purified by flash chromatography on silica gel using hexanes−EtOAc (5:1) as eluent to furnish compound **6** (594 mg, 88%) as a white solid; $[\alpha]_D^{24}$ = -57.6 (c 2.0, $CHCl_3$); mp 117−118 °C; ^{1}H NMR (400 MHz, $CDCl_3$) δ 8.06 (s, 1H), 5.29 (d, J = 9.0 Hz, 1H), 4.93 – 4.76 (m, 1H), 3.90 (s, 3H), 2.48 – 2.33 (m, 1H), 1.40 (s, 9H), 0.94 (d, J = 6.7 Hz, 3H), 0.86 (d, J = 7.3 Hz, 3H); ^{13}C NMR (100 MHz, $CDCl_3$): δ 173.5, 161.8, 155.4, 147.0, 127.1, 80.1, 58.0, 52.4, 33.2, 28.3, 19.4, 17.2; HRESIMS *m/z* 315.1382 $[M+H]^+$ (calcd. for $C_{14}H_{23}N_2O_4S^+$, 315.1373).

Synthesis of Compound 10:

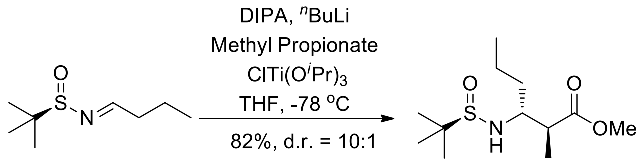

9 10

To a solution of i-Pr_2NH (2.18 mL, 15.43 mmol,) in THF (30 mL) at 0 °C was added n-Butyllithium (8.58 mL, 15.43 mmol, 2.39 M in Hexane) and stirred for 30 min. The solution was cooled to -78 °C before methyl propionate (1.66 mL, 17.13 mmol) was added via syringe and the reaction mixture was stirred for 30 min. To this solution was added $ClTi(O$i$-Pr)_3$ (18.86 mL, 18.86 mmol, 1 M in THF) dropwise via syringe and the resulting orange-colored enolate was stirred for 30 min, prior to addition of a solution of the *N*-sulfinyl imine **9** (1.5 g, 8.57 mmol) in THF (10 mL). The reaction was stirred at -78 °C for 12 hours, at which point TLC analysis indicated complete consumption of the starting material, and a saturated aqueous solution of NH_4Cl (30 mL) was added and the suspension was warmed up to ambient temperature and stirred for additional 1 hour. The mixture was filtered through a pad of celite and the filtrate was extracted with EtOAc (3 x 50 mL). The combined organic layers were washed with brine, dried over anhydrous

Na_2SO_4 and concentrated under vacuum. The residue was purified by flash chromatography on silica gel using hexanes−EtOAc (2:1) as eluent to furnish compound **10** (91% d.e., the major diastereomer isolated: 1.85 g, 82%,) as a yellow oil; $[\alpha]_D^{28}$ = -66.0 (c 1.0, $CHCl_3$); ^{1}H NMR (400 MHz, $CDCl_3$, major diatereomer) δ 4.06 (d, J = 8.8 Hz, 1H), 3.48 (s, 3H), 3.18 – 3.04 (m, 1H), 2.85 – 2.72 (m, 1H), 1.34 – 1.05 (m, 4H), 1.01 (s, 9H), 0.96 (d, J = 7.2 Hz, 3H), 0.68 (t, J = 7.1 Hz, 3H); ^{13}C NMR (100 MHz, $CDCl_3$, major diatereomer): δ 174.7, 58.9, 55.7, 51.4, 43.9, 33.5, 22.4, 19.1, 13.4, 12.9; HRESIMS m/z 264.1627 $[M+H]^+$ (calcd for $C_{12}H_{26}NO_3S^+$, 264.1628).

Synthesis of Compound 5:

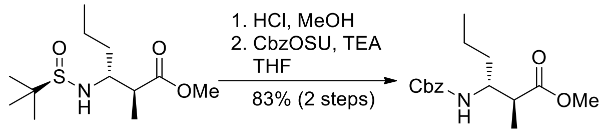

10 **5**

To a solution of compound **10** (303 mg, 1.15 mmol) in anhydrous MeOH (3 mL) was added HCl/MeOH solution (1M, 2.3 mL) dropwise via syringe at 0 °C. The reaction mixture was warmed to ambient temperature and stirred for 2 hours, at which point **10** had been consumed as judged by TLC analysis. Volatiles were removed under vacuum and the residue was dried under high vacuum for 2 hours and used in the next step without further purification.

To a solution of the above crude product in anhydrous THF (4 mL), CbzOSu (574 mg, 2.30 mmol) was added at 0 °C followed by TEA (0.64 mL, 4.60 mmol). The reaction mixture was warmed to ambient temperature and stirred for 24 hours, at which point the starting material had been consumed as judged by TLC analysis. The reaction mixture was diluted with EtOAc (40 mL), washed with a saturated aqueous solution of NH_4Cl (20 mL) and brine (20 mL), dried over anhydrous Na_2SO_4 and concentrated under vacuum. The residue was purified by flash chromatography on silica gel using hexanes−EtOAc (5:1) as eluent to furnish compound **5** (281 mg, 83% for 2 steps) as a colorless oil; $[\alpha]_D^{28}$ = +52.0 (c 1.0, $CHCl_3$); ^{1}H NMR (400 MHz, $CDCl_3$) δ 7.40 – 7.27 (m, 5H), 5.09 (s, 2H), 4.96 (d, J = 9.9 Hz, 1H), 3.93 – 3.83 (m, 1H), 3.67 (s, 3H), 2.65 (qd, J = 7.2, 5.0 Hz, 1H), 1.49 – 1.39 (m, 2H), 1.36 – 1.26 (m, 2H), 1.15 (d, J = 7.2 Hz, 3H), 0.91 (t, J = 6.5 Hz, 3H); ^{13}C NMR (100 MHz, $CDCl_3$) δ 174.9, 156.2, 136.7, 128.5, 128.1, 128.0, 66.6, 53.1, 51.7, 44.0, 34.1, 19.5, 13.8, 13.1; HRESIMS m/z 294.1692 $[M+H]^+$ (calcd for $C_{16}H_{24}NO_4^+$, 294.1700).

Synthesis of Compound 4:

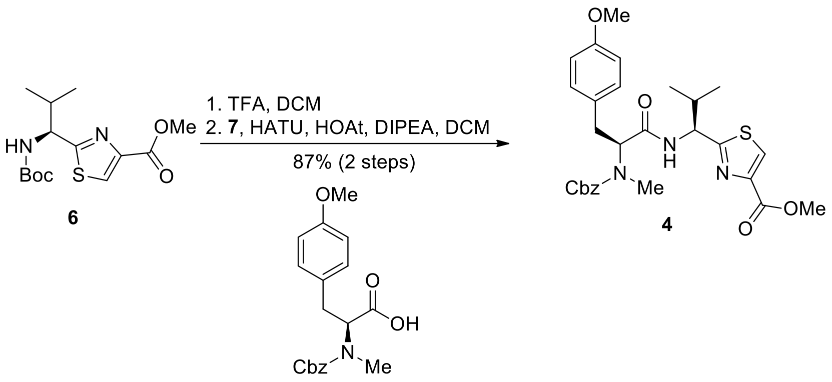

7

To a solution of compound **6** (314 mg, 1.0 mmol) in anhydrous DCM (3 mL) was added trifluoroacetic acid (1 mL, 13.5 mmol) at 0 °C. The reaction mixture was warmed to ambient temperature and stirred for 2 hours, at which point the starting material had been consumed as judged by TLC analysis. Volatiles were removed under vacuum and the residue was dried under high vacuum for 2 hours to afford the corresponding ammonium salt, which was used without further purification.

To a solution of the above ammonium salt and the known acid **7** (515 mg, 1.5 mmol) in anhydrous DCM (5 mL) was sequentially added HATU (950 mg, 2.5 mmol), HOAt (136 mg, 1.0 mmol) and DIPEA (0.83 mL, 5.0 mmol) at 0 °C. The reaction mixture was warmed up to ambient temperature and stirred for 12 hours, and then quenched with 10% aqueous solution of citric acid (5 mL). The reaction mixture was extracted with DCM (25 mL) and the organic layer was washed with a saturated aqueous solution of $NaHCO_3$ (5 mL) and brine (5 mL), dried over anhydrous Na_2SO_4 and concentrated under vacuum. The residue was purified by flash chromatography on silica gel using hexanes-EtOAc (2:1) as eluent to furnish compound **4** (469 mg, 87% for 2 steps) as a colorless oil; $[\alpha]_D^{30}$ = -72.8 (c 1.0, $CHCl_3$); ^1H NMR (500 MHz, CD_3CN, 60℃) δ 8.14 (s, 1H), 7.40 – 7.27 (m, 5H), 7.12 (d, *J* = 8.2 Hz, 2H), 6.95 (br, 1H), 6.80 (d, *J* = 8.2 Hz, 2H), 5.14 – 5.02 (m, 3H), 4.87 (dd, *J* = 9.5, 6.2 Hz, 1H), 3.88 (s, 3H), 3.76 (s, 3H), 3.21 (dd, *J* = 14.5, 6.2 Hz, 1H), 2.96 (dd, *J* = 14.5, 9.5 Hz, 1H), 2.88 (s, 3H), 2.34 (m, 1H), 0.92 (d, *J* = 3.5 Hz, 3H), 0.90 (d, *J* = 3.6 Hz, 3H); ^{13}C NMR (125 MHz, CD_3CN, 60°C) δ 173.84, 171.65, 162.89, 160.00, 147.93, 138.49, 131.31, 131.09, 129.74, 129.19, 128.97, 128.91, 118.16, 115.35, 68.36, 62.28, 58.40, 56.29, 52.83, 34.74, 33.80, 32.08, 20.04, 18.69; HRESIMS *m/z* 540.2163 $[M+H]^+$ (calcd. for $C_{28}H_{34}N_3O_6S^+$, 540.2163).

Synthesis of Compound 20:

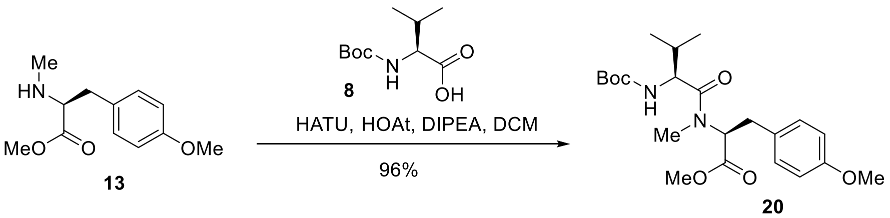

To a solution of amine **13** (357 mg, 1.6 mmol) and acid **8** (348 mg, 1.6 mmol) in anhydrous DCM (5 mL) was sequentially added HATU (1.22 g, 3.2 mmol), HOAt (218 mg, 1.6 mmol) and DIPEA (1.32 mL, 8.0 mmol) at 0 °C. The reaction mixture was warmed up to ambient temperature and stirred for 48 hours, and then quenched with 10% aqueous solution of citric acid (5 mL). The reaction mixture was extracted with DCM (25 mL) and the organic layer was washed with a saturated aqueous solution of $NaHCO_3$ (5 mL) and brine (5 mL), dried over anhydrous Na_2SO_4 and concentrated under vacuum. The residue was purified by flash chromatography on silica gel using hexanes-EtOAc (2:1) as eluent to furnish compound **20** (645 mg, 96%) as a colorless oil; ^{1}H NMR (500 MHz, $CDCl_3$) δ 7.10 (d, J = 8.6 Hz, 2H), 6.81 (d, J = 8.6 Hz, 2H), 5.33 – 5.28 (m, 1H), 5.09 (d, J = 9.3 Hz, 1H), 4.39 – 4.34 (m, 1H), 3.77 (s, 3H), 3.72 (s, 3H), 3.36 – 3.30 (m, 1H), 2.97 – 2.93 (m, 1H), 2.84 (s, 3H), 1.99 – 1.90 (m, 1H), 1.41 (s, 9H), 0.96 (d, J = 6.8 Hz, 3H), 0.90 (d, J = 6.8 Hz, 3H).

Synthesis of Compound 3:

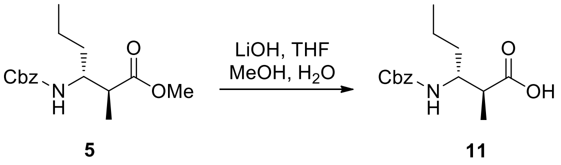

To a solution of compound **5** (110 mg, 0.28 mmol) in a mixture of THF (1 mL), MeOH (1 mL) and H_2O (0.5 mL) was added LiOH (35 mg, 0.84 mmol) at 0 °C. The reaction mixture was warmed up to ambient temperature and stirred for 10 hours, at which point the starting material had been consumed as judged by TLC analysis. Volatiles were removed under vacuum and to the residue was added Et_2O (1 mL). The solution was acidified with HCl/H_2O solution (1 M) to PH ~ 5 at °C and the aqueous phase was extracted with EtOAc (3 x 3 mL). The combined organic layers were washed with brine (5 mL), dried over anhydrous Na_2SO_4 and concentrated under vacuum. The residue was dried under high vacuum for 2 hours to afford the corresponding acid **11**, which was used without further purification.

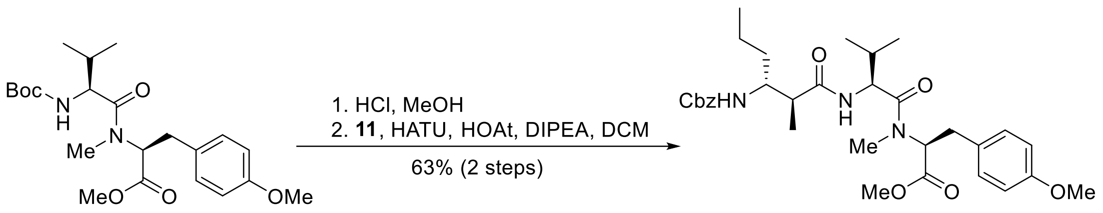

20 3

To a solution of compound **20** (118 mg, 0.28 mmol) in anhydrous MeOH (2 mL) was added HCl/MeOH solution (1.5M, 0.56 mL) dropwise at 0 °C. The reaction mixture was warmed to ambient temperature and stirred for 3 hours, at which point **20** had been consumed as judged by TLC analysis. Volatiles were removed under vacuum and the residue was dried under high vacuum for 2 hours and used in the next step without further purification.

To a solution of the above crude product and acid **11** in anhydrous DCM (2 mL) was sequentially added HATU (266 mg, 0.7 mmol), HOAt (38 mg, 0.28 mmol) and DIPEA (0.24 mL, 1.4 mmol) at 0 °C. The reaction mixture was warmed up to ambient temperature and stirred for 12 hours, then quenched with 10% aqueous solution of citric acid (4 mL). The reaction mixture was extracted with DCM (10 mL) and the organic layer was washed with a saturated aqueous solution of $NaHCO_3$ (4 mL) and brine (4 mL), dried over anhydrous Na_2SO_4 and concentrated under vacuum. The residue was purified by flash chromatography on silica gel using hexanes−EtOAc (1:1) as eluent to furnish compound **3** (102 mg, 63% for 2 steps) as a colorless oil; ^{1}H NMR (500 MHz, $CDCl_3$) δ 7.40 – 7.33 (m, 5H), 7.08 (d, J = 8.6 Hz, 2H), 6.78 (d, J = 8.6 Hz, 2H), 6.03 (d, J = 8.5 Hz, 1H), 5.63 (d, J = 8.2 Hz, 1H), 5.17 – 5.10 (m, 3H), 4.71 – 4.65 (m, 1H), 3.75 (s, 3H), 3.69 (s, 3H), 3.33 – 3.28 (m, 1H), 2.98 – 2.96 (m, 1H), 2.95 (s, 3H), 2.93 – 2.89 (m, 1H), 2.55 – 2.48 (m, 1H), 2.03 – 1.95 (m, 1H), 1.50 – 1.37 (m, 2H), 1.33 – 1.23 (m, 2H), 1.15 (t, J = 5.8 Hz, 3H), 0.96 (d, J = 6.8 Hz, 3H), 0.87 (d, J = 6.8 Hz, 6H).

Synthesis of Compound 14:

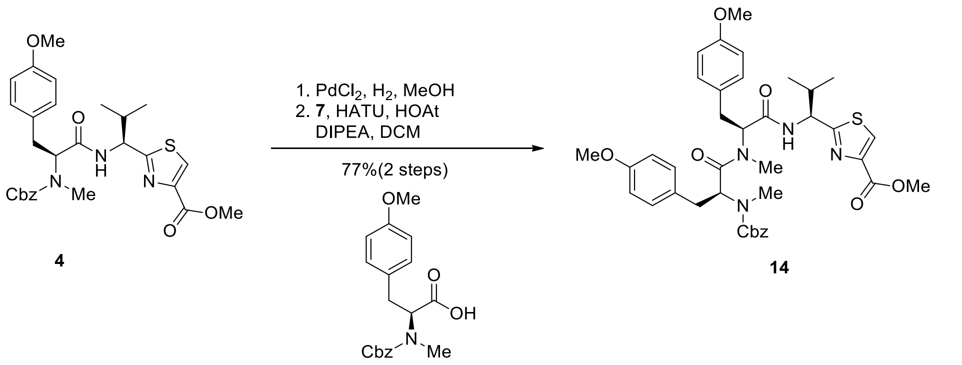

7

To a solution of compound **4** (431 mg, 0.8 mmol) in anhydrous MeOH (4 mL) was added $PdCl_2$ (71 mg, 0.4 mmol). The reaction flask was evacuated and purged with hydrogen three times. The reaction mixture

was stirred under a hydrogen atmosphere at ambient temperature for 10 h., at which point the starting material had been consumed as judged by TLC analysis. The catalyst was removed by filtration through a pad of celite and eluted with MeOH (10 mL). The filtrate was concentrated under vacuum and the residue was dried under high vacuum for 2 hours to afford the corresponding ammonium salt, which was used in the next step without further purification.

To a solution of the above ammonium salt and acid **7** (329 mg, 0.96 mmol) in anhydrous DCM (4 mL) was sequentially added HATU (760 mg, 2.0 mmol), HOAt (109 mg, 0.8 mmol) and DIPEA (0.66 mL, 4.0 mmol) at 0 °C. The reaction mixture was warmed up to ambient temperature and stirred for 12 hours, and then quenched with 10% aqueous solution of citric acid (4 mL). The reaction mixture was extracted with DCM (20 mL) and the organic layer was washed with a saturated aqueous solution of $NaHCO_3$ (4 mL) and brine (4 mL), dried over anhydrous Na_2SO_4 and concentrated under vacuum. The residue was purified by flash chromatography on silica gel using hexanes-EtOAc (2:1) as eluent to furnish compound **14** (450 mg, 77% for 2 steps) as a colorless oil. $[\alpha]_D^{25}$ = -178.0 (c 0.1, $CHCl_3$); ^{1}H NMR (500 MHz, DMSO-*d6,* 80°C) δ 8.34 (s, 1H), 7.95 (brs, 1H), 7.41 – 7.26 (m, 3H), 7.20 (d, *J* = 7.2 Hz, 2H), 7.09 (d, *J* = 8.1 Hz, 2H), 7.05 – 6.96 (m, 2H), 6.80 – 6.72 (m, 4H), 5.46 – 5.22 (m, 1H), 5.19 – 5.02 (m, 1H), 5.01 – 4.93 (m, 2H), 4.93 – 4.83 (m, 1H), 3.84 (s, 3H), 3.72 (s, 3H), 3.69 (s, 3H), 3.15 (dd, *J* = 14.6, 5.3 Hz, 1H), 2.93 (s, 3H), 2.90 – 2.78 (m, 1H), 2.85 (s, 3H), 2.75 (dd, *J* = 14.6, 8.4 Hz, 1H), 2.46 – 2.35 (m, 1H), 2.36 – 2.25 (m, 1H), 0.91 (d, *J* = 6.7 Hz, 3H), 0.88 (d, *J* = 6.7 Hz, 3H); ^{13}C NMR (125 MHz, DMSO-*d6*) (Mixture of conformers) δ 173.5, 172.6, 171.1, 171.0, 170.8, 170.5, 161.7, 161.7, 158.5, 158.5, 158.3, 158.2, 155.6, 155.0, 146.0, 137.4, 136.9, 130.7, 130.2, 130.2, 130.0, 129.8, 129.7, 129.7, 129.6, 129.4, 128.8, 128.7, 128.6, 128.3, 128.2, 128.1, 128.0, 127.6, 127.2, 114.5, 114.2, 114.1, 114.1, 114.0, 79.7, 69.0, 67.5, 67.0, 66.6, 61.8, 57.9, 57.6, 57.5, 57.1, 57.1, 56.3, 55.5, 55.5, 55.4, 55.4, 55.4, 54.9, 52.4, 34.1, 34.1, 32.5, 32.3, 31.5, 31.2, 30.1, 29.1, 28.7, 19.9, 19.8, 18.9; HRESIMS *m/z* 731.3115 $[M+H]^+$ (calcd for $C_{39}H_{47}N_4O_8S^+$, 731.3109).

Synthesis of Compound 15:

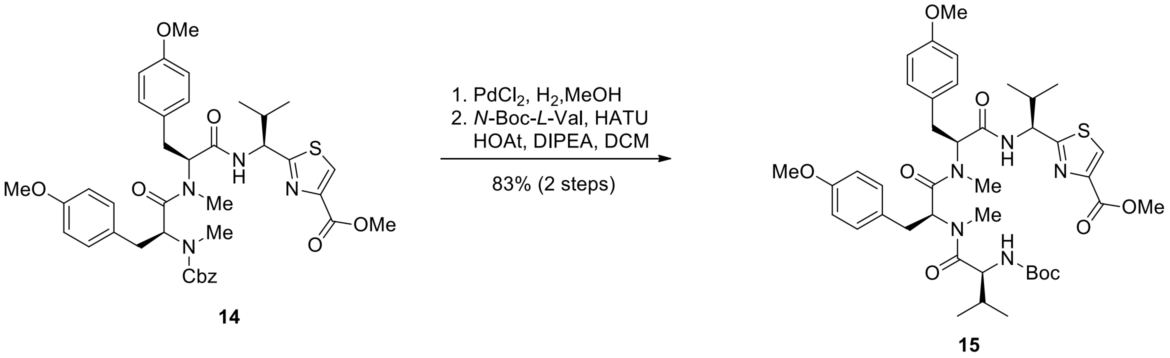

To a solution of compound **14** (438 mg, 0.6 mmol) in anhydrous MeOH (4 mL) was added $PdCl_2$ (53 mg, 0.3 mmol). The reaction flask was evacuated and purged with hydrogen three times and then the reaction was stirred under a hydrogen atmosphere at ambient temperature for 10 hours, at which point the starting material had been consumed as judged by TLC analysis. The catalyst was removed by filtration through a pad of celite and eluted with MeOH (10 mL). The filtrate was concentrated under vacuum and the residue was dried under high vacuum for 2 hours to afford the corresponding ammonium salt, which was used in the next step without further purification.

To a solution of above ammonium salt and *N*-Boc-*L*-Val (195 mg, 0.9 mmol) in anhydrous DCM (4 mL) was sequentially added HATU (570 mg, 1.5 mmol), HOAt (82 mg, 0.6 mmol) and DIPEA (0.50 mL, 3.0 mm) at 0 °C. The reaction mixture was warmed up to ambient temperature and stirred for 12 hours, and then quenched with 10% aqueous solution of citric acid (4 mL). The reaction mixture was extracted with DCM (20 mL) and the organic layer was washed with a saturated aqueous solution of $NaHCO_3$ (4 mL) and brine (4 mL), dried over anhydrous Na_2SO_4 and concentrated under vacuum. The residue was purified by flash chromatography on silica gel using hexanes−EtOAc (2:1) as eluent to furnish compound **15** (396 mg, 83% for 2 steps) as a colorless oil; $[\alpha]_D^{25}$ = -208.0 (c 0.1, $CHCl_3$; ^1H NMR (500 MHz, DMSO-*d6*) (Mixture of conformers) δ 8.58 (d, J = 8.5 Hz, 1H), 8.45 (s, 1H), 7.15 (d, J = 8.6 Hz, 2H), 7.01 (d, J = 8.4 Hz, 2H), 6.83 (d, J = 8.4 Hz, 2H), 6.71 (d, J = 8.6 Hz, 2H), 6.36 (d, J = 9.1 Hz, 1H), 5.51 (dd, J = 12.2, 4.8 Hz, 1H), 5.38 – 5.31 (m, 1H), 4.92 (dd, J = 8.5, 7.1 Hz, 1H), 3.96 (dd, J = 9.1, 6.4 Hz, 1H), 3.82 (s, 3H), 3.73 (s, 3H), 3.67 (s, 3H), 3.11 (dd, J = 14.6, 4.8 Hz, 1H), 3.00 (dd, J = 14.6, 7.1 Hz, 1H), 2.92 (dd, J = 12.2, 6.0 Hz, 1H), 2.89 – 2.85 (m, 1H), 2.73 (s, 3H), 2.30 (s, 3H), 2.34 – 2.23 (m, 1H), 1.62 – 1.52 (m, 1H), 1.36 (s, 9H), 0.88 (d, J = 6.7 Hz, 3H), 0.83 (d, J = 6.8 Hz, 3H), 0.69 (d, J = 7.2 Hz, 3H), 0.68 (d, J = 7.4 Hz, 3H); ^{13}C NMR (125 MHz, DMSO-*d6*) (Mixture of conformers) δ 173.8, 171.8, 171.4, 171.0, 162.0, 158.9, 158.5, 156.3, 146.3, 130.9, 130.4, 130.0, 129.7, 114.5, 114.3, 78.9, 69.3, 57.3, 57.2, 56.7, 56.0, 55.9, 55.7, 55.7, 52.8, 40.5, 34.4, 34.2, 32.9, 32.8, 31.4, 30.4, 30.3, 30.2, 28.9, 20.4, 20.1, 19.0, 17.9; HRESIMS *m/z* 796.3956 $[M+H]^+$ (calcd for $C_{41}H_{58}N_5O_9S^+$, 796.3950).

Synthesis of Compound 2:

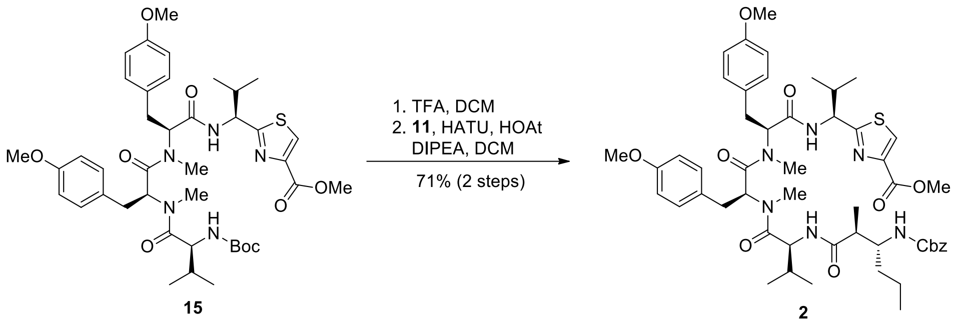

To a solution of compound **15** (318 mg, 0.4 mmol) in anhydrous DCM (3 mL) was added trifluoroacetic acid (1 mL, 13.5 mmol) at 0 °C. The reaction mixture was warmed up to ambient temperature and stirred for 2 hours, at which point the starting material had been consumed as judged by TLC analysis. Volatiles were removed under vacuum and the residue was dried under high vacuum for 2 hours to afford the corresponding ammonium salt, which was used in the next step without further purification.

To a solution of above ammonium salt and acid **11** (168 mg, 0.6 mmol) in anhydrous DCM (4 mL) was sequentially added HATU (380 mg, 1.0 mmol), HOAt (54 mg, 0.4 mmol) and DIPEA (0.33 mL, 2.0 mmol) at 0 °C. The reaction mixture was warmed up to ambient temperature and stirred for 12 hours, then quenched with 10% aqueous solution of citric acid (4 mL). The reaction mixture was extracted with DCM (20 mL) and the organic layer was washed with a saturated aqueous solution of $NaHCO_3$ (4 mL) and brine (4 mL), dried over anhydrous Na_2SO_4 and concentrated under vacuum. The residue was purified by flash chromatography on silica gel using hexanes−EtOAc (1:1) as eluent to furnish compound **2** (272 mg, 71% for 2 steps) as a colorless oil; $[\alpha]_D^{25}$ = -126.0 (c 0.1, $CHCl_3$); ^1H NMR (500 MHz, DMSO-d_6) (Mixture of conformers) δ 8.54 (d, J = 8.5 Hz, 1H), 8.45 (s, 1H), 8.08 (d, J = 8.9 Hz, 1H), 7.40 – 7.26 (m, 5H), 7.15 (d, J = 8.2 Hz, 2H), 7.03 (d, J = 8.6 Hz, 2H), 6.83 (d, J = 8.2 Hz, 2H), 6.73 (d, J = 8.6 Hz, 2H), 6.71 – 6.65 (m, 1H), 5.50 (dd, J = 12.0, 4.8 Hz, 1H), 5.35 (dd, J = 8.9, 4.7 Hz, 1H), 5.01 (s, 2H), 4.91 (dd, J = 8.5, 6.9 Hz, 1H), 4.32 – 4.21 (m, 1H), 3.82 (s, 3H), 3.74 (s, 3H), 3.71 – 3.68 (m, 1H), 3.67 (s, 3H), 3.54 (dd, J = 9.2, 4.8 Hz, 1H), 3.16 – 3.04 (m, 2H), 2.98 – 2.89 (m, 1H), 2.74 (s, 3H), 2.41 – 2.33 (m, 1H), 2.31 (s, 3H), 2.29 – 2.21 (m, 1H), 1.84 – 1.73 (m, 1H), 1.28 – 1.09 (m, 4H), 0.93 (d, J = 6.7 Hz, 3H), 0.83 (d, J = 6.6 Hz, 3H), 0.80 (d, J = 6.7 Hz, 3H), 0.77 (d, J = 6.6 Hz, 3H), 0.72 (t, J = 7.1 Hz, 3H), 0.71 (d, J = 6.5 Hz, 3H); ^{13}C NMR (125 MHz, DMSO-d_6) (Mixture of conformers) δ 175.2, 173.8, 171.6, 171.3, 170.6, 162.0, 158.9, 158.5, 157.2, 146.3, 138.3, 130.8, 130.6, 130.5, 129.9, 129.7, 129.1, 128.5, 128.4, 128.3, 114.5, 114.4, 65.8, 57.4, 57.1, 56.0, 55.9, 55.7, 54.4, 53.6, 52.8, 45.5, 35.7, 34.7, 34.2, 32.8, 31.4, 30.4, 30.2, 20.3, 20.0, 19.6, 18.8, 18.6, 16.3, 14.5; HRESIMS m/z 957.4784 $[M+H]^+$ (calcd for $C_{51}H_{69}N_6O_{10}S^+$, 957.4790).

Synthesis of kakeromamide A (1):

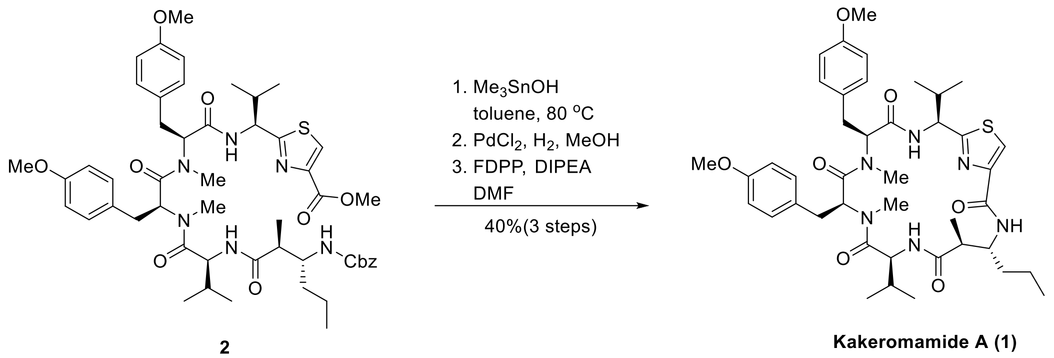

To a solution of **2** (30 mg, 0.031 mmol) in anhydrous toluene (1.5 mL) was added Me_3SnOH (28 mg, 0.155 mmol) and the reaction mixture was heated to 80 °C and stirred for 10 hours, at which point the starting material had been consumed as judged by TLC analysis. The reaction mixture was cooled to ambient temperature, quenched with a saturated aqueous solution of NH_4Cl (3 mL) and stirred for 10 min. and then extracted with EtOAc (3 x 5 mL). The combined organic layers were washed with brine (5 mL), dried over anhydrous Na_2SO_4 and concentrated under vacuum. The residue was dried under high vacuum for 2 hours to afford the corresponding acid, which was used in next step without further purification.

To a solution of the above acid in anhydrous MeOH (1.5 mL) was added $PdCl_2$ (3 mg, 0.016 mmol). The reaction flask was evacuated and purged with hydrogen three times. The reaction mixture was stirred under a hydrogen atmosphere at ambient temperature for 10 hours, at which point the starting material had been consumed as judged by TLC analysis. The catalyst was removed by filtration through a pad of celite and eluted with MeOH (5 mL). The filtrate was concentrated under vacuum and the residue was dried under high vacuum for 2 hours to afford the corresponding ammonium salt, which was used in the next step without further purification.

To a solution of the above ammonium salt in anhydrous DMF (31 mL) was added FDPP (119 mg, 0.31 mmol) and DIPEA (0.10 mL, 0.62 mmol) at 0 °C. The reaction mixture was warmed up to ambient temperature and stirred for 48 hours. Volatiles were removed under vacuum and the residue was dissolved in EtOAc (20 mL) and 10% aqueous solution of citric acid (4 mL) was added. The organic layer was washed with a saturated aqueous solution of $NaHCO_3$ (4 mL) and brine (4 mL), dried over anhydrous Na_2SO_4 and concentrated under vacuum. The residue was purified by flash chromatography on silica gel using hexanes−EtOAc (3:2) as eluent to furnish kakeromamide A (**1**) (10 mg, 40% for 3 steps) as an amorphous solid; $[\alpha]_D^{22}$ = +6.15 (c 0.065, MeOH); ^{1}H NMR (400 MHz, CD_3CN) δ 8.60 (d, J = 9.0 Hz, 1H), 8.51 (d, J = 10.3 Hz, 1H), 8.01 (s, 1H), 6.99 (d, J = 8.6 Hz, 2H), 6.88 (d, J = 8.6 Hz, 2H), 6.77 (d, J = 8.7 Hz, 2H), 6.55 (d, J = 8.7 Hz, 2H), 6.53 (d, J = 4.7 Hz, 1H), 5.53 (dd, J = 11.3, 4.7 Hz, 1H), 5.33 (dd, J = 9.0, 5.4 Hz, 1H), 5.25 (dd, J = 9.7, 5.2 Hz, 1H), 4.29 (dd, J = 9.8, 7.1 Hz, 1H), 4.12 – 4.02 (m, 1H), 3.73 (s, 3H), 3.48

(s, 3H), 3.04 (s, 3H), 2.96 (dd, J = 14.4, 5.1 Hz, 1H), 2.85 (s, 3H bv), 2.69 (dd, J = 16.3, 11.3 Hz, 1H), 2.65 – 2.56 (m, 2H), 2.06 – 1.97 (m, 1H), 1.84 – 1.74 (m, 1H), 1.74 – 1.64 (m, 1H), 1.52 – 1.40 (m, 1H), 1.37 (dd, J = 16.3, 4.6 Hz, 1H), 1.32 – 1.20 (m, 1H), 1.09 (d, J = 7.0 Hz, 3H), 1.14 – 1.00 (m, 1H), 0.96 (t, J = 7.3 Hz, 3H), 0.95 (d, J = 6.8 Hz, 3H), 0.89 (d, J = 6.6 Hz, 3H), 0.79 (d, J = 6.9 Hz, 3H), 0.78 (d, J = 6.8 Hz, 3H); ^{13}C NMR (100 MHz, CD_3CN) δ 177.0, 173.8, 173.3, 170.2, 170.0, 161.6, 159.7, 159.4, 150.5, 131.5, 131.1, 130.3, 130.1, 123.8, 115.3, 115.0, 63.9, 57.4, 57.4, 56.1, 55.8, 53.0, 52.4, 44.7, 36.9, 34.7, 33.5, 32.1, 32.1, 31.8, 29.9, 21.0, 20.4, 20.4, 19.0, 18.0, 14.8, 14.5; HRESIMS m/z 791.4161 [M+H]$^+$ (calcd for $C_{42}H_{59}N_6O_7S^+$, 791.4160).

IV. Spectral Data for Synthesis of Analogues

Synthesis of Analogue 16:

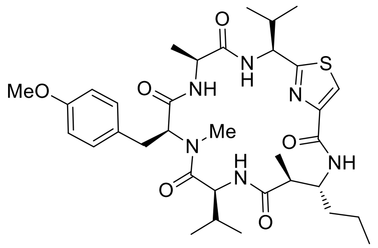

16

Synthesis of analogue **16** was performed in an identical manner as outlined for kakeromamide A (**1**).

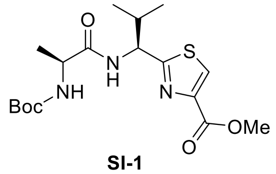

SI-1

The corresponding dipeptide **SI-1** was synthesized on a 1 mmol scale and obtained as a colorless oil (331 mg, 86% for 2 steps); $[\alpha]_D^{29}$ = -203.6 (c 1.0, $CHCl_3$); ^{1}H NMR (400 MHz, $CDCl_3$) δ 8.06 (s, 1H), 7.16 (br, 1H), 5.18 (dd, J = 8.8, 5.8 Hz, 1H), 5.08 (d, J = 7.4 Hz, 1H), 4.26 – 4.15 (m, 1H), 3.90 (s, 3H), 2.56 – 2.40 (m, 1H), 1.41 (s, 9H), 1.34 (d, J = 7.1 Hz, 3H), 0.92 (d, J = 6.7 Hz, 3H), 0.89 (d, J = 6.8 Hz, 3H); ^{13}C NMR (100 MHz, $CDCl_3$) δ 172.6, 161.8, 155.8, 147.0, 127.1, 80.4, 56.5, 52.4, 50.1, 33.0, 29.7, 28.3, 19.4, 17.3; HRESIMS m/z 386.1736 [M+H]$^+$ (calcd for $C_{17}H_{28}N_3O_5S^+$, 386.1744).

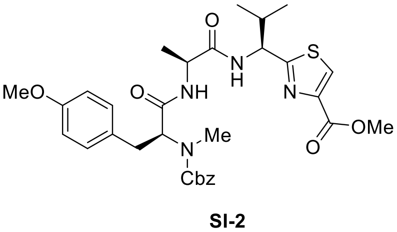

SI-2

The corresponding tripeptide **SI-2** was synthesized on a 0.8 mmol scale and obtained as a colorless oil (381 mg, 78% for 2 steps); $[\alpha]_D^{25}$ = -151.2 (c 0.5, $CHCl_3$); ^{1}H NMR (500 MHz, DMSO-*d6*, 80 °C) δ 8.33 (s, 1H), 8.07 (d, *J* = 8.4 Hz, 1H), 7.65 (d, *J* = 7.6 Hz, 1H), 7.38 – 7.26 (m, 3H), 7.27 – 7.21 (m, 2H), 7.13 (d, *J* = 8.7 Hz, 2H), 6.80 (d, *J* = 8.7 Hz, 2H), 5.07 – 4.95 (m, 3H), 4.84 (dd, *J* = 10.2, 5.4 Hz, 1H), 4.50 – 4.41 (m, 1H), 3.84 (s, 3H), 3.74 (s, 3H), 3.16 (dd, *J* = 14.7, 5.4 Hz, 1H), 2.91 – 2.85 (m, 1H), 2.81 (s, 3H), 2.38 – 2.27 (m, 1H), 1.28 (d, *J* = 7.0 Hz, 3H), 0.96 (d, *J* = 6.8 Hz, 3H), 0.93 (d, *J* = 6.8 Hz, 3H); ^{13}C NMR (125 MHz, DMSO-*d6*) (Mixture of conformers) δ 174.2, 173.2, 170.8, 162.0, 158.6, 146.4, 137.7, 130.7, 130.5, 130.5, 129.6, 129.0, 128.4, 127.8, 114.5, 80.0, 66.9, 60.4, 57.3, 55.8, 52.7, 49.1, 34.7, 34.3, 32.9, 31.5, 20.1, 18.8, 18.7; HRESIMS *m/z* 611.2539 $[M+H]^+$ (calcd for $C_{31}H_{39}N_4O_7S^+$, 611.2534).

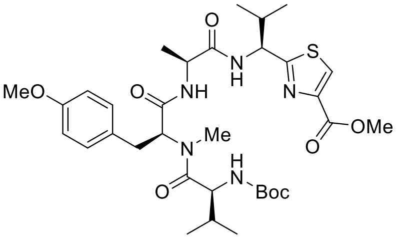

SI-3

The corresponding tetrapeptide **SI-3** was synthesized on a 0.6 mmol scale and obtained as a colorless oil (336 mg, 83% for 2 steps); $[\boldsymbol{\alpha}]_{\mathbf{D}}^{\mathbf{24}}$: -154.0 (*c* = 0.1, $CHCl_3$); ^{1}H NMR (400 MHz, DMSO-*d6*) (Mixture of conformers) δ 8.61 – 8.45 (m, 1H), 8.43 (s, 1H), 7.99 – 7.78 (m, 1H), 7.16 (d, *J* = 8.5 Hz, 1H), 7.10 (d, *J* = 8.5 Hz, 1H), 6.83 (d, *J* = 8.5 Hz, 1H), 6.76 (d, *J* = 8.6 Hz, 1H), 6.60 (d, *J* = 8.8 Hz, 1H), 5.32 – 5.20 (m, 1H), 4.94 (dd, *J* = 8.3, 6.6 Hz, 1H), 4.50 – 4.33 (m, 1H), 4.19 – 3.98 (m, 1H), 3.82 (s, 3H), 3.68 (s, 3H), 3.21 – 3.06 (m, 1H), 2.97 (s, 2H), 2.87 – 2.81 (m, 1H), 2.77 (s, 1H), 2.37 – 2.22 (m, 1H), 1.93 – 1.77 (m, 1H), 1.33 (s, 9H), 1.22 (d, *J* = 7.1 Hz, 3H), 0.90 (d, *J* = 6.6 Hz, 3H), 0.87 (d, *J* = 6.8 Hz, 3H), 0.82 (d, *J* = 3.9 Hz, 2H), 0.80 (d, *J* = 3.8 Hz, 2H), 0.57 (d, *J* = 6.7 Hz, 1H), 0.44 (d, *J* = 6.7 Hz, 1H); ^{13}C NMR (100 MHz, DMSO-*d6*) (Mixture of conformers) δ 174.4, 174.4, 173.5, 173.2, 173.0, 172.8, 170.5, 169.8, 162.1, 159.0, 158.5, 156.9, 156.2, 146.5, 131.2, 130.5, 130.4, 129.8, 114.7, 114.4, 80.2, 80.1, 79.9, 79.6, 79.2, 78.8, 61.8, 57.6, 57.4, 57.3, 56.3, 56.0, 55.7, 52.9, 49.3, 49.1, 34.7, 33.8, 33.0, 32.9, 32.2, 30.9, 30.3, 29.7, 29.0, 28.8, 20.2, 20.2, 19.9, 19.1, 19.0, 18.8, 18.3; HRESIMS *m/z* 676.3375 $[M+H]^+$ (calcd for $C_{33}H_{50}N_5O_8S^+$, 676.3375).

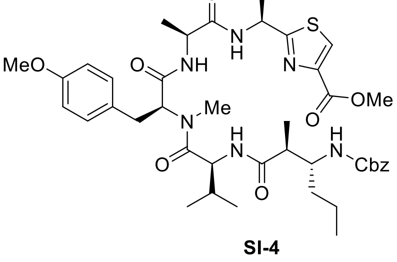

SI-4

The corresponding pentapeptide **SI-4** was synthesized on a 0.4 mmol scale and obtained as a colorless oil (268 mg, 80% for 2 steps); $[\alpha]_D^{23}$: -68.0 (c = 0.1, $CHCl_3$); ^{1}H NMR (500 MHz, DMSO-*d6*) (Mixture of conformers) δ 8.52 – 8.39 (m, 2H), 8.26 – 7.78 (m, 2H), 7.39 – 7.25 (m, 5H), 7.19 – 6.98 (m, 3H), 6.86 – 6.71 (m, 2H), 5.30 – 4.83 (m, 4H), 4.55 – 4.24 (m, 2H), 3.82 (s, 3H), 3.73 – 3.63 (m, 3H), 3.61 – 3.48 (m, 1H), 3.14 (dd, J = 14.6, 6.6 Hz, 1H), 2.99 (s, 2H), 2.76 (s, 1H), 2.83 – 2.67 (m, 1H), 2.47 – 2.21 (m, 2H), 2.04 – 1.86 (m, 1H), 1.32 – 1.09 (m, 8H), 0.93 (d, J = 6.9 Hz, 3H), 0.88 (d, J = 6.7 Hz, 3H), 0.86 (d, J = 6.9 Hz, 3H), 0.83 (t, J = 7.2 Hz, 3H), 0.78 – 0.70 (m, 3H), 0.64 (d, J = 6.8 Hz, 1H), 0.48 (d, J = 6.8 Hz, 1H); ^{13}C NMR (125 MHz, DMSO-*d6*) (Mixture of conformers) δ175.6, 174.7, 173.8, 172.7, 172.7, 169.8, 161.7, 158.6, 158.2, 156.8, 146.1, 146.0, 138.0, 130.8, 130.1, 130.0, 129.2, 128.8, 128.1, 127.9, 127.9, 114.4, 114.1, 65.5, 61.6, 60.2, 57.6, 56.9, 56.9, 55.6, 55.4, 54.3, 54.0, 53.2, 52.4, 49.1, 48.8, 45.2, 44.9, 35.3, 33.4, 32.6, 32.5, 31.8, 31.7, 30.6, 30.4, 29.7, 29.4, 21.2, 19.8, 19.7, 19.7, 19.6, 19.3, 19.2, 18.7, 18.6, 18.4, 18.4, 18.1, 16.0, 14.6, 14.2, 14.1; HRESIMS *m/z* 837.4212 $[M+H]^+$ (calcd for $C_{43}H_{61}N_6O_9S^+$, 837.4215).

Analogue **16** was synthesized on a 0.03 mmol scale and obtained as an amorphous solid (11 mg, 52% for 3 steps); $[\alpha]_D^{27}$ = -107.9 (c 0.28, $CHCl_3$); ^{1}H NMR (400 MHz, $CDCl_3$) δ 8.12 (d, J = 8.7 Hz, 1H), 7.97 (s, 1H), 7.88 (d, J = 9.2 Hz, 1H), 7.12 (d, J = 8.6 Hz, 2H), 6.87 (d, J = 8.6 Hz, 2H), 5.78 (d, J = 4.6 Hz, 1H), 5.76 (d, J = 3.0 Hz, 1H), 5.04 (dd, J = 9.2, 7.3 Hz, 1H), 4.63 (t, J = 9.2 Hz, 1H), 4.25 – 4.14 (m, 1H), 4.07 – 3.99 (m, 1H), 3.93 (t, J = 7.9 Hz, 1H), 3.80 (s, 3H), 3.29 – 3.14 (m, 2H), 3.10 (s, 3H), 2.76 – 2.65 (m, 1H), 2.24 – 2.13 (m, 1H), 1.91 – 1.80 (m, 1H), 1.63 – 1.54 (m, 1H), 1.50 – 1.42 (m, 1H), 1.39 (d, J = 7.3 Hz, 3H), 1.36 – 1.32 (m, 1H), 1.31 – 1.27 (m, 1H), 1.20 (d, J = 7.0 Hz, 3H), 1.04 (d, J = 6.8 Hz, 3H), 0.99 (d, J = 6.7 Hz, 3H), 0.94 (d, J = 6.7 Hz, 3H), 0.91 (d, J = 6.7 Hz, 3H), 0.87 (t, J = 7.1 Hz, 3H); ^{13}C NMR (100 MHz, $CDCl_3$) δ 173.2, 172.4, 172.3, 170.2, 169.4, 161.3, 158.9, 149.2, 129.9, 128.2, 122.5, 114.5, 65.7, 57.0, 55.3, 54.8, 52.7, 49.9, 44.2, 37.8, 34.2, 34.0, 30.7, 29.7, 29.7, 20.0, 19.6, 19.5, 18.7, 16.9, 13.9, 13.8; HRESIMS *m/z* 671.3586 $[M+H]^+$ (calcd for $C_{34}H_{51}N_6O_6S^+$, 671.3585).

Synthesis of Analogue 17:

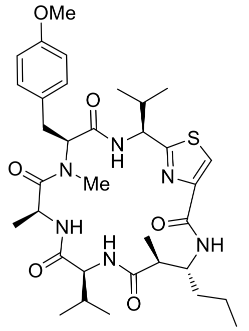

17

Synthesis of analogue **17** was performed in an identical manner as outlined for kakeromamide A (**1**).

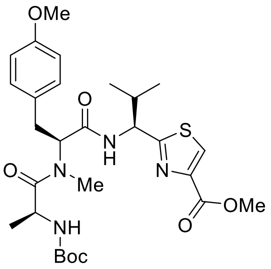

SI-5

The corresponding tripeptide **SI-5** was synthesized on a 0.8 mmol scale and obtained as a white solid (341 mg, 74% for 2 steps); $[\alpha]_D^{24}$ = -80.0 (c 0.49, $CHCl_3$); mp 50.6 – 51.6 °C; ^1H NMR (500 MHz, $CDCl_3$) (Mixture of conformers) δ 8.49 – 6.89 (m, 4H), 6.77 – 6.60 (m, 2H), 5.37 – 5.16 (m, 1H), 5.15 – 4.64 (m, 2H), 4.56 – 4.04 (m, 1H), 3.84 (s, 3H), 3.66 (s, 3H), 3.25 – 3.08 (m, 1H), 3.02 – 2.80 (m, 4H), 2.45 – 2.29 (m, 1H), 1.41 – 1.23 (m, 9H), 1.21 – 0.86 (m, 3H), 0.87 – 0.78 (m, 3H), 0.77 – 0.24 (m, 3H); ^{13}C NMR (125 MHz, $CDCl_3$) (Mixture of conformers) δ 174.1, 174.0, 172.9, 172.4, 169.6, 169.3, 161.8, 161.7, 158.7, 158.4, 156.1, 155.0, 147.0, 146.5, 130.4, 130.0, 129.7, 129.5, 128.5, 127.4, 127.0, 114.4, 114.0, 80.3, 79.6, 62.8, 60.3, 59.1, 57.8, 56.4, 55.3, 55.1, 52.3, 52.2, 46.7, 45.2, 33.3, 33.0, 32.7, 32.7, 31.7, 29.7, 28.3, 28.2, 19.8, 19.4, 18.7, 18.5, 17.2, 16.5, 14.2; HRESIMS *m/z* 577.2684 $[M+H]^+$ (calcd for $C_{28}H_{41}N_4O_7S^+$, 577.2690).

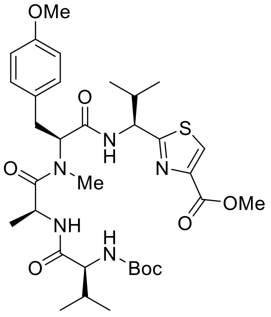

SI-6

The corresponding tetrapeptide **SI-6** was synthesized on a 0.5 mmol scale and obtained as a white solid (287 mg, 85% for 2 steps); $[\boldsymbol{\alpha}]_D^{24}$: -99.2 (c 0.52, $CHCl_3$); mp 63.3 – 64.6 °C; ^1H NMR (500 MHz, $CDCl_3$) (Mixture of conformers) δ 8.73 – 6.92 (m, 5H), 6.83 – 6.67 (m, 2H), 5.37 – 5.13 (m, 2H), 5.12 – 4.89 (m, 1H), 4.86 – 4.20 (m, 1H), 4.02 – 3.80 (m, 4H), 3.72 (s, 3H), 3.34 – 2.71 (m, 5H), 2.46 – 2.27 (m, 1H), 2.13 – 1.92 (m, 1H), 1.51 – 1.34 (m, 9H), 1.30 – 0.31 (m, 15H); ^{13}C NMR (125 MHz, $CDCl_3$) (Mixture of conformers) δ 173.3, 173.2, 172.9, 172.5, 172.2, 170.8, 169.6, 169.3, 161.9, 161.8, 158.8, 158.5, 155.8, 147.0, 146.7, 130.5, 130.0, 129.7, 129.4, 128.3, 127.3, 127.1, 114.4, 114.0, 79.9, 62.7, 59.7, 59.5, 59.0, 57.4, 56.5, 55.3, 55.2, 52.4, 45.7, 44.6, 38.6, 33.5, 33.1, 32.9, 32.7, 31.8, 31.1, 29.5, 28.3, 19.6, 19.5, 19.3, 19.2, 18.4, 18.3, 18.1, 17.6, 17.4, 16.2; HRESIMS *m/z* 676.3377 $[M+H]^+$ (calcd for $C_{33}H_{50}N_5O_8S^+$, 676.3375).

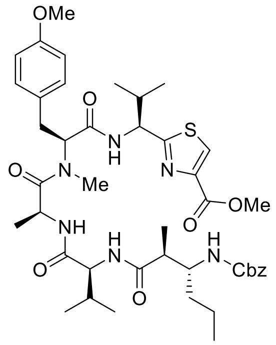

SI-7

The corresponding pentapeptide **SI-7** was synthesized on a 0.4 mmol scale and obtained as a colorless oil (274 mg, 82% for 2 steps); $[\boldsymbol{\alpha}]_{\mathbf{D}}^{\mathbf{24}}$: -137.7 (*c* = 0.26, $CHCl_3$); ^{1}H NMR (300 MHz, $CDCl_3$) (Mixture of conformers) δ 8.86 – 7.11 (m, 8H), 7.11 – 6.50 (m, 5H), 5.25 – 4.97 (m, 4H), 4.98 – 4.75 (m, 1H), 4.32 (dd, *J* = 12.4, 6.7 Hz, 1H), 4.23 – 3.18 (m, 8H), 3.14 – 2.83 (m, 4H), 2.68 – 2.53 (m, 1H), 2.52 – 2.27 (m, 1H), 2.14 – 2.02 (m, 2H), 1.57 – 1.37 (m, 3H), 1.36 – 1.20 (m, 2H), 1.22 – 1.07 (m, 3H), 1.00 – 0.78 (m, 15H), 0.38 (d, *J* = 6.5 Hz, 2H); ^{13}C NMR (75 MHz, $CDCl_3$) (Mixture of conformers) δ 174.7, 173.2, 173.2, 172.5, 172.4, 171.5, 170.4, 169.7, 169.2, 162.0, 161.8, 158.7, 158.4, 156.5, 146.9, 146.6, 136.5, 136.5, 130.5, 129.9, 129.3, 128.5, 128.3, 128.1, 128.0, 127.8, 127.5, 127.2, 114.3, 114.0, 66.7, 62.5, 58.4, 58.1, 56.9, 56.5, 55.3, 55.2, 53.5, 52.6, 52.5, 45.7, 45.4, 45.2, 44.4, 34.6, 34.3, 33.3, 33.1, 33.0, 32.7, 31.9, 31.1, 30.8, 29.7, 29.5, 19.7, 19.6, 19.4, 19.3, 19.3, 19.0, 18.8, 18.2, 18.1, 17.4, 16.1, 14.2, 13.8, 13.7; HRESIMS *m/z* 837.4224 $[M+H]^+$ (calcd for $C_{43}H_{61}N_6O_9S^+$, 837.4215).

Analogue **17** was synthesized on a 0.03 mmol scale and obtained as an amorphous solid (11 mg, 52% for 3 steps); $[\alpha]_D^{27}$ = -298.0 (c 0.1, $CHCl_3$); ^{1}H NMR (400 MHz, $CDCl_3$) δ 8.06 (s, 1H), 7.97 (d, *J* = 9.5 Hz, 1H), 7.56 (d, *J* = 9.6 Hz, 1H), 7.02 (d, *J* = 8.6 Hz, 2H), 6.81 (d, *J* = 8.6 Hz, 2H), 6.55 (d, *J* = 5.9 Hz, 1H), 6.12 (d, *J* = 6.6 Hz, 1H), 5.13 (t, *J* = 9.6 Hz, 1H), 5.07 – 4.99 (m, 1H), 4.30 (dd, J = 5.9, 3.8 Hz, 1H), 4.22 – 4.09 (m, 2H), 3.74 (s, 3H), 3.08 – 2.98 (m, 2H), 2.95 (s, 3H), 2.66 – 2.57 (m, 1H), 2.30 – 2.18 (m, 2H), 1.76 – 1.65 (m, 2H), 1.54 – 1.41 (m, 1H), 1.39 – 1.28 (m, 1H), 1.19 (d, *J* = 7.0 Hz, 3H), 1.06 (d, *J* = 7.0 Hz, 3H), 1.00 (d, *J* = 6.8 Hz, 3H), 0.95 (d, *J* = 6.8 Hz, 3H), 0.92 (t, *J* = 7.3 Hz, 3H), 0.87 (d, *J* = 6.6 Hz, 3H), 0.39 (d, *J* = 6.6 Hz, 3H); ^{13}C NMR (100 MHz, $CDCl_3$) δ 173.0, 172.3, 171.1, 168.9, 168.6, 160.4, 158.8, 150.5, 130.5, 129.2, 122.9, 114.5, 62.4, 58.1, 55.7, 55.4, 51.8, 45.0, 44.5, 33.7, 33.6, 32.4, 30.5, 29.7, 29.7, 20.0, 19.8, 19.2, 18.2, 16.3, 13.8, 13.7; HRESIMS *m/z* 671.3586 $[M+H]^+$ (calcd for $C_{34}H_{51}N_6O_6S^+$, 671.3585).

Synthesis of Analogue 18:

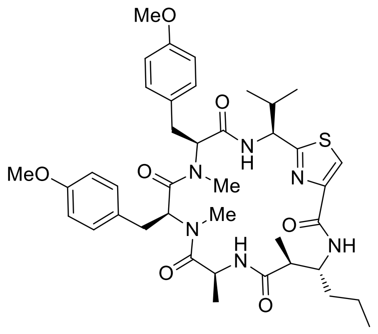

18

Synthesis of analogue **18** was performed in an identical manner as outlined for kakeromamide A (**1**).

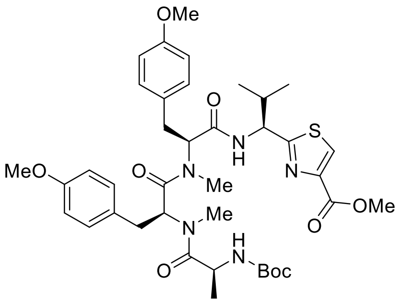

SI-8

The corresponding tetrapeptide **SI-8** was synthesized on a 0.6 mmol scale and obtained as a colorless oil (345 mg, 75% for 2 steps); $[\alpha]_D^{24}$: -160.6 (c 0.65, $CHCl_3$); ^1H NMR (400 MHz, $CDCl_3$) (Mixture of conformers) δ 8.07 (s, 1H), 7.18 – 6.58 (m, 9H), 5.61 – 5.11 (m, 3H), 5.12 – 4.98 (m, 1H), 4.47 – 4.29 (m, 1H), 3.92 (s, 3H), 3.75 – 3.68 (m, 6H), 3.49 (s, 1H), 3.25 – 2.88 (m, 4H), 2.72 (s, 2H), 2.62 – 2.52 (m, 1H), 2.38 – 2.13 (m, 3H), 1.40 (s, 9H), 1.25 – 1.02 (m, 3H), 0.90 – 0.49 (m, 6H); ^{13}C NMR (100 MHz, $CDCl_3$) (Mixture of conformers) δ 175.1, 172.5, 172.1, 171.9, 171.2, 169.6, 168.8, 161.9, 161.8, 158.6, 158.5, 158.3, 155.0, 147.0, 146.9, 130.5, 130.3, 129.8, 129.1, 129.0, 128.5, 128.2, 127.3, 127.2, 114.4, 114.0, 114.0, 113.8, 79.7, 62.4, 57.4, 56.6, 56.4, 56.1, 55.3, 55.1, 55.1, 52.4, 52.1, 46.7, 46.3, 38.6, 34.4, 33.6, 33.0, 32.7, 32.4, 30.7, 30.4, 29.8, 29.5, 28.3, 19.5, 19.3, 18.8, 18.5, 17.5, 17.2; HRESIMS *m/z* 768.3637 $[M+H]^+$ (calcd for $C_{39}H_{54}N_5O_9S^+$,768.3637).

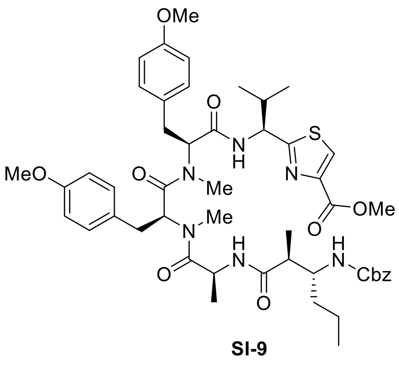

SI-9

The corresponding pentapeptide **SI-9** was synthesized on a 0.4 mmol scale and obtained as a colorless oil (304 mg, 82% for 2 steps); $[\alpha]_D^{25}$: -223.8 (*c* = 0.26, $CHCl_3$); ^1H NMR (300 MHz, $CDCl_3$) (Mixture of conformers) δ 8.10 (s, 1H), 7.44 – 7.28 (m, 5H), 7.21 – 6.89 (m, 4H), 6.88 – 6.46 (m, 7H), 5.58 – 5.21 (m, 2H), 5.18 – 4.91 (m, 4H), 4.83 – 4.48 (m, 1H), 4.00 – 3.84 (m, 3H), 3.81 – 3.65 (m, 6H), 3.56 – 3.39 (m, 2H), 3.27 – 3.08 (m, 1H), 3.06– 2.91 (m, 3H), 2.84 – 2.56 (m, 3H), 2.46 – 2.19 (m, 3H), 1.50 – 1.28 (m, 4H), 1.28 – 1.02 (m, 6H), 0.96 – 0.80 (m, 6H), 0.78 – 0.55 (m, 3H); ^{13}C NMR (75 MHz, $CDCl_3$) (Mixture of conformers) δ 174.7, 174.4, 173.6, 173.2, 172.0, 172.0, 171.9, 171.7, 171.0, 170.8, 169.6, 168.6, 161.8, 158.6, 158.5, 158.4, 158.3, 158.2, 156.7, 156.4, 156.2, 147.0, 146.8, 136.7, 136.6, 130.5, 130.2, 129.8, 129.0, 128.9, 128.9, 128.9, 128.5, 128.4, 128.4, 128.1, 128.1, 128.0, 127.9, 127.8, 127.8, 127.5, 127.2, 114.4, 114.1, 114.0, 113.8, 66.6, 66.5, 62.5, 62.2, 57.3, 56.4, 56.4, 56.0, 55.3, 55.3, 55.2, 55.1, 53.6, 52.5, 52.4, 52.0, 45.5, 45.4, 45.2, 45.1, 43.5, 36.5, 36.3, 34.7, 34.3, 34.2, 33.8, 33.6, 33.1, 32.9, 32.8, 32.5, 30.8, 30.5, 30.3, 29.8, 29.8, 29.7, 29.4, 29.3, 19.6, 19.5, 19.4, 19.3, 18.8, 18.2, 18.0, 17.4, 17.2, 17.0, 16.0, 15.7, 13.9, 13.9, 13.8; HRESIMS *m/z* 929.4477 $[M+H]^+$ (calcd for $C_{49}H_{65}N_6O_{10}S^+$, 929.4477).

Analogue **18** was synthesized on a 0.03 mmol scale and obtained as an amorphous solid (6 mg, 27% for 3 steps); $[\alpha]_D^{27}$ = -298.0 (c 0.1, $CHCl_3$); ^1H NMR (400 MHz, $CDCl_3$) δ 8.05 (s, 1H), 7.57 (d, *J* = 9.5 Hz, 1H), 7.43 (d, *J* = 10.6 Hz, 1H), 7.00 (d, *J* = 8.6 Hz, 2H), 6.82 – 6.71 (m, 4H), 6.69 (d, *J* = 6.2 Hz, 1H), 6.65 (d, *J* = 8.6 Hz, 2H), 5.34 (dd, *J* = 11.2, 4.9 Hz, 1H), 5.16 (dd, *J* = 10.6, 4.7 Hz, 1H), 5.11 (t, *J* = 9.5 Hz, 1H), 4.72 – 4.61 (m, 1H), 4.23 – 4.12 (m, 1H), 3.75 (s, 3H), 3.43 (s, 3H), 3.01 (s, 3H), 2.97 (s, 3H), 3.01 – 2.92 (m, 1H), 2.80 (dd, *J* = 14.5, 10.4 Hz, 1H), 2.73 (dd, *J* = 15.9, 11.2 Hz, 1H), 2.59 – 2.45 (m, 1H), 2.31 – 2.15 (m, 1H), 1.80 – 1.67 (m, 1H), 1.49 – 1.45 (m, 1H), 1.43 (d, *J* = 6.6 Hz, 3H), 1.37 – 1.27 (m, 2H), 1.18 (d, *J* = 7.0 Hz, 3H), 1.13 – 1.00 (m, 1H), 0.96 (d, *J* = 6.7 Hz, 3H), 0.91 (t, *J* = 7.3 Hz, 3H), 0.86 (d, *J* = 6.6 Hz, 3H); ^{13}C NMR (100 MHz, $CDCl_3$) δ 174.1, 171.2, 170.9, 168.7, 168.2, 160.0, 158.6, 158.4, 150.6, 130.2, 129.0, 128.9, 127.7, 122.9, 114.6, 114.1, 62.4, 55.2, 55.2, 55.1, 51.2, 50.9, 46.5, 45.5, 34.1, 33.7, 32.9, 30.9, 30.5, 29.2, 19.9, 19.8, 19.0, 18.9, 13.8, 13.7; HRESIMS *m/z* 763.3848 $[M+H]^+$ (calcd for $C_{40}H_{55}N_6O_7S^+$, 763.3847).

Synthesis of Analogue 19:

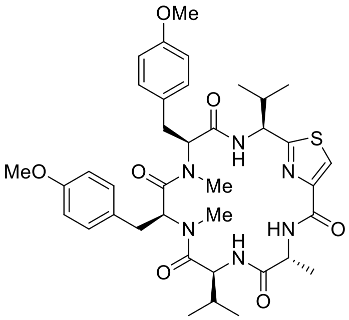

19

Synthesis of analogue **19** was performed in an identical manner as outlined for kakeromamide A (**1**).

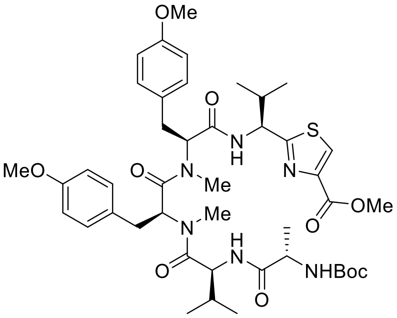

SI-10

The corresponding pentapeptide **SI-10** was synthesized on a 0.4 mmol scale and obtained as a colorless oil (302 mg, 87% for 2 steps); $[\boldsymbol{\alpha}]_{\mathbf{D}}^{\mathbf{25}}$: -219 (*c* = 0.26, $CHCl_3$); ^{1}H NMR (500 MHz, DMSO-*d6*) (Mixture of conformers) *δ* 8.58 (d, *J* = 8.5 Hz, 1H), 8.46 (s, 1H), 7.46 (d, *J* = 9.1 Hz, 1H), 7.17 (d, *J* = 8.5 Hz, 2H), 7.04 (d, *J* = 8.3 Hz, 2H), 7.00 (d, *J* = 7.7 Hz, 1H), 6.85 (d, *J* = 8.3 Hz, 2H), 6.72 (d, *J* = 8.5 Hz, 2H), 5.53 (dd, *J* = 12.1, 4.8 Hz, 1H), 5.47 – 5.34 (m, 1H), 5.02 – 4.85 (m, 1H), 4.33 (dd, *J* = 9.1, 5.7 Hz, 1H), 4.01 – 3.89 (m, 1H), 3.84 (s, 3H), 3.75 (s, 3H), 3.71 (dd, *J* = 4.4, 2.6 Hz, 1H), 3.68 (s, 3H), 3.13 (dd, *J* = 14.9, 4.6 Hz, 1H), 3.05 (dd, *J* = 13.8, 7.7 Hz, 1H), 2.80 (d, *J* = 2.6 Hz, 1H), 2.77 (s, 3H), 2.48 (dd, *J* = 13.8, 6.3 Hz, 1H), 2.34 – 2.29 (m, 1H), 2.27 (s, 2H), 1.70 – 1.55 (m, 1H), 1.37 (s, 9H), 1.11 (d, *J* = 7.1 Hz, 3H), 0.88 (d, *J* = 6.8 Hz, 3H), 0.84 (d, *J* = 6.7 Hz, 3H), 0.75 (d, *J* = 6.5 Hz, 3H), 0.71 (d, *J* = 6.7 Hz, 3H); ^{13}C NMR (125 MHz, DMSO-*d6*) (Mixture of conformers) *δ* 172.9, 172.3, 170.4, 170.2, 169.9, 161.1, 158.0, 157.7, 157.6, 155.0, 145.4, 129.9, 129.5, 129.4, 129.0, 128.7, 113.6, 113.4, 79.1, 78.0, 56.4, 56.3, 54.9, 54.7, 54.6, 52.9, 51.8, 49.6, 39.5, 33.5, 33.3, 31.9, 30.4, 29.8, 29.3, 28.0, 19.6, 19.2, 18.0, 17.6, 16.7; HRESIMS *m/z* 867.4309 $[M+H]^+$ (calcd for $C_{44}H_{63}N_6O_{10}S^+$, 867.4321).

Analogue **19** was synthesized on a 0.03 mmol scale and obtained as an amorphous solid (5 mg, 24% for 3 steps); $[\alpha]_{\mathrm{D}}^{27}$ = -50.5 (c 0.21, $CHCl_3$); ^{1}H NMR (400 MHz, $CDCl_3$) *δ* 8.37 (d, *J* = 9.5 Hz, 1H), 8.00 (s, 1H), 7.74 (d, *J* = 7.5 Hz, 1H), 7.31 (d, *J* = 10.7 Hz, 1H), 6.94 (d, *J* = 8.6 Hz, 2H), 6.73 – 6.62 (m, 4H), 6.47 (d, *J* = 8.6 Hz, 2H), 5.16 – 5.04 (m, 2H), 4.80 – 4.64 (m, 3H), 3.75 (s, 3H), 3.44 (s, 3H), 3.13 (dd, *J* = 14.8, 4.1 Hz, 1H), 2.92 (s, 3H), 2.92 (s, 3H), 2.80 (dd, *J* = 14.6, 10.6 Hz, 1H), 2.56 (dd, *J* = 17.0, 12.2 Hz, 1H), 2.49

– 2.34 (m, 1H), 2.19 – 2.09 (m, 1H), 1.58 (d, J = 7.4 Hz, 3H), 1.22 – 1.20 (m, 1H), 1.04 (d, J = 6.7 Hz, 3H), 0.90 (d, J = 6.8 Hz, 3H), 0.89 (d, J = 6.8 Hz, 3H), 0.79 (d, J = 6.7 Hz, 3H); ^{13}C NMR (100 MHz, $CDCl_3$) δ 171.9, 171.2, 171.1, 168.3, 167.5, 160.1, 157.5, 157.3, 149.8, 129.1, 128.4, 127.3, 127.2, 121.7, 113.6, 113.2, 76.2, 62.5, 55.0, 54.3, 54.0, 52.7, 51.2, 48.8, 32.7, 32.1, 30.6, 30.3, 29.6, 28.7, 19.1, 18.8, 18.1, 15.2; HRESIMS *m/z* 735.3541 $[M+H]^+$ (calcd for $C_{38}H_{51}N_6O_7S^+$, 735.3534).

V. Procedures for Biological Tests:

Induction of neural stem cells in vitro: Neural stem cells (NSCs) were obtained following the procedure (Iwata, et al., 2016; Nakayama, et al., 2006) with modification. Embryoid bodies (EBs) were induced by hanging drop method for 72 hours using 7,500 cells per 20 µL of ESCs culturing medium without LIF. Then the EBs were cultured in non-adhesive bacterial dishes in Neuron Culture Medium (Sumitomo Bakelite, Tokyo, Japan) supplemented with 20 ng/mL rhEGF (R&D Systems, Minneapolis, MN, USA) and 20 ng/mL rhFGF-2 (R&D Systems). After this step, these EBs were transferred to matrigel (BD biosciences, Franklin Lakes, NJ, USA)-coated dishes in neurobasal medium (Gibco) with 2% B-27 supplement (Gibco), 1% P/S, 20 ng/mL rhEGF, and 20 ng/mL rhFGF-2 for 16 days. Then the NSCs, which is migrated from outside of the EBs, were collected and cryopreserved until being used in the neural differentiation assay.

NSC differentiation assay: The cryopreserved NSCs were seeded on a Matrigel-coated 96 well plate at 10,000 cells per well in neurobasal medium supplemented with 2% B-27 supplement, 1% P/S, 20 ng/mL rhEGF, and 20 ng/mL rhFGF-2, and cultured for 72 hours. After this step, the cells were cultured with the compound in differentiation medium (1:1 mixture of DMEM and Ham's F-12 (Wako) supplemented with 2% B-27, 1% P/S, and 1% FBS) for 72 hours.

Cell staining: To label neurons, we used NeuO, (Er et al., 2015) a fluorescent probe which could selectively stain living neurons. The cells were incubated with 2 µM of NeuO for 1 hour in Opti-MEM I (1X) + GlutaMAX Reduced Serum Medium (Gibco), and their nuclei were counterstained with Hoechst 33342 (1:1000, DOJINDO, Kumamoto, Japan). After washing in differentiation medium for 30 min to reduce non-specific binding of NeuO, cell images were obtained with IX71 microscope (Olympus, Tokyo, Japan) at 4x magnification to calculate a ratio of neurons to the whole cells.

Immunofluorescence: The cells were fixed in 4% paraformaldehyde (Wako) at 4°C for 30 min, and blocked in phosphate-buffered saline (PBS, Wako) with 5% skim milk (Wako) and 0.2% TritonX-100 (Alfa Aesar, Thermo Fisher Scientific, Inc., Waltham, MA, USA) at 4°C for 30 min. The fixed cells were treated with

anti-Glial Fibrillary Acidic Protein (GFAP) antibody (1:500, Merck Millipore) at 4°C overnight. Then, the cells were treated with chromeo 488 conjugated goat anti-mouse IgG (1:200, Active motif, Carlsbad, CA, USA) at room temperature for 2 hours, counterstain of the nuclei with Hoechst 33342 (1:1000). Fluorescent images were obtained with IX71 microscope at 4x magnification to calculate a ratio of GFAP-positive cells to the whole cells.

VI. Comparison of NMR Data and Spectra of Natural and Synthetic Kakeromamide A

Comparison of ^{1}H NMR Spectra of Natural and Synthetic Kakeromamide A

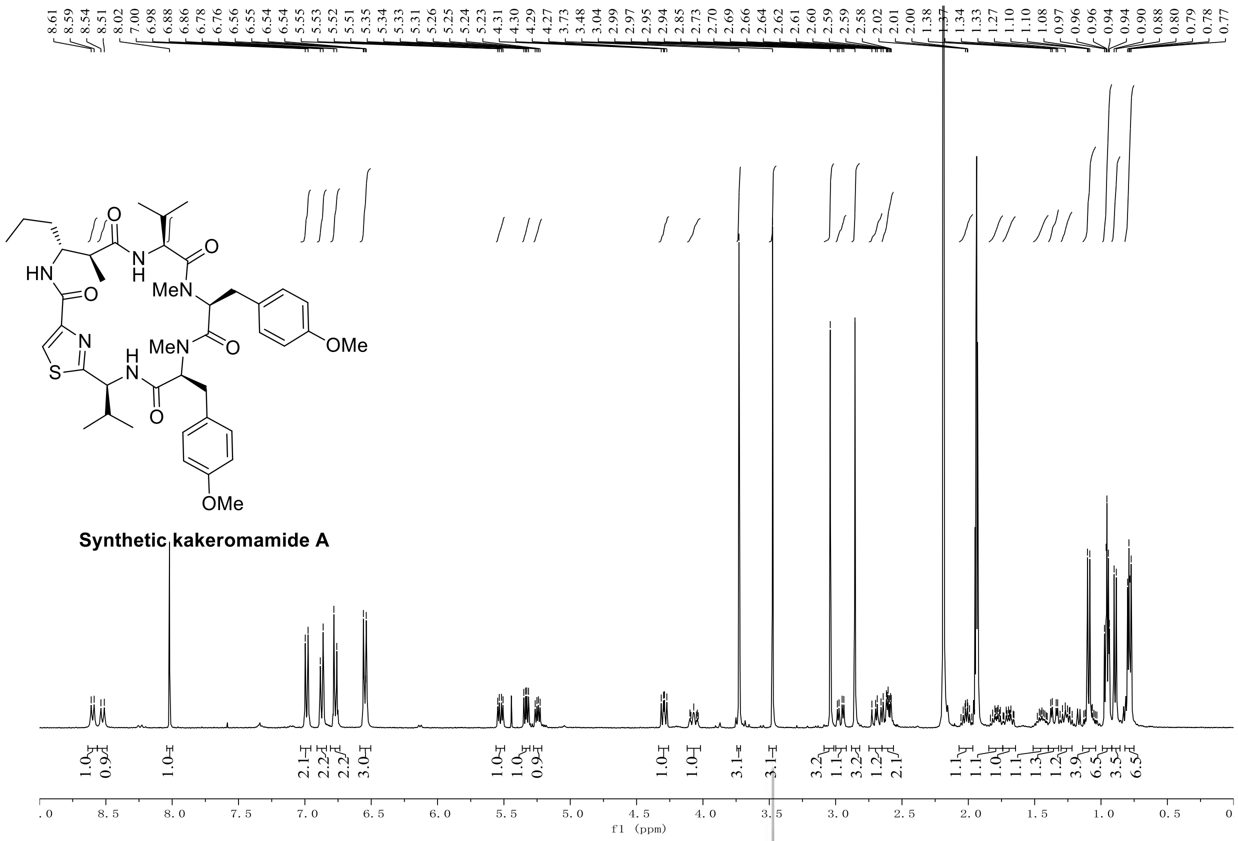

Synthetic kakeromamide A

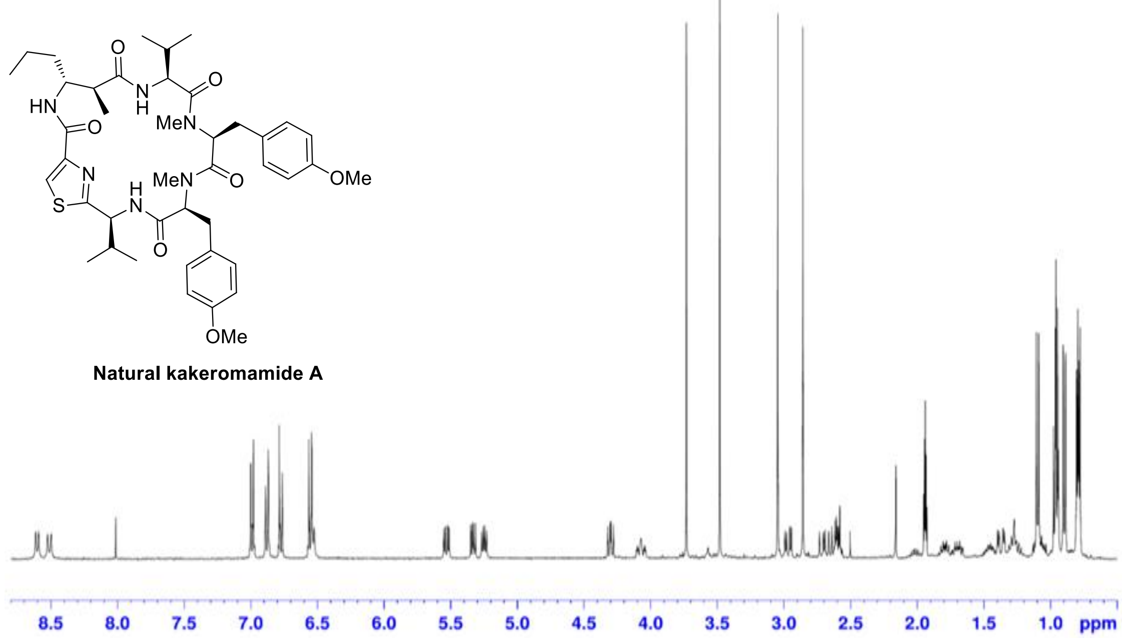

Natural kakeromamide A

Comparison of ^{13}C NMR Spectra of Natural and Synthetic Kakeromamide

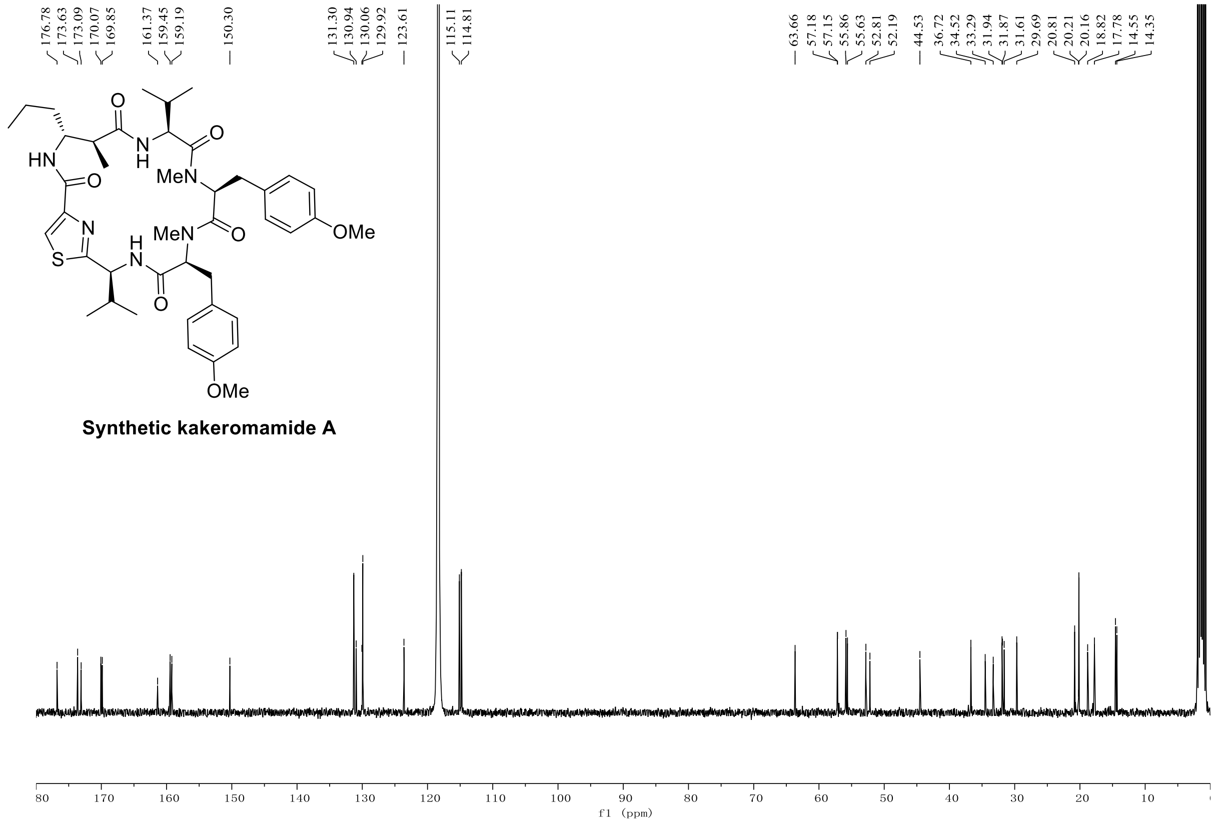

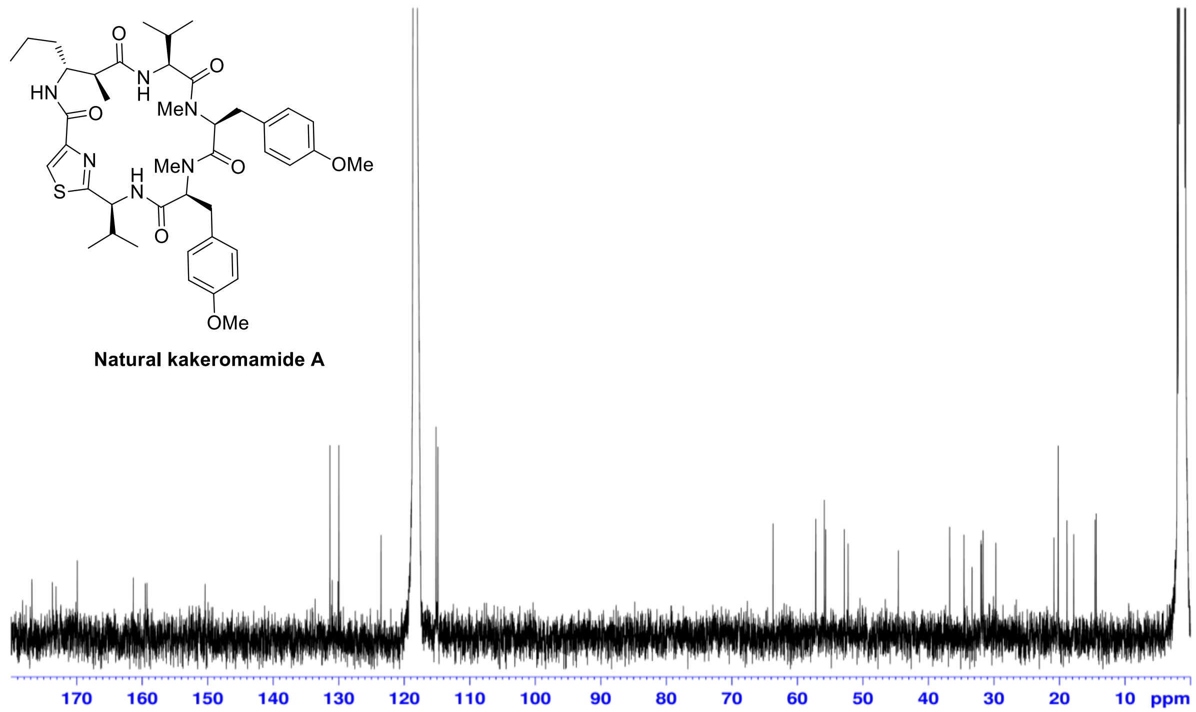

VI. NMR Spectra for Compounds 6, 10, 5, 4, 20, 3, 14, 15, 2, SI-1, SI-2, SI-3, SI-4, 16, SI-5, SI-6, SI-7, 17, SI-8, SI-9, 18, SI-10, 19

^{1}H NMR and ^{13}C NMR Spectra of Compound 6

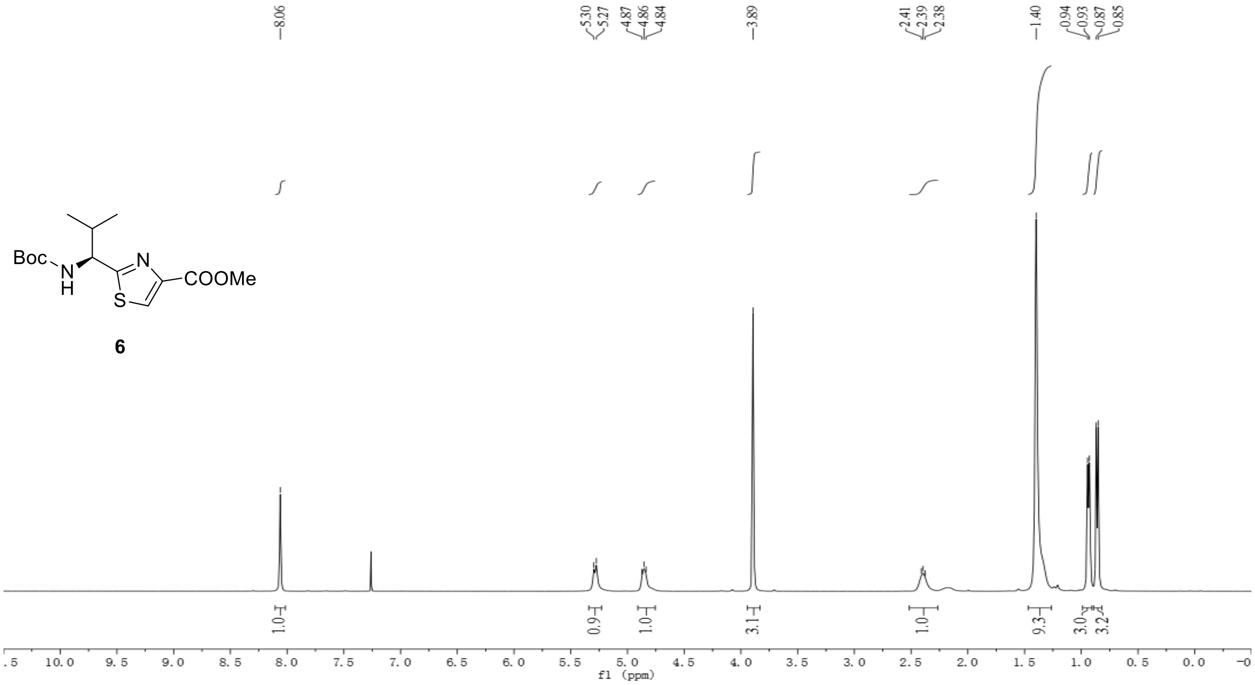

6

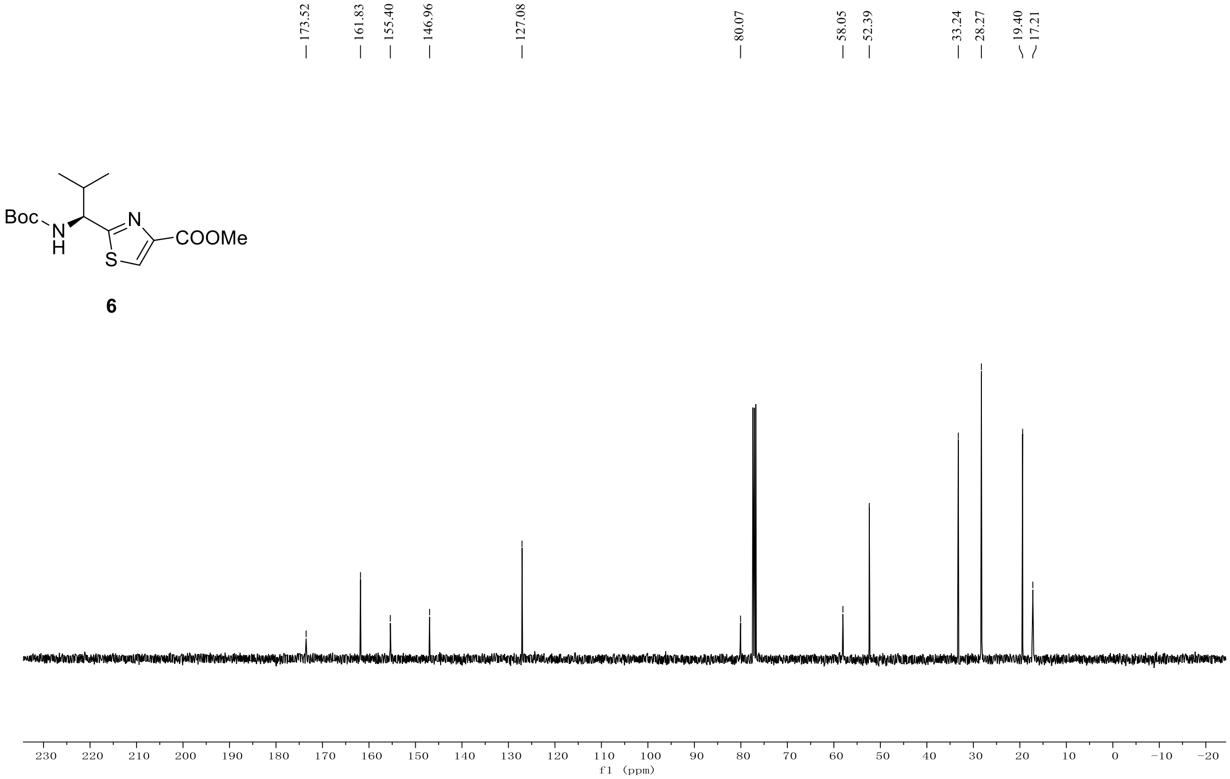

6

^{1}H NMR and ^{13}C NMR Spectra of Compound 10

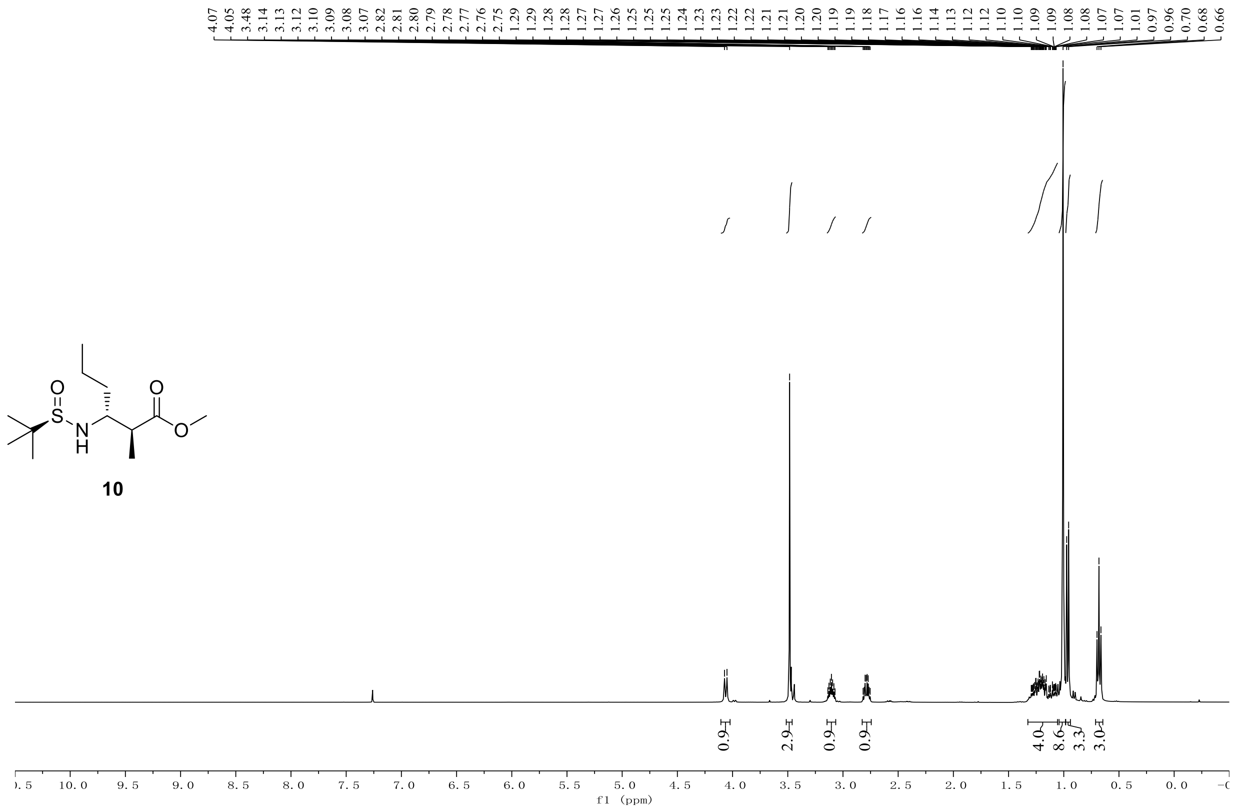

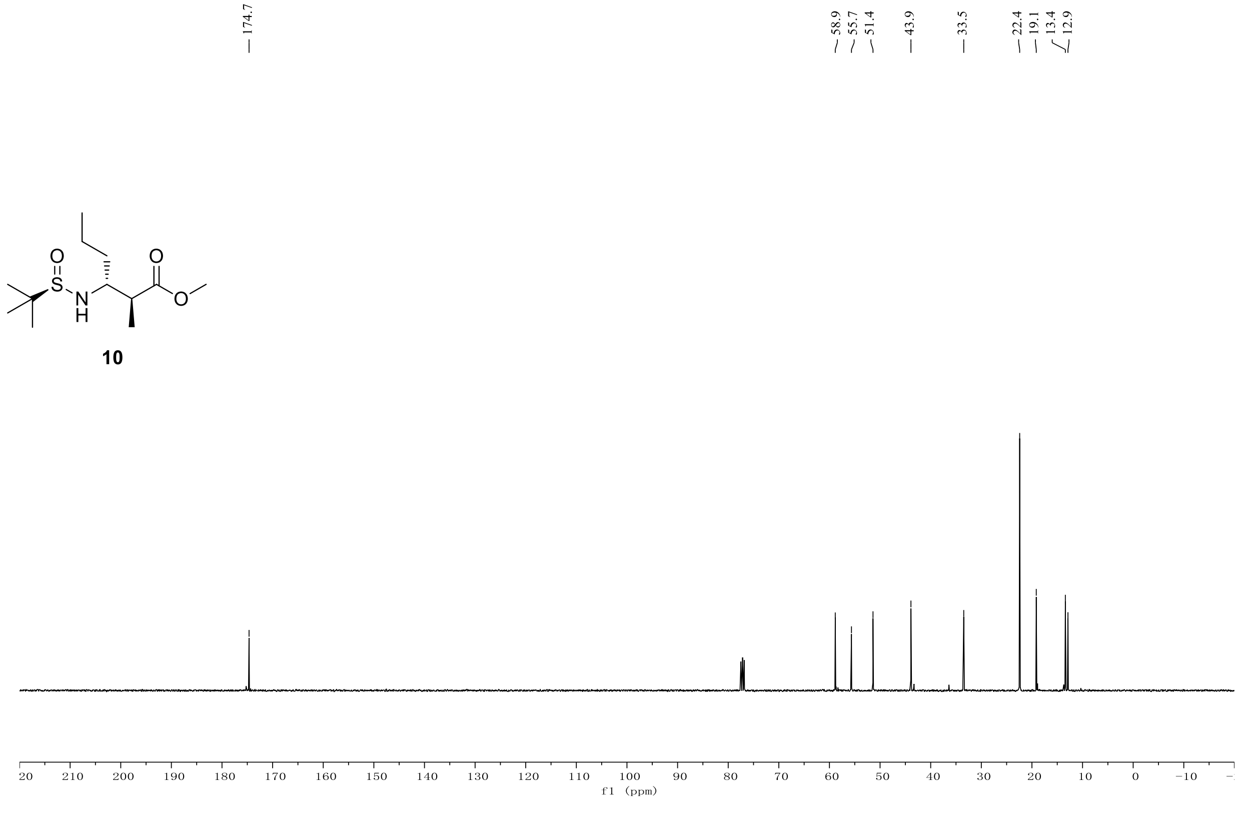

^{1}H NMR and ^{13}C NMR Spectra of Compound 5

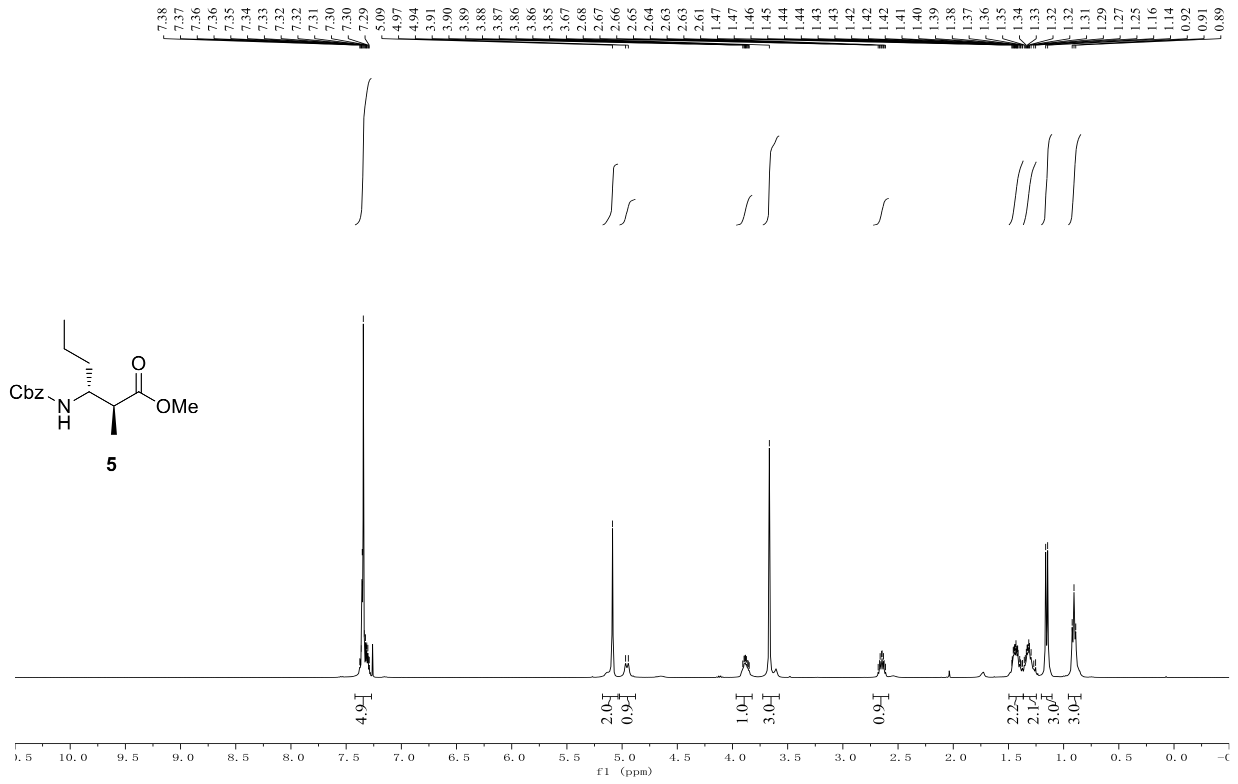

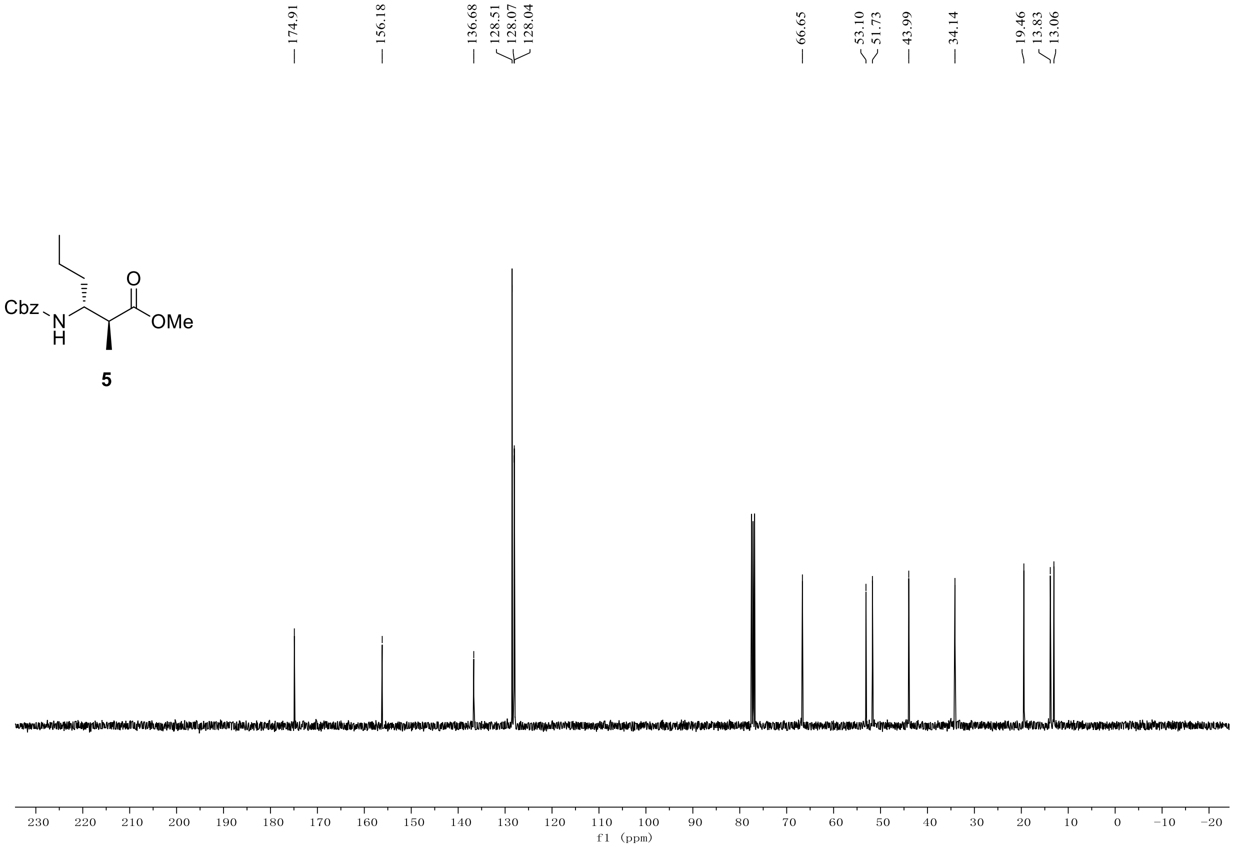

^{1}H NMR and ^{13}C NMR Spectra of Compound 4

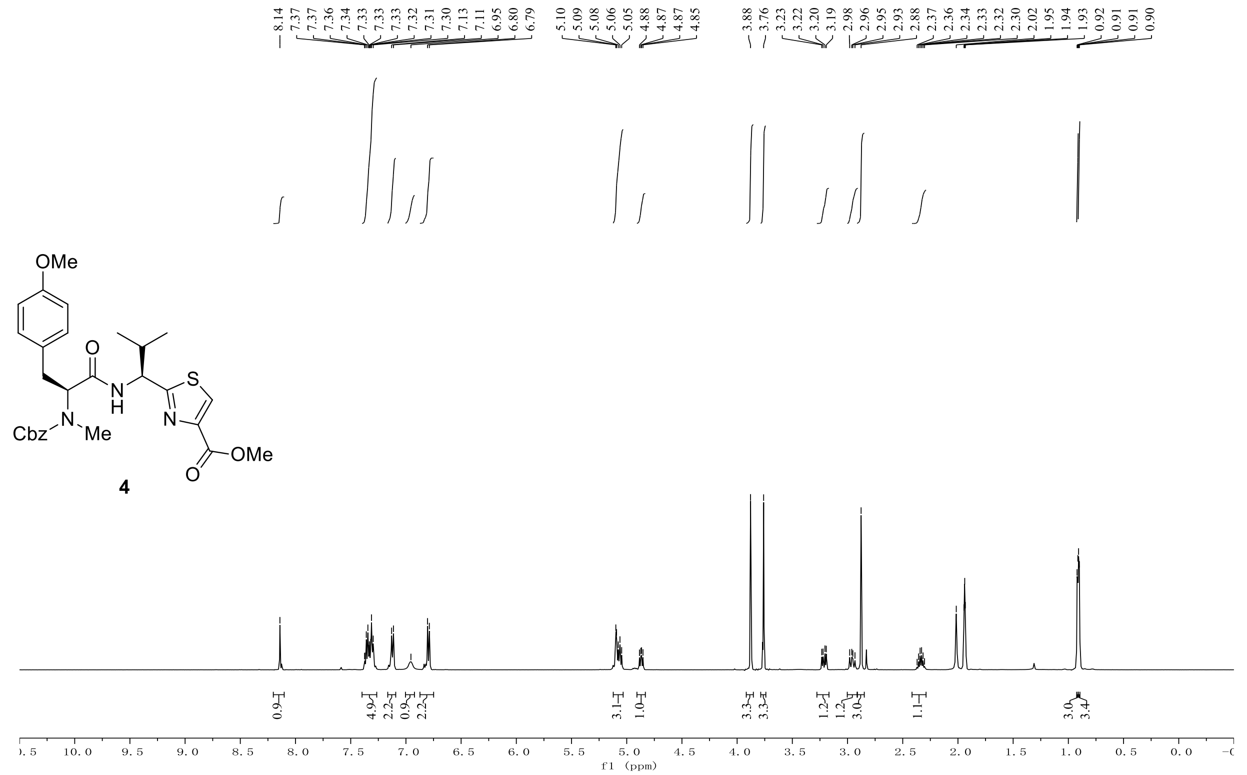

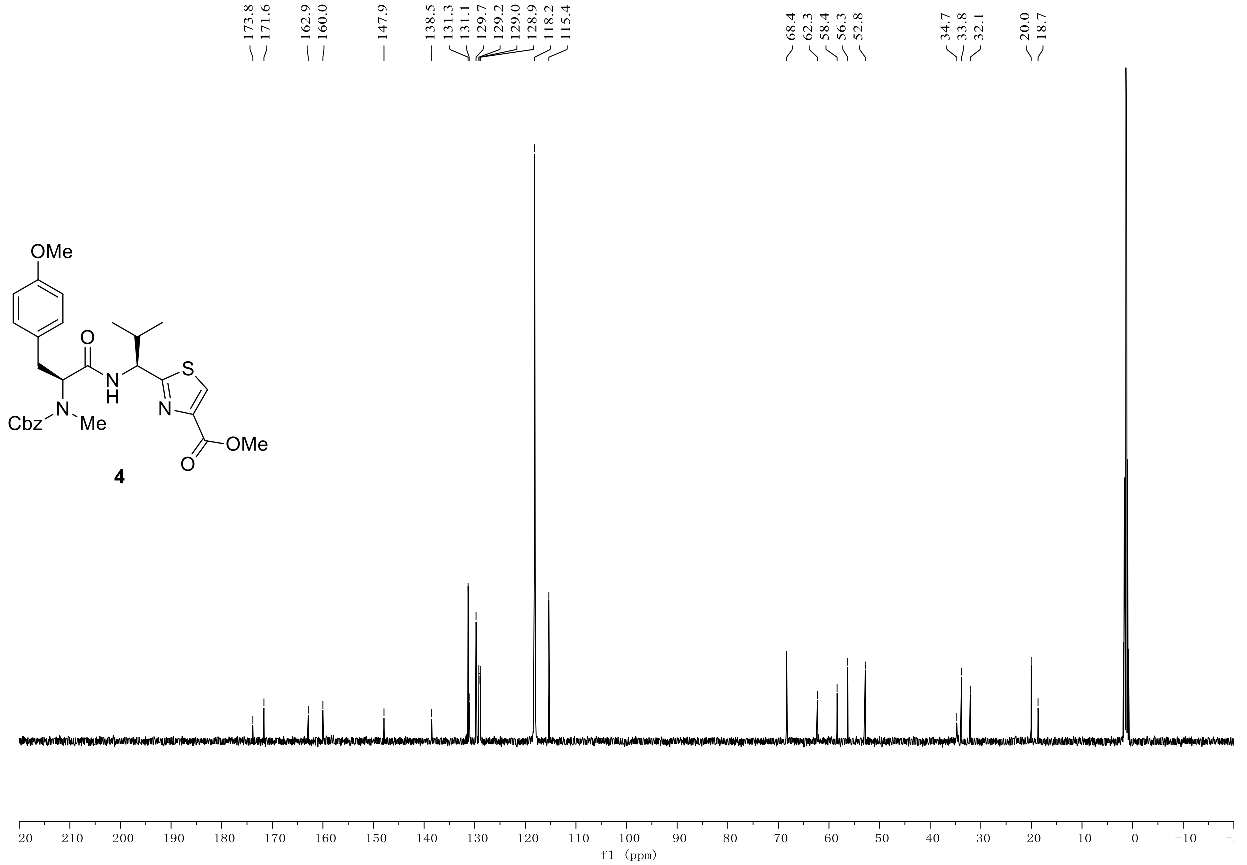

^{1}H NMR Spectrum of Compound 20

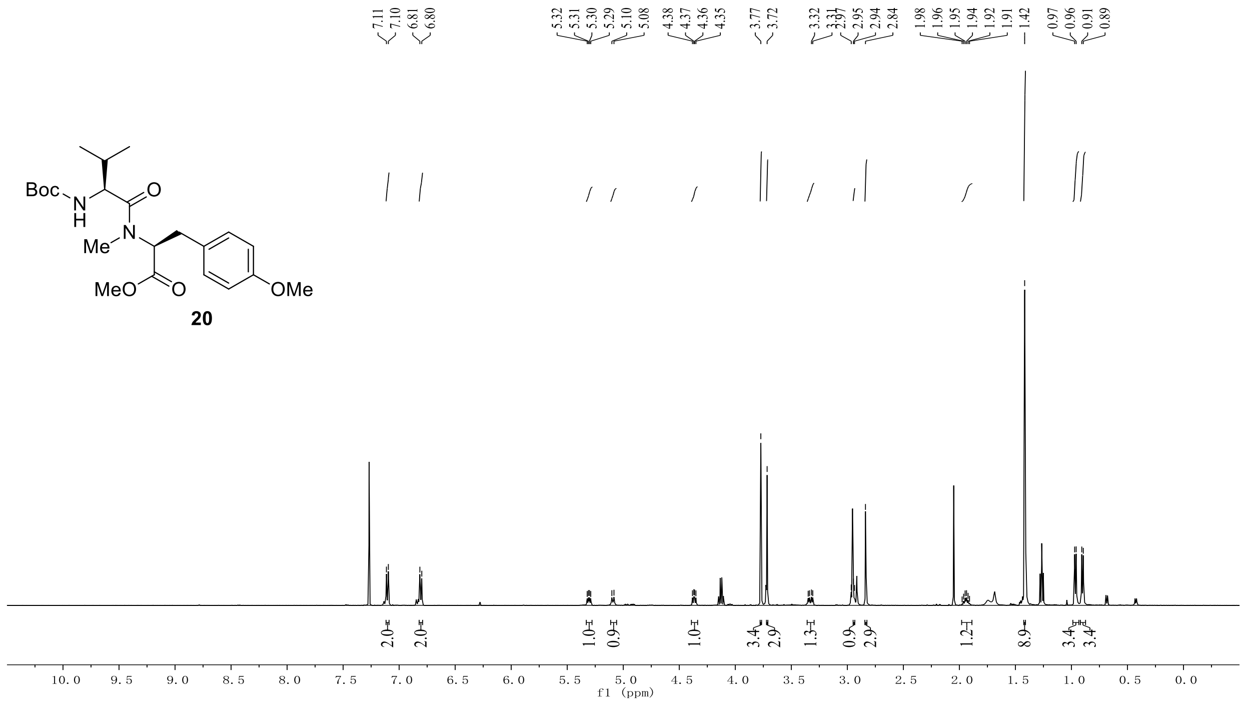

^{1}H NMR Spectrum of Compound 3

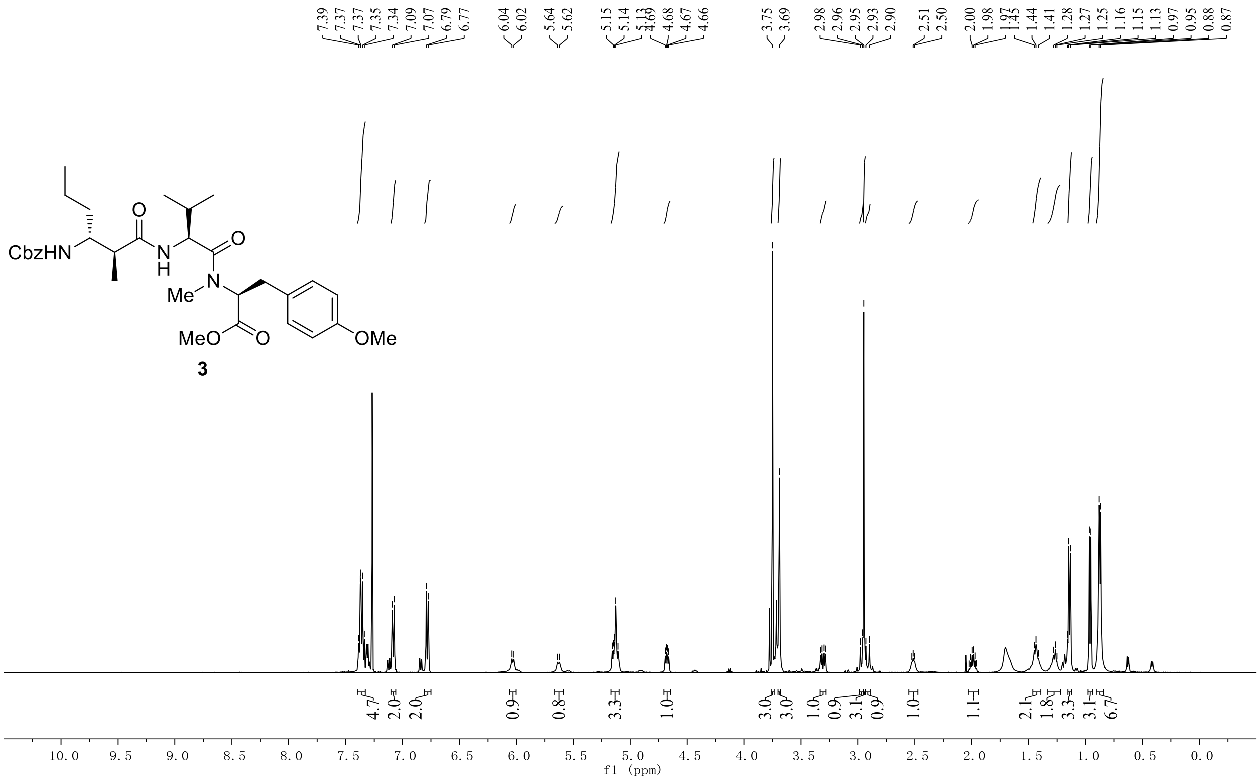

^{1}H NMR and ^{13}C NMR Spectra of Compound 14

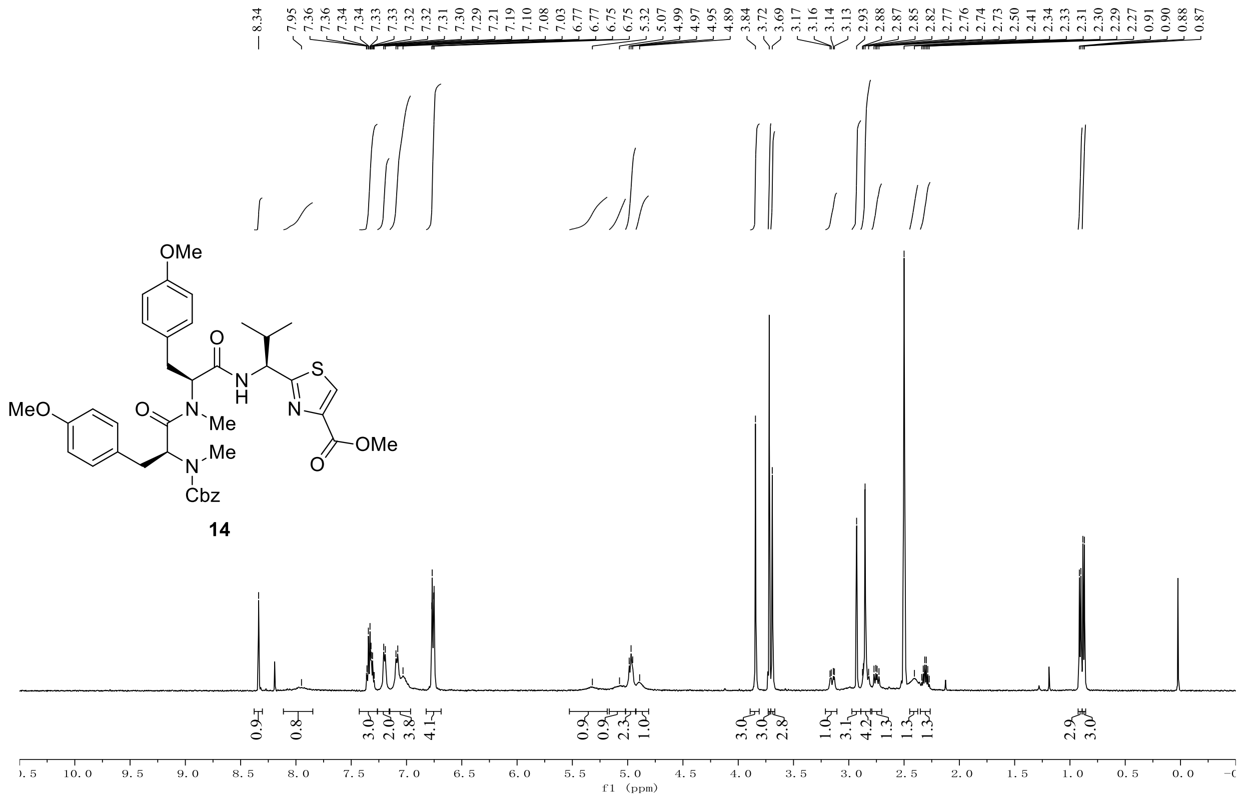

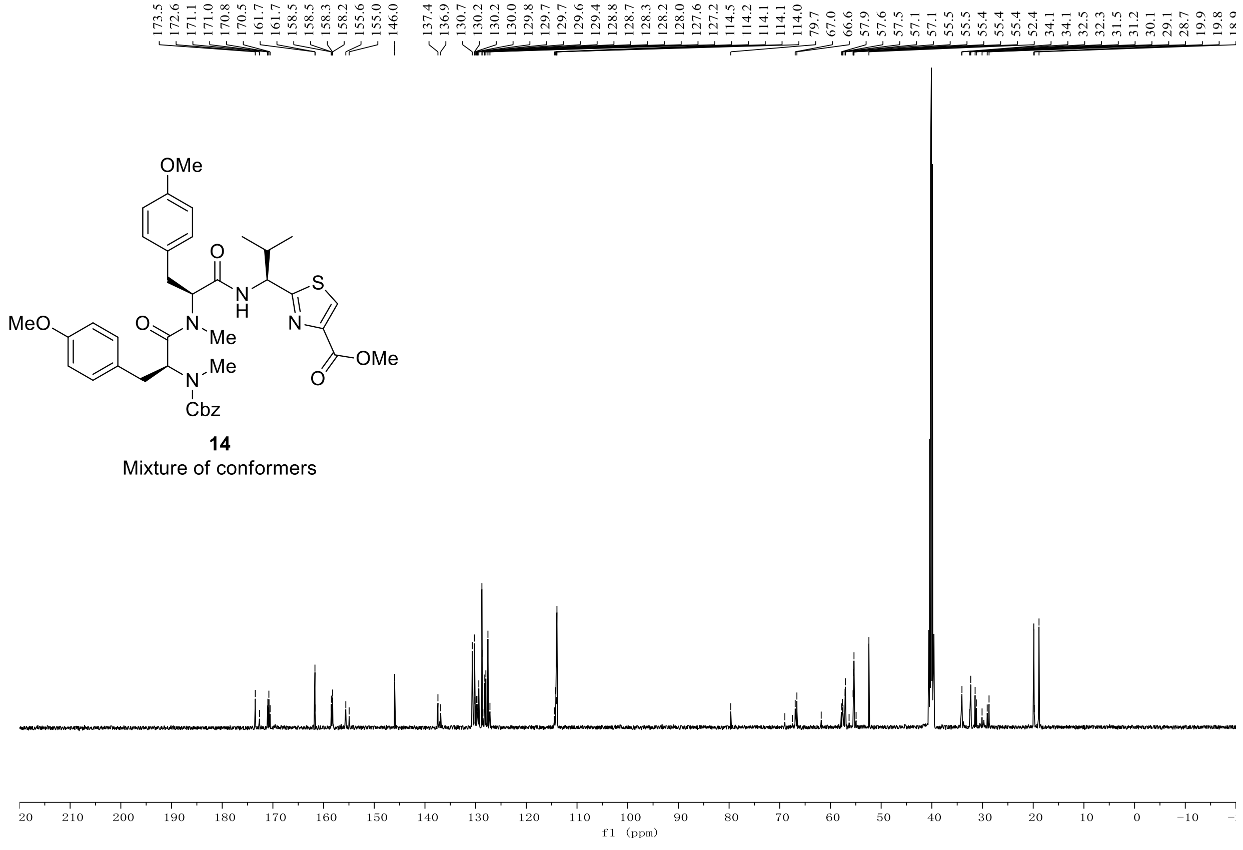

^{1}H NMR and ^{13}C NMR Spectra of Compound 15

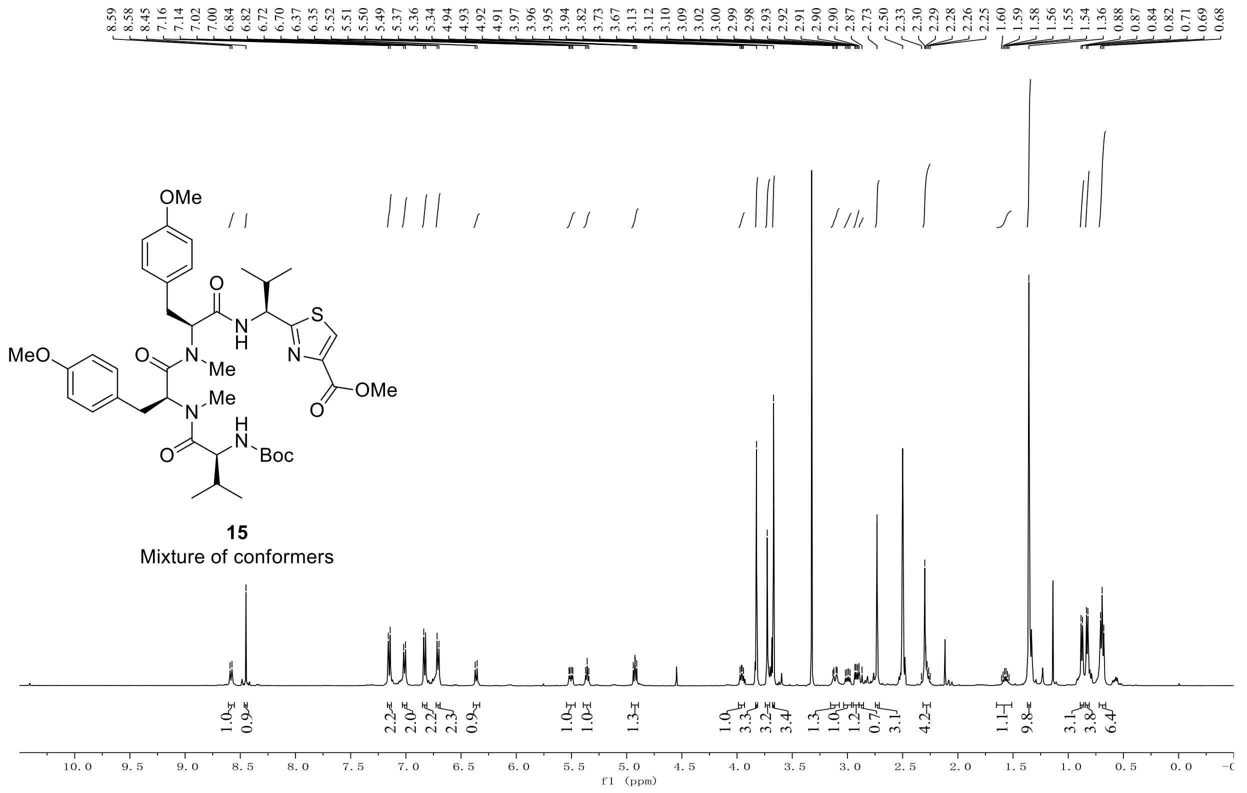

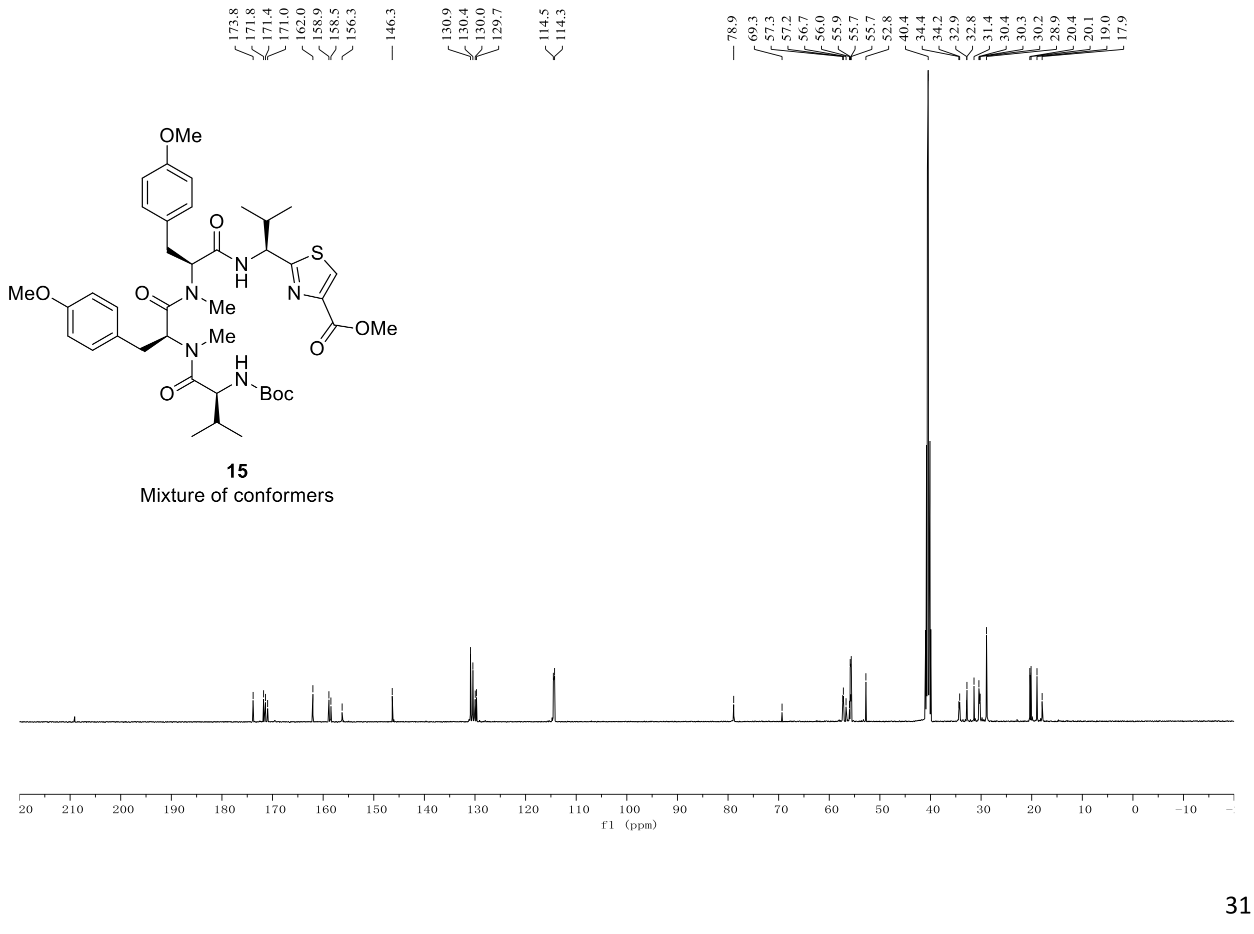

^{1}H NMR and ^{13}C NMR Spectra of Compound 2

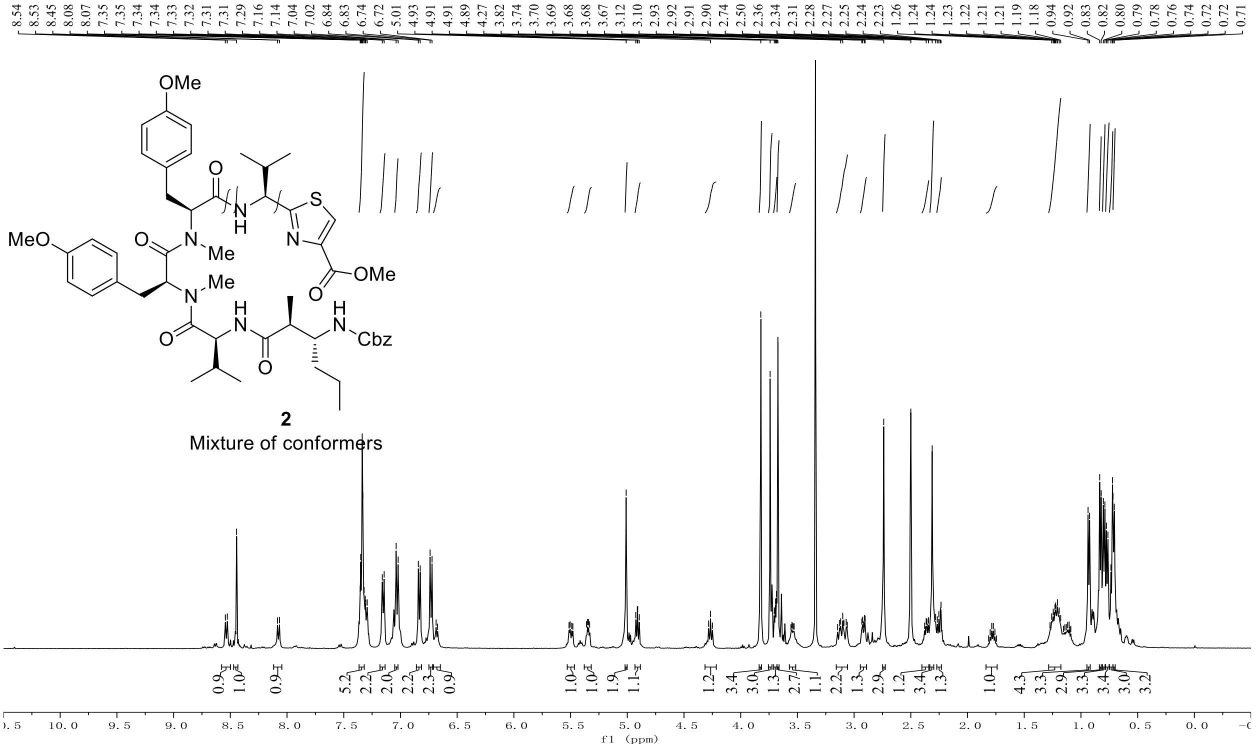

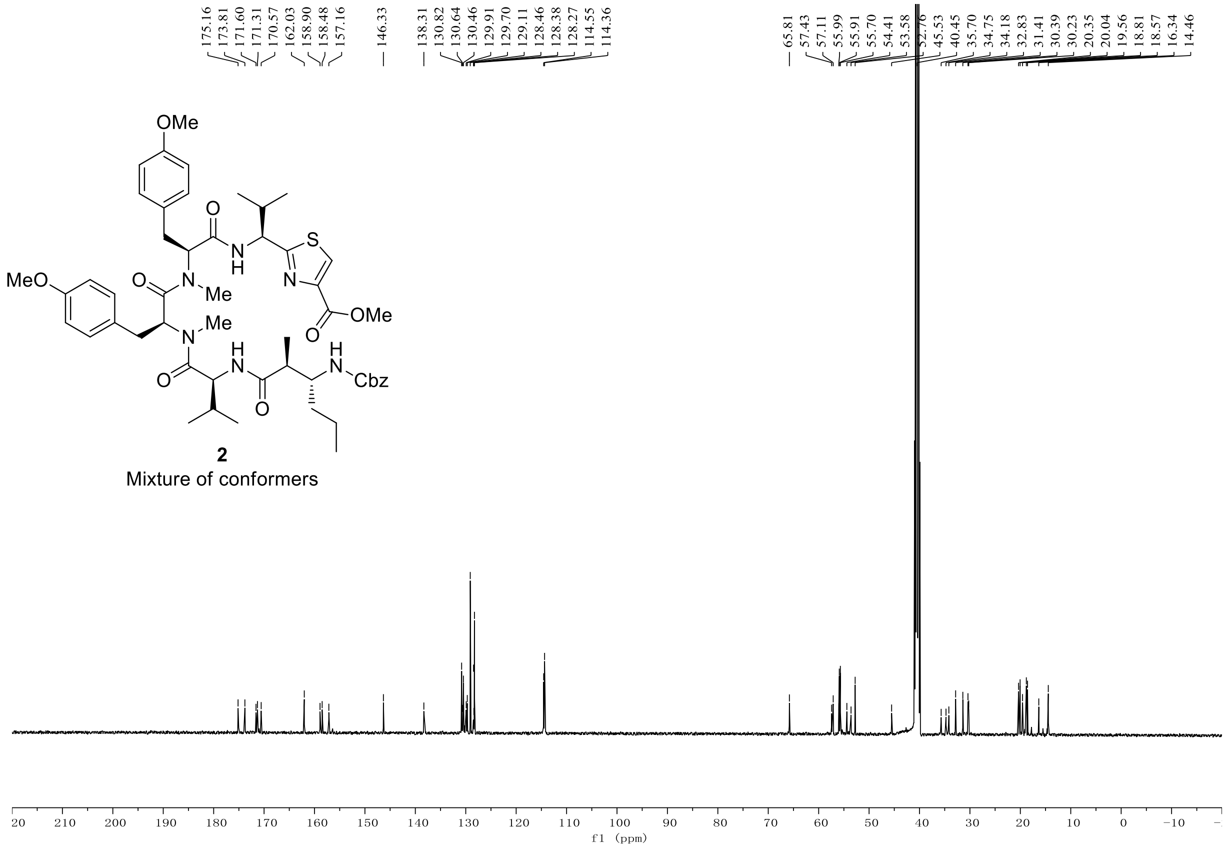

^{1}H NMR and ^{13}C NMR Spectra of Compound SI-1

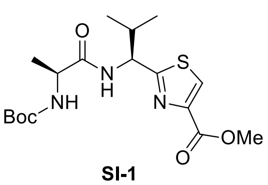

SI-1

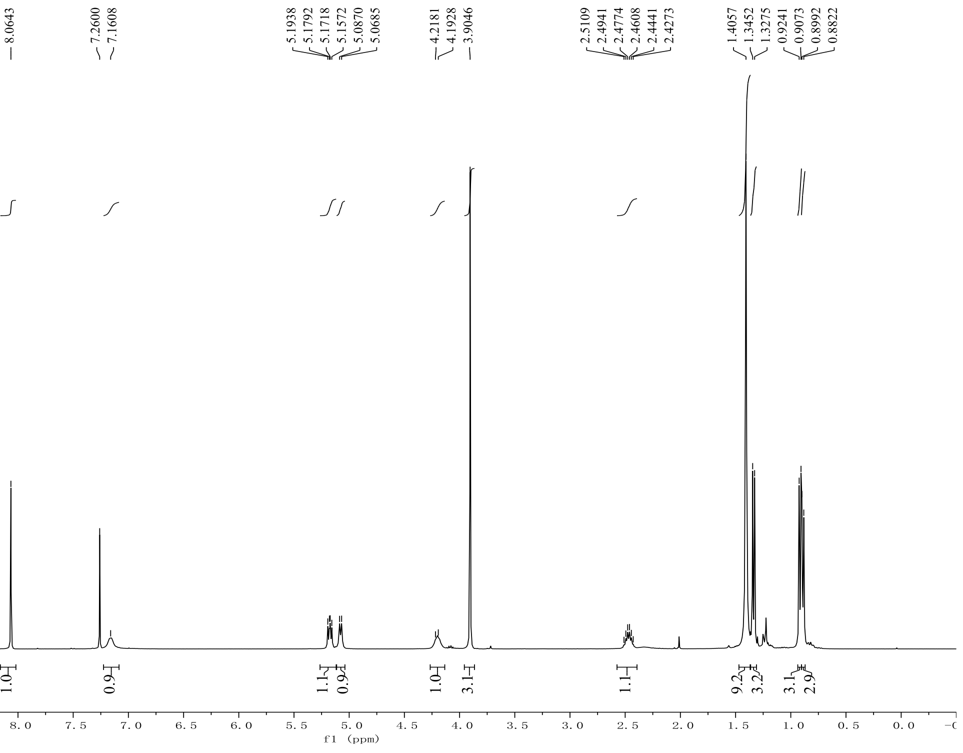

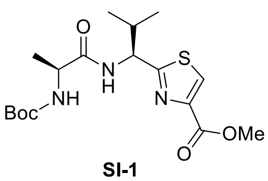

SI-1

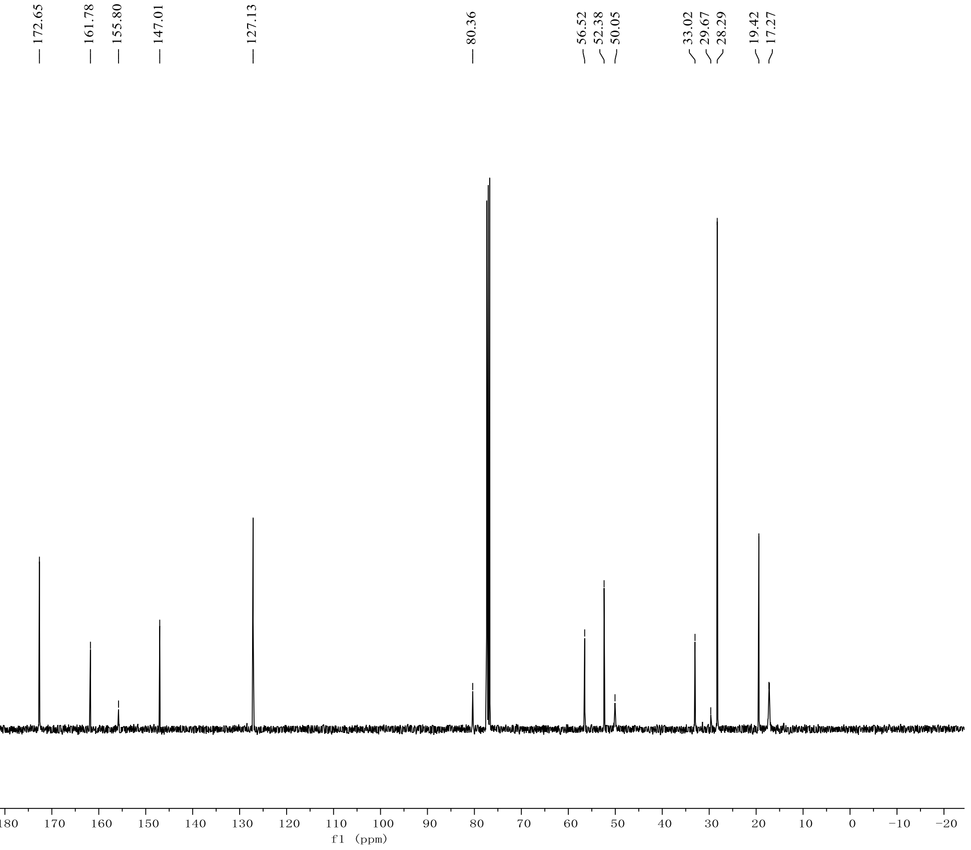

^{1}H NMR and ^{13}C NMR Spectra of Compound SI-2

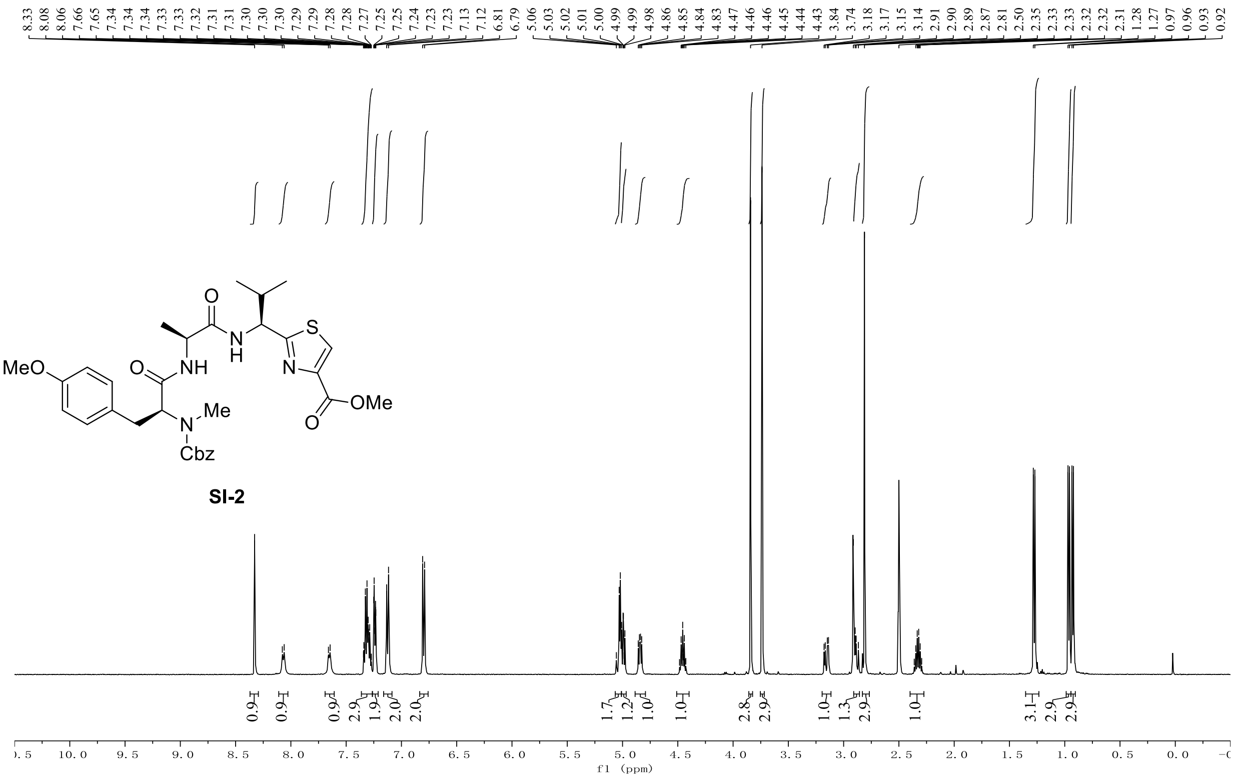

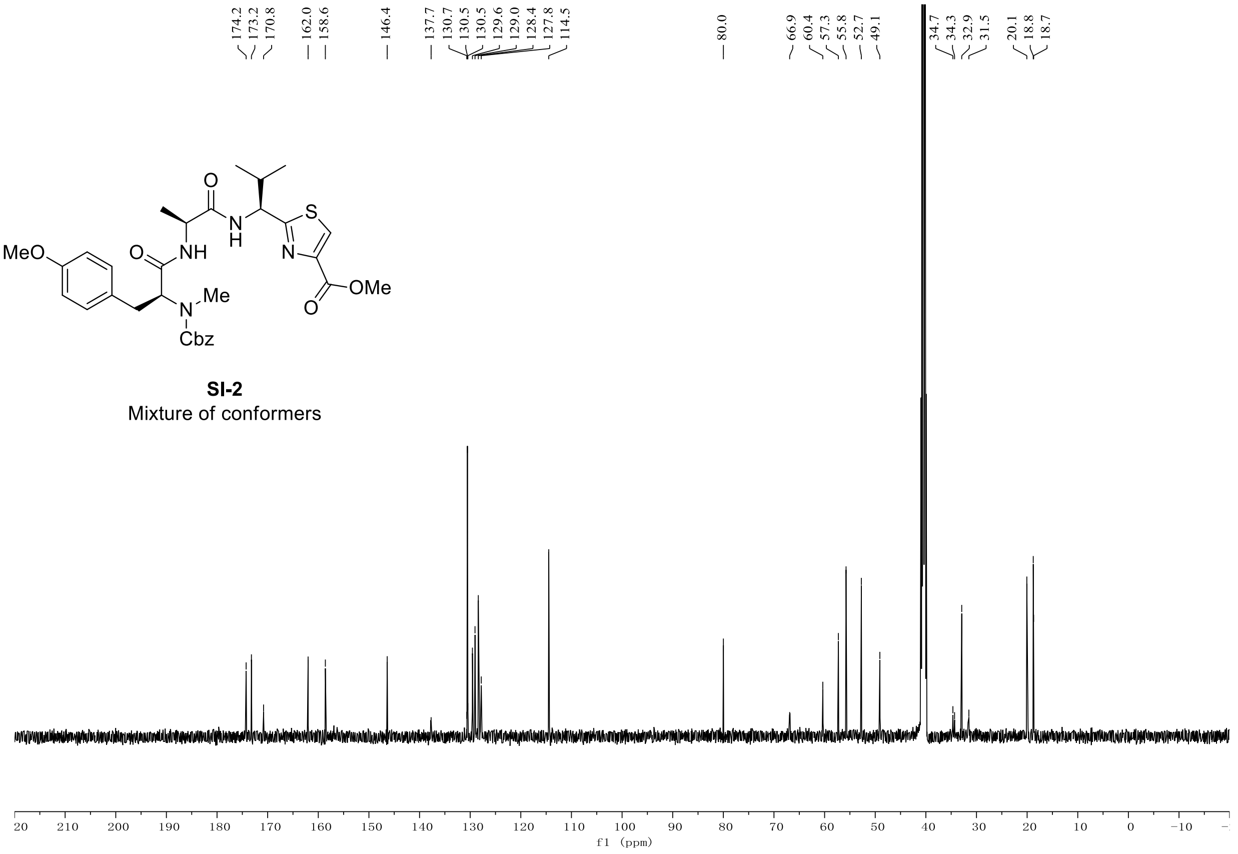

^{1}H NMR and ^{13}C NMR Spectra of Compound SI-3

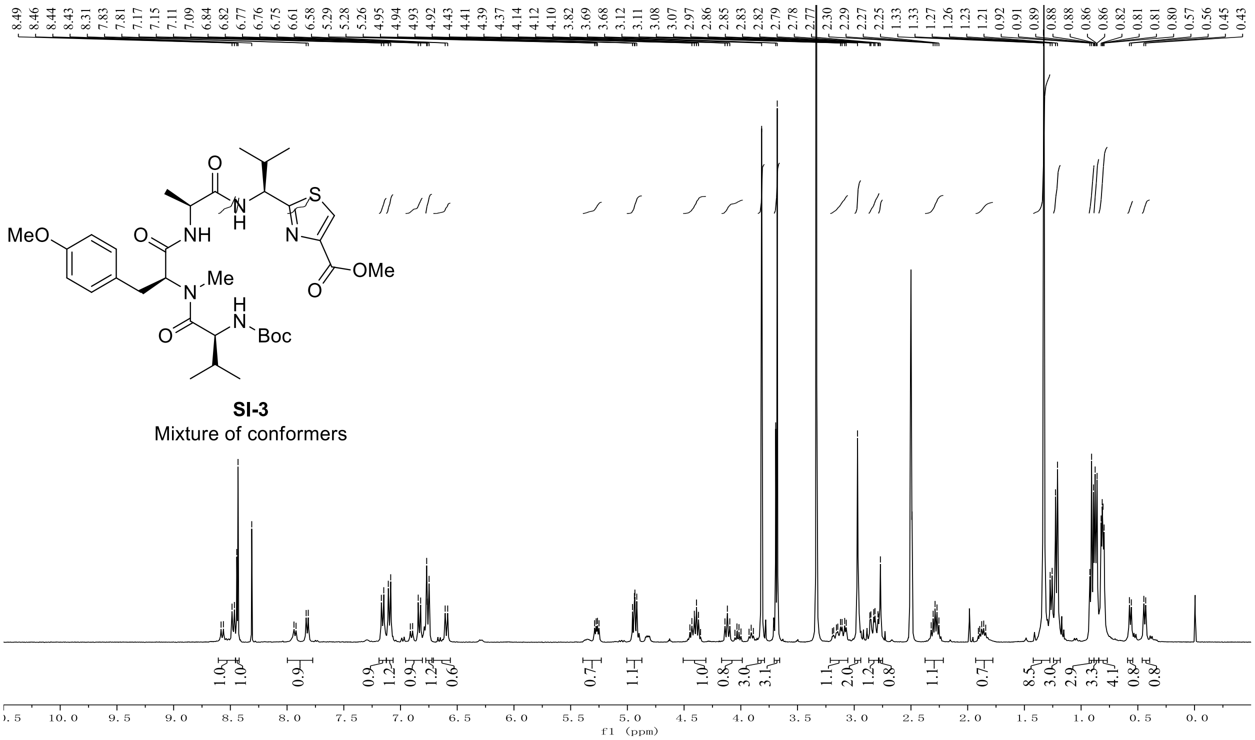

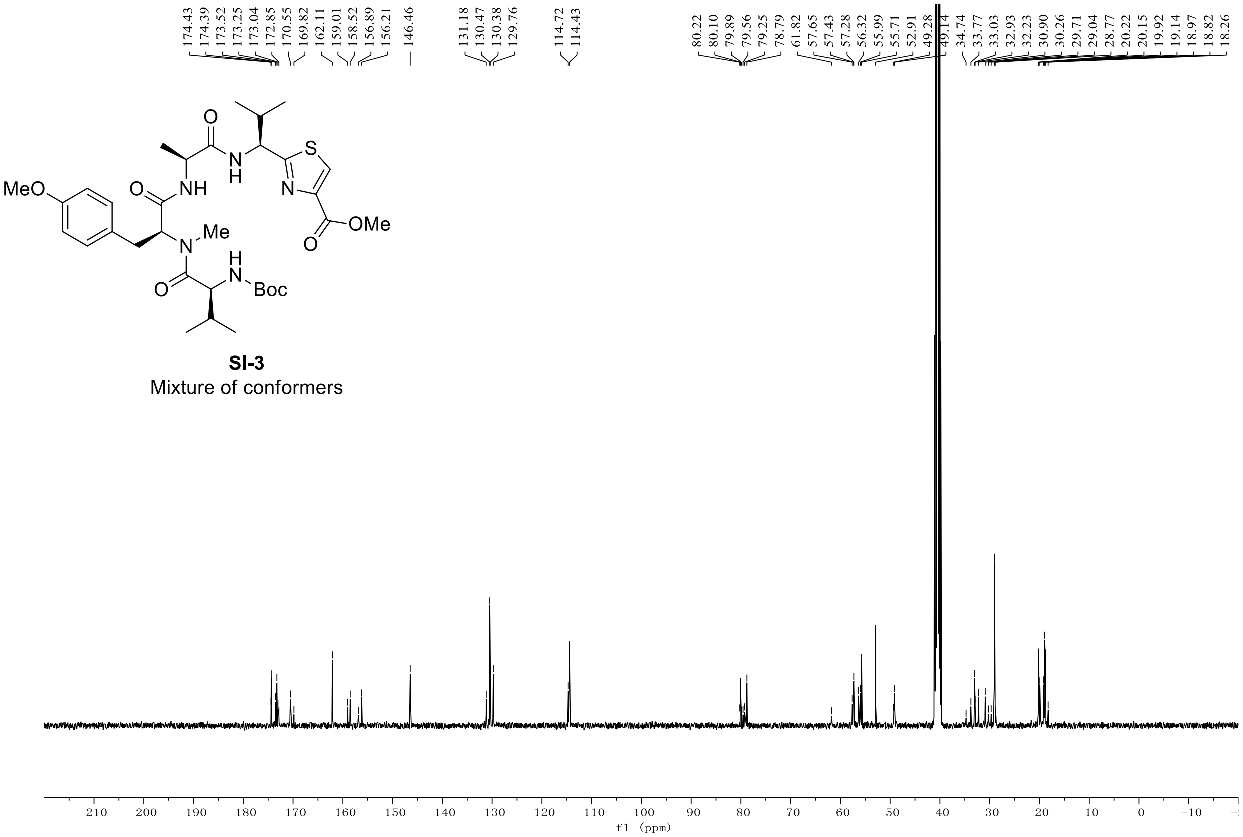

^{1}H NMR and ^{13}C NMR Spectra of Compound SI-4

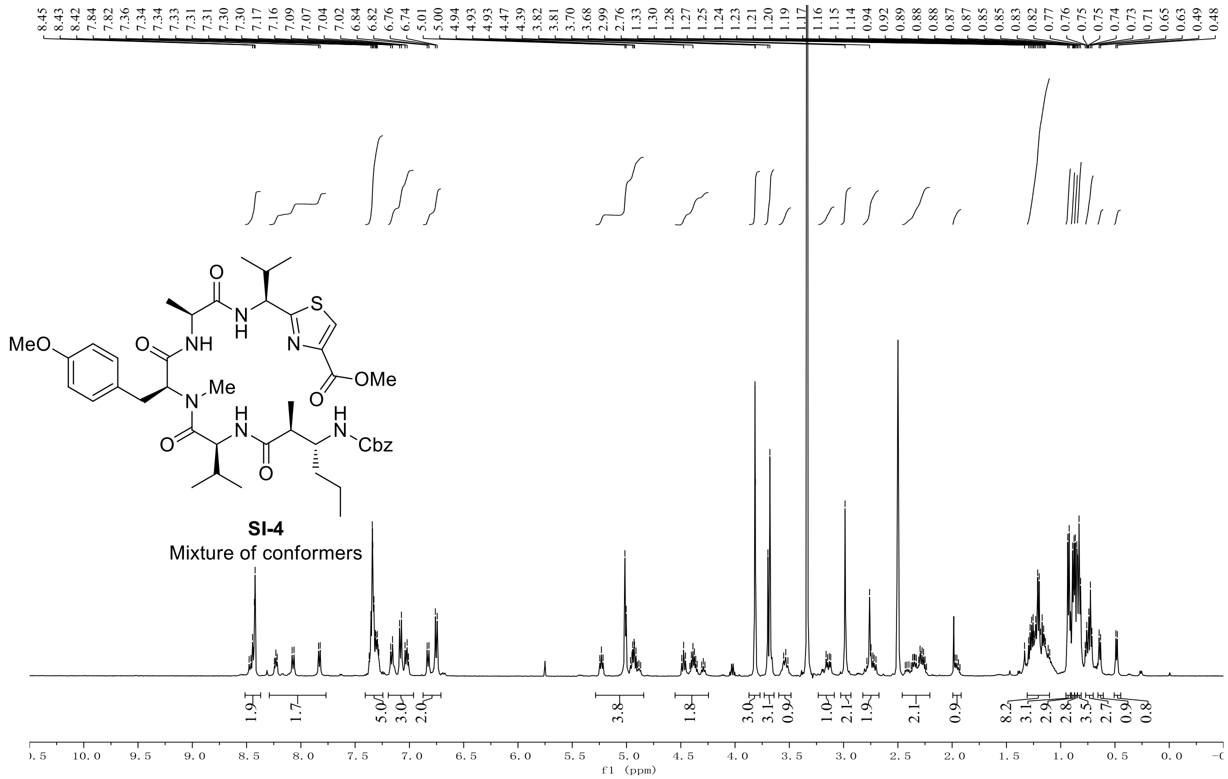

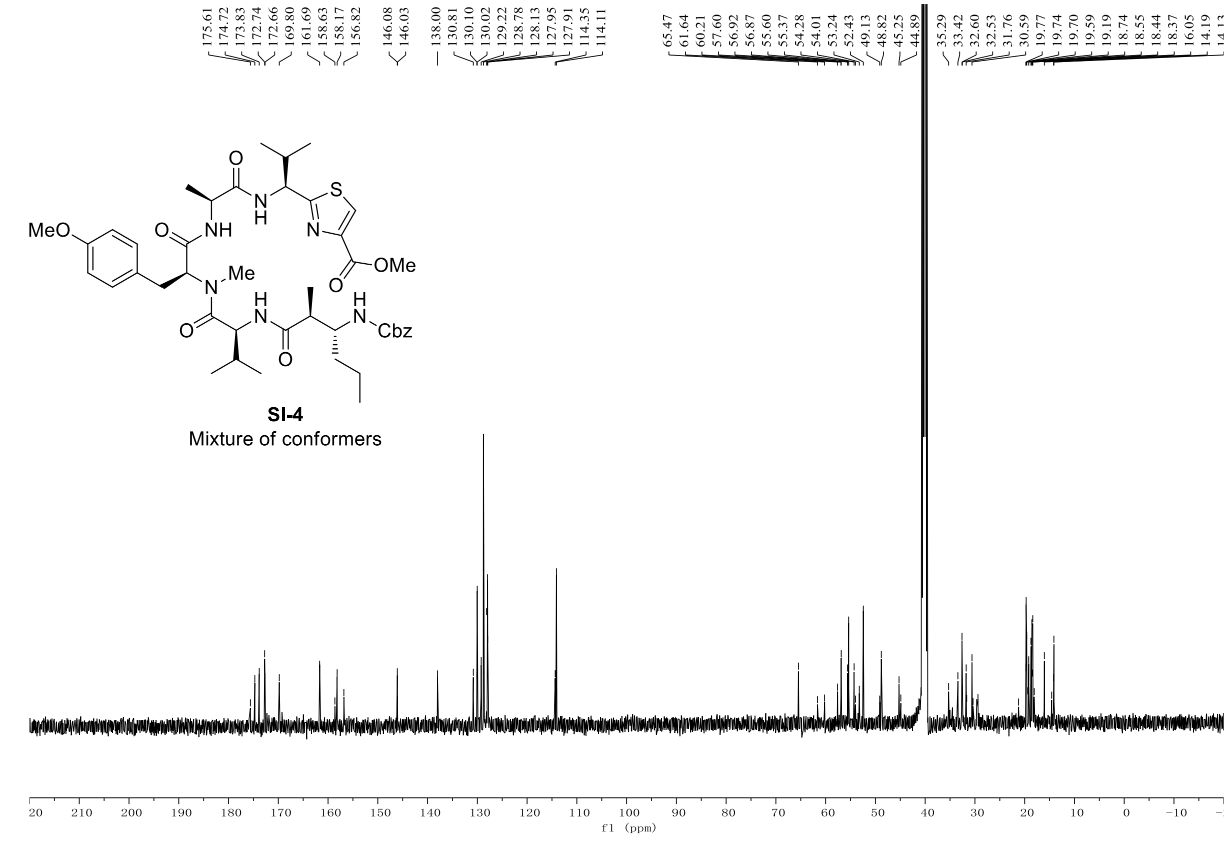

^{1}H NMR and ^{13}C NMR Spectra of Analogue 16

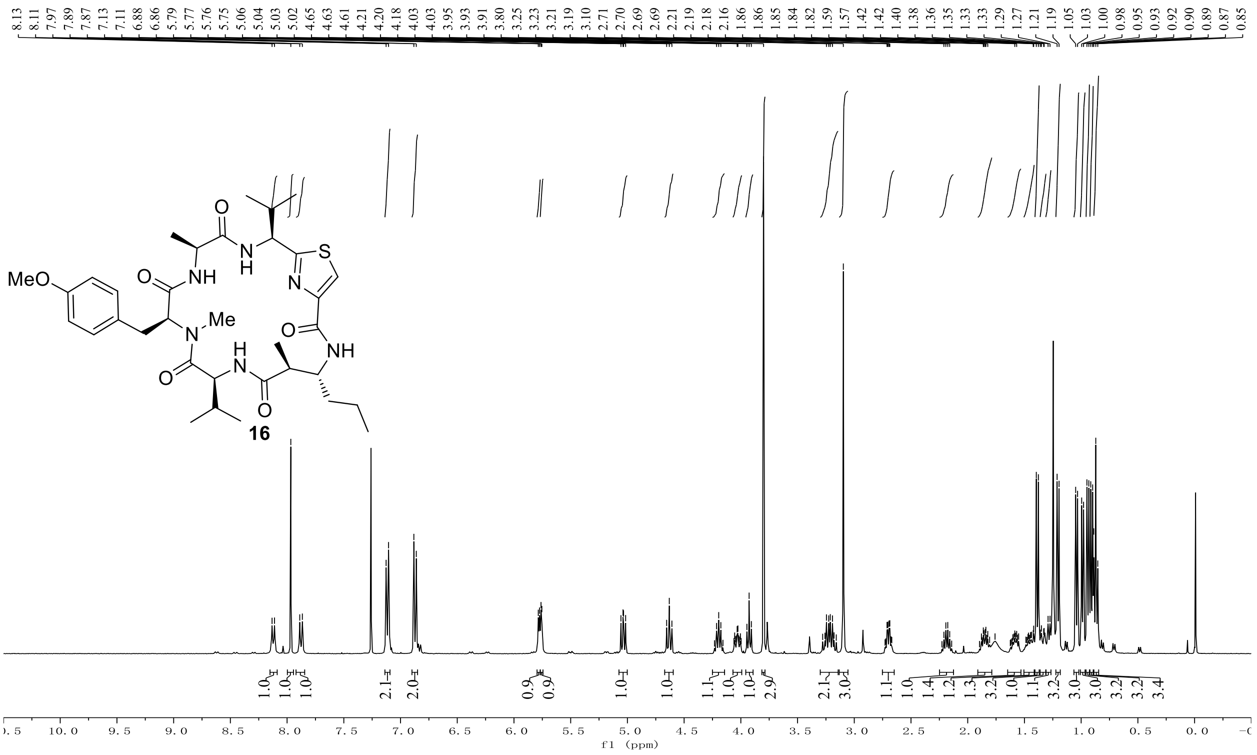

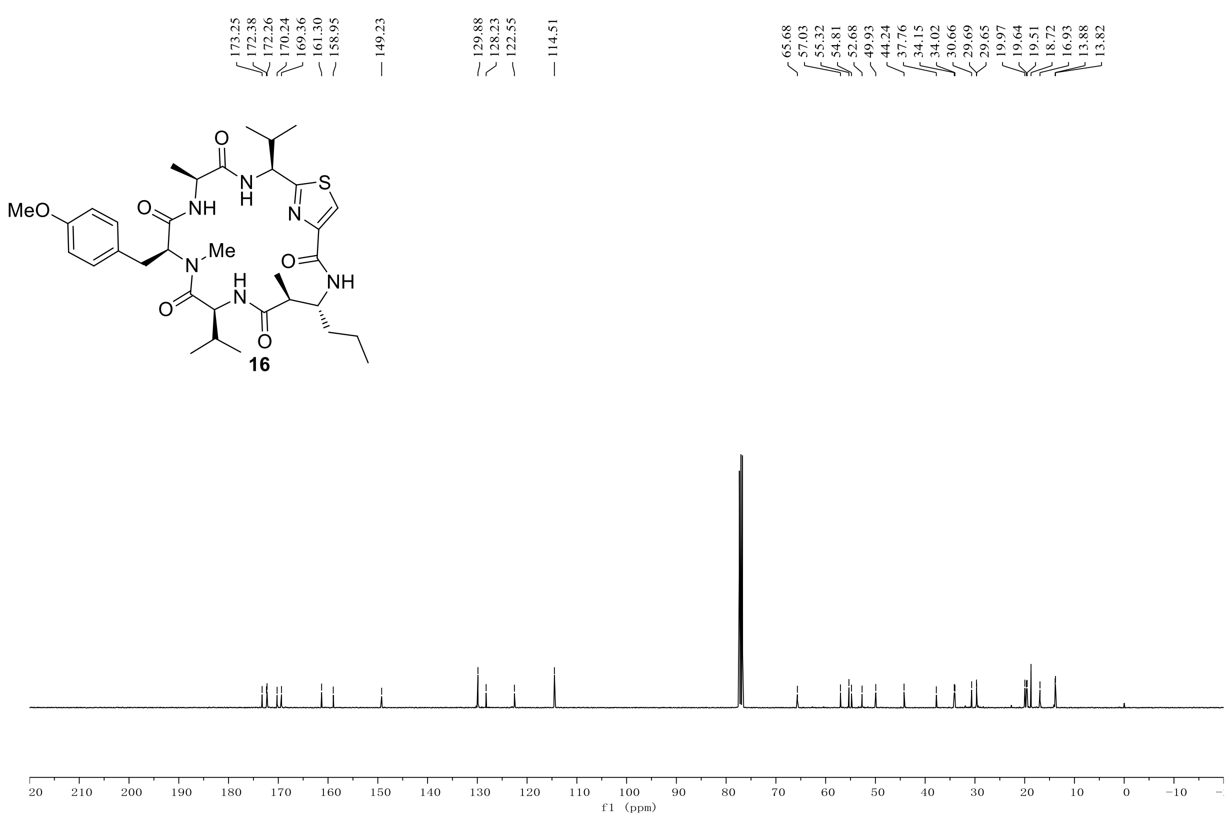

^{1}H NMR and ^{13}C NMR Spectra of Compound SI-5

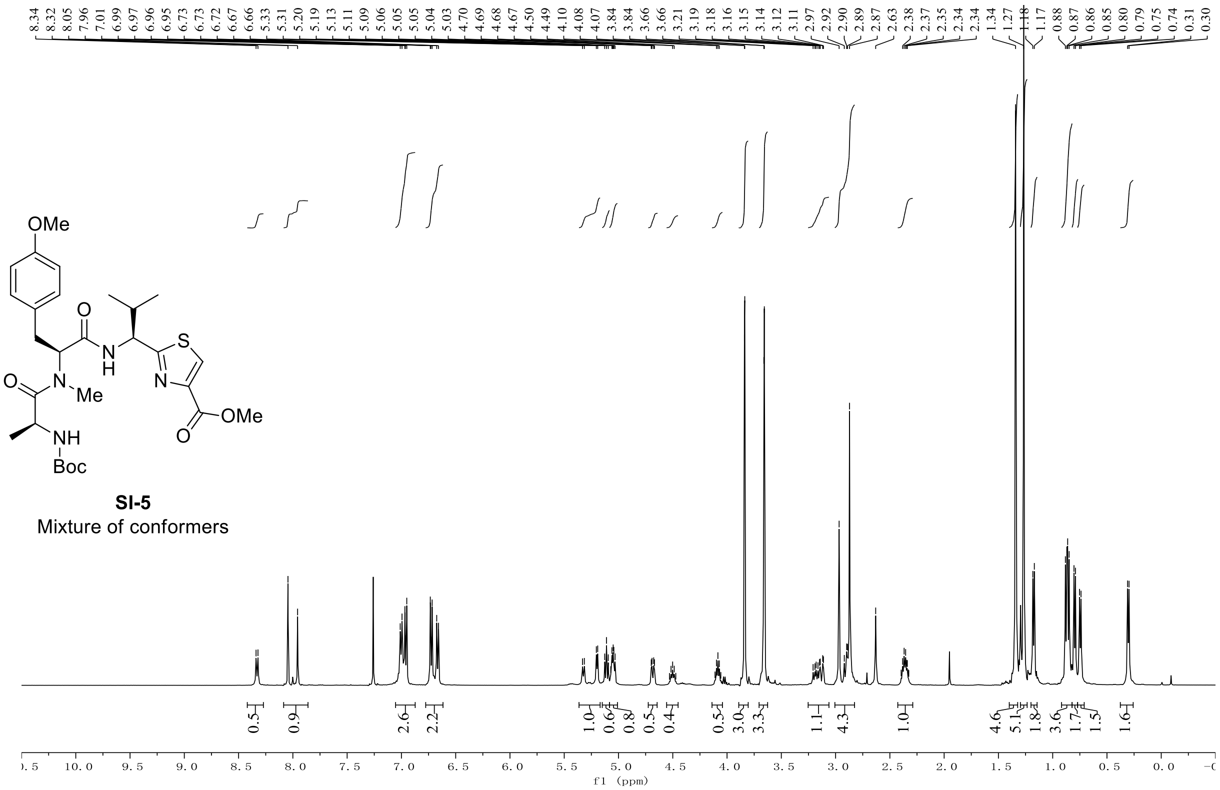

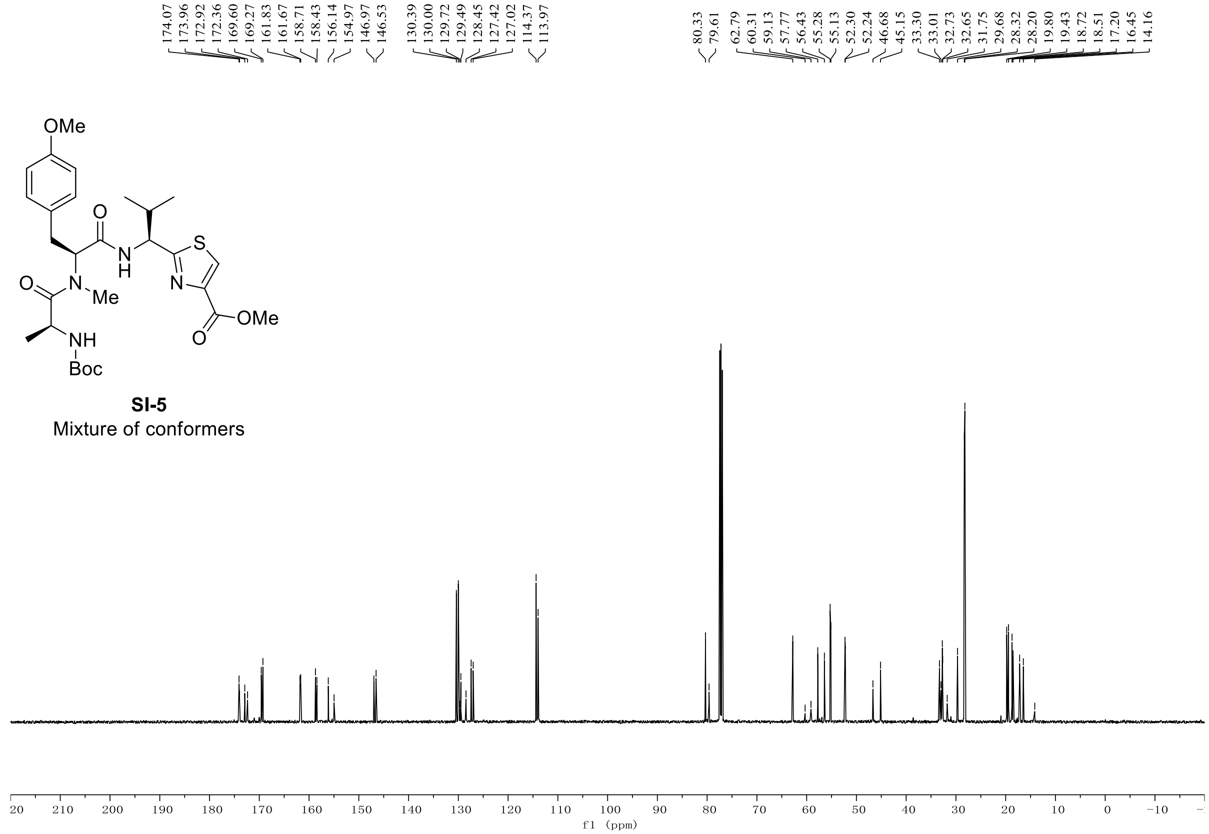

^{1}H NMR and ^{13}C NMR Spectra of Compound SI-6

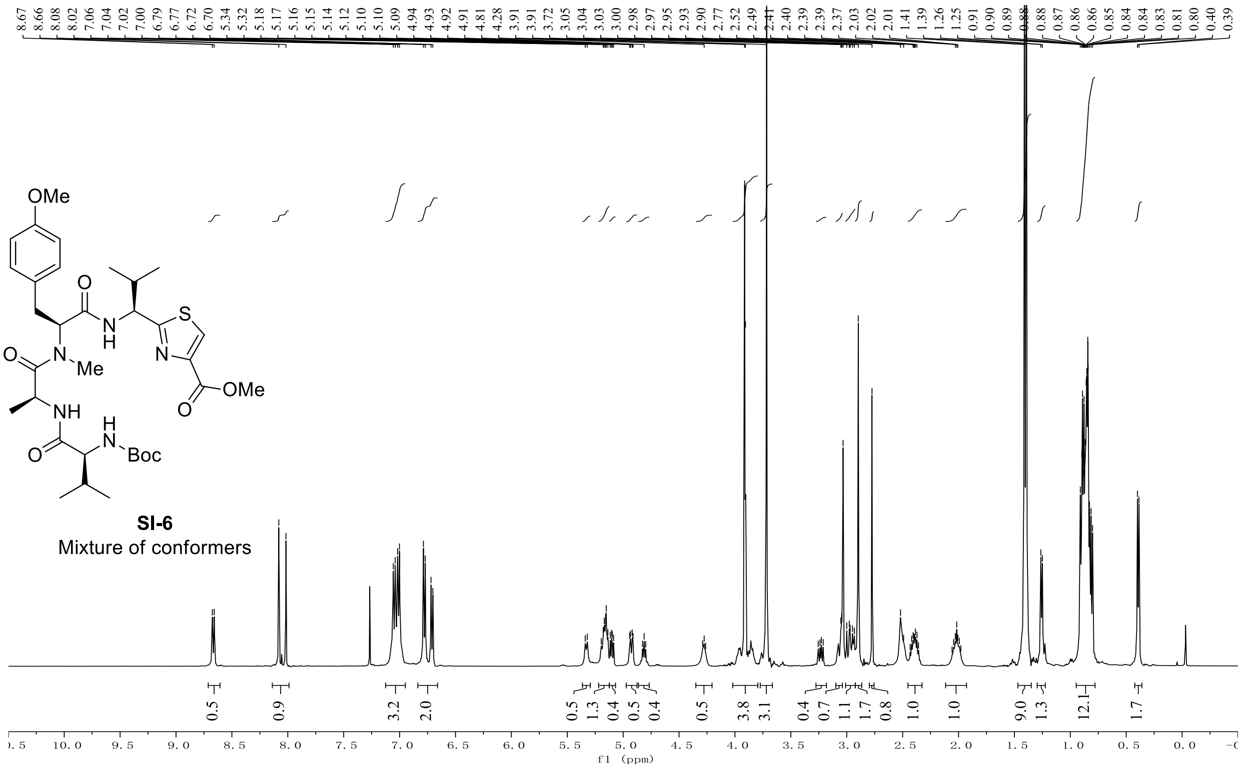

SI-6
Mixture of conformers

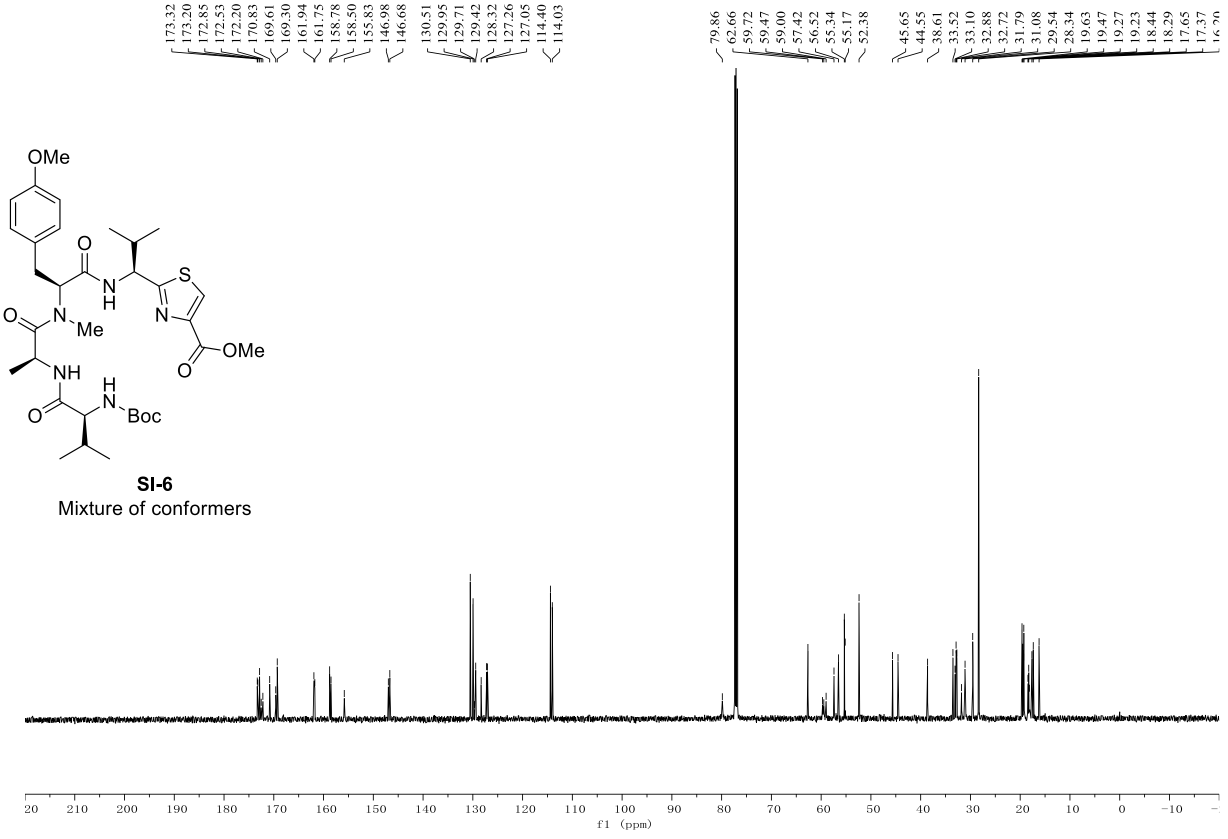

SI-6
Mixture of conformers

^{1}H NMR and ^{13}C NMR Spectra of Compound SI-7

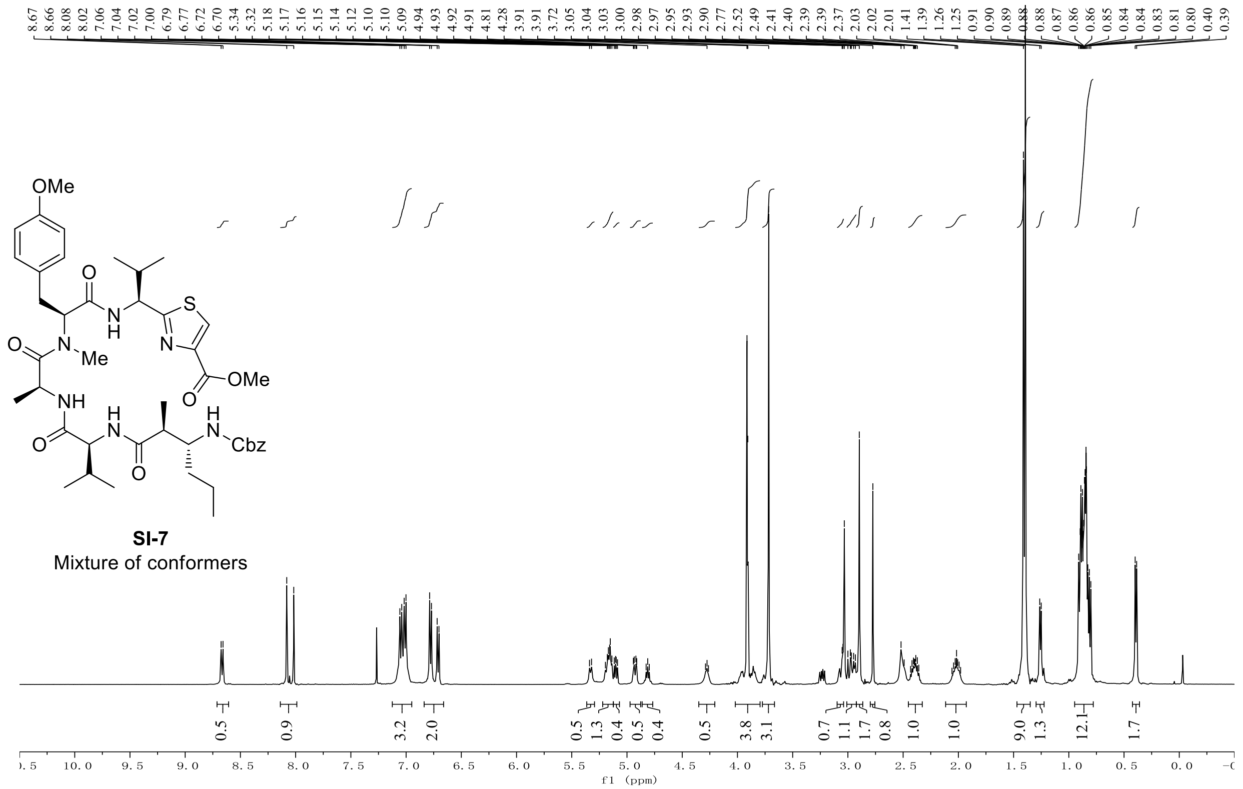

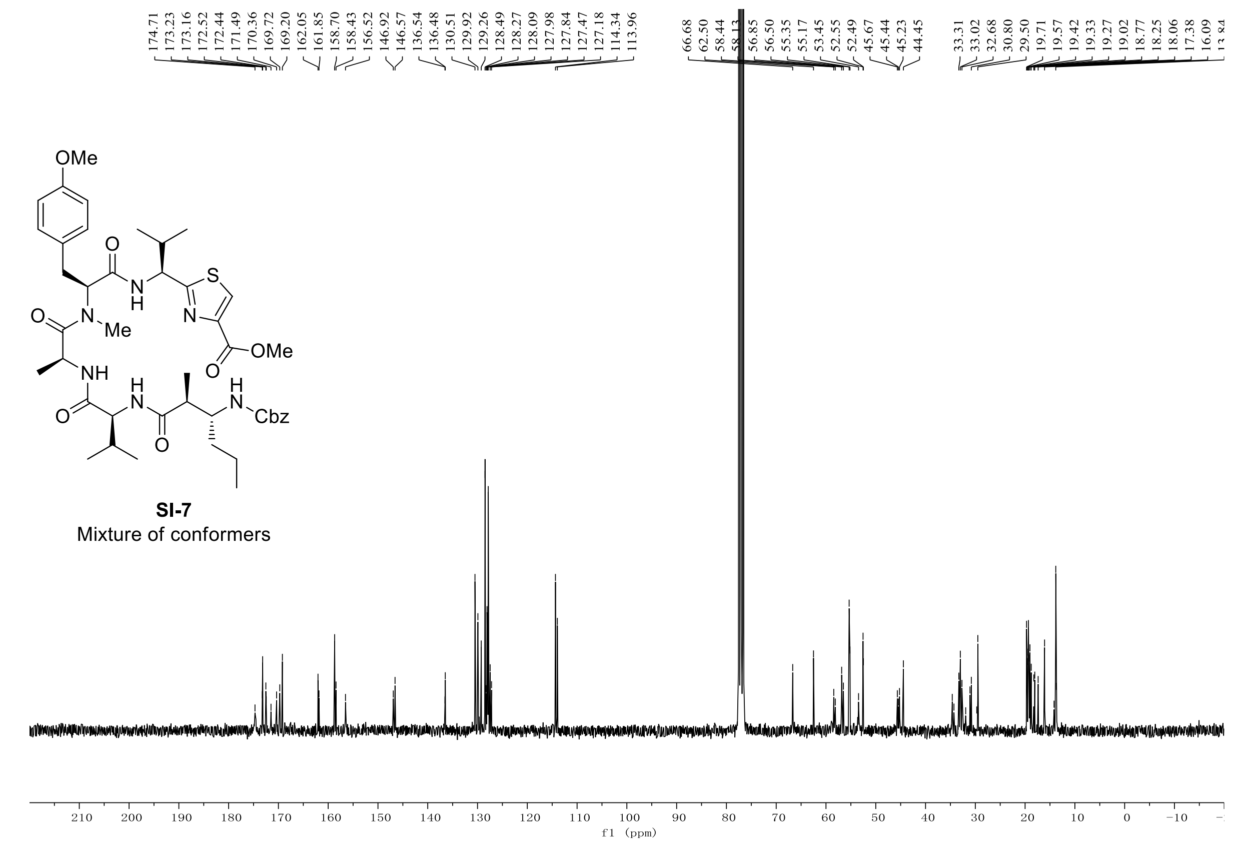

^{1}H NMR and ^{13}C NMR Spectra of Analogue 17

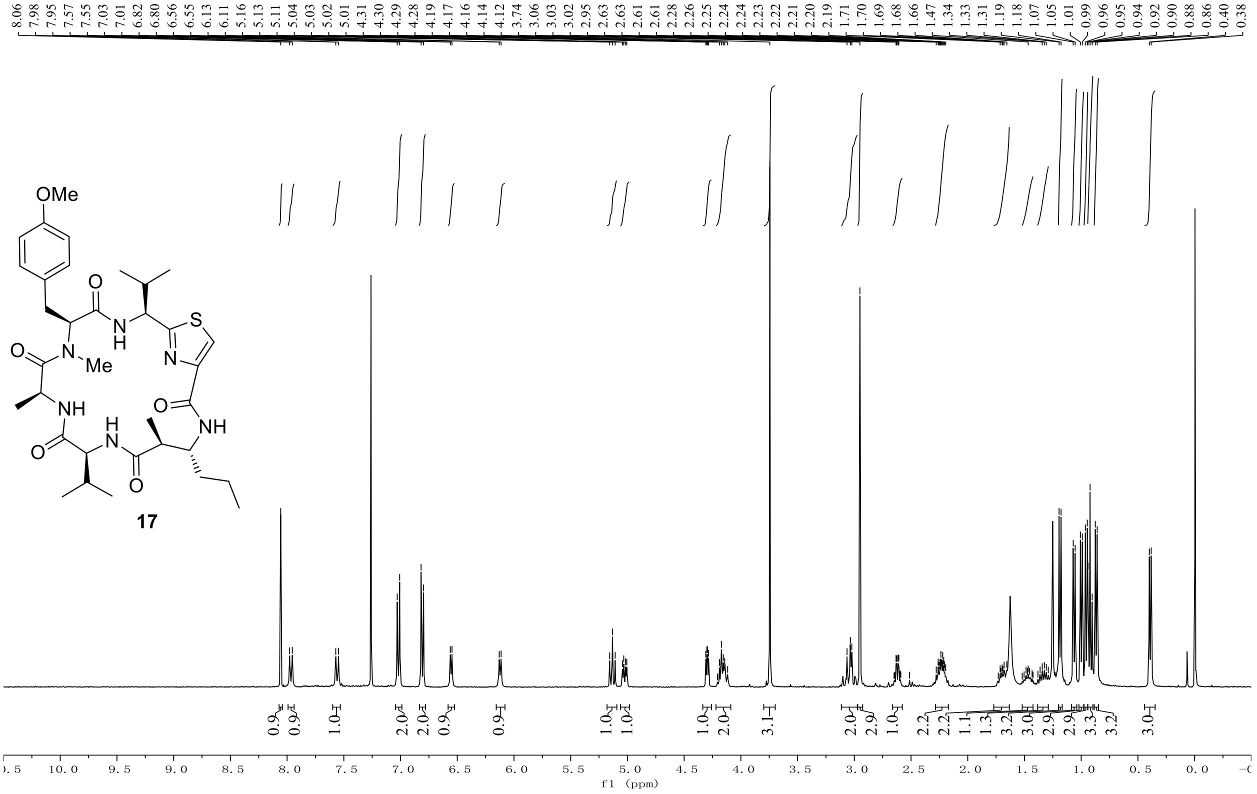

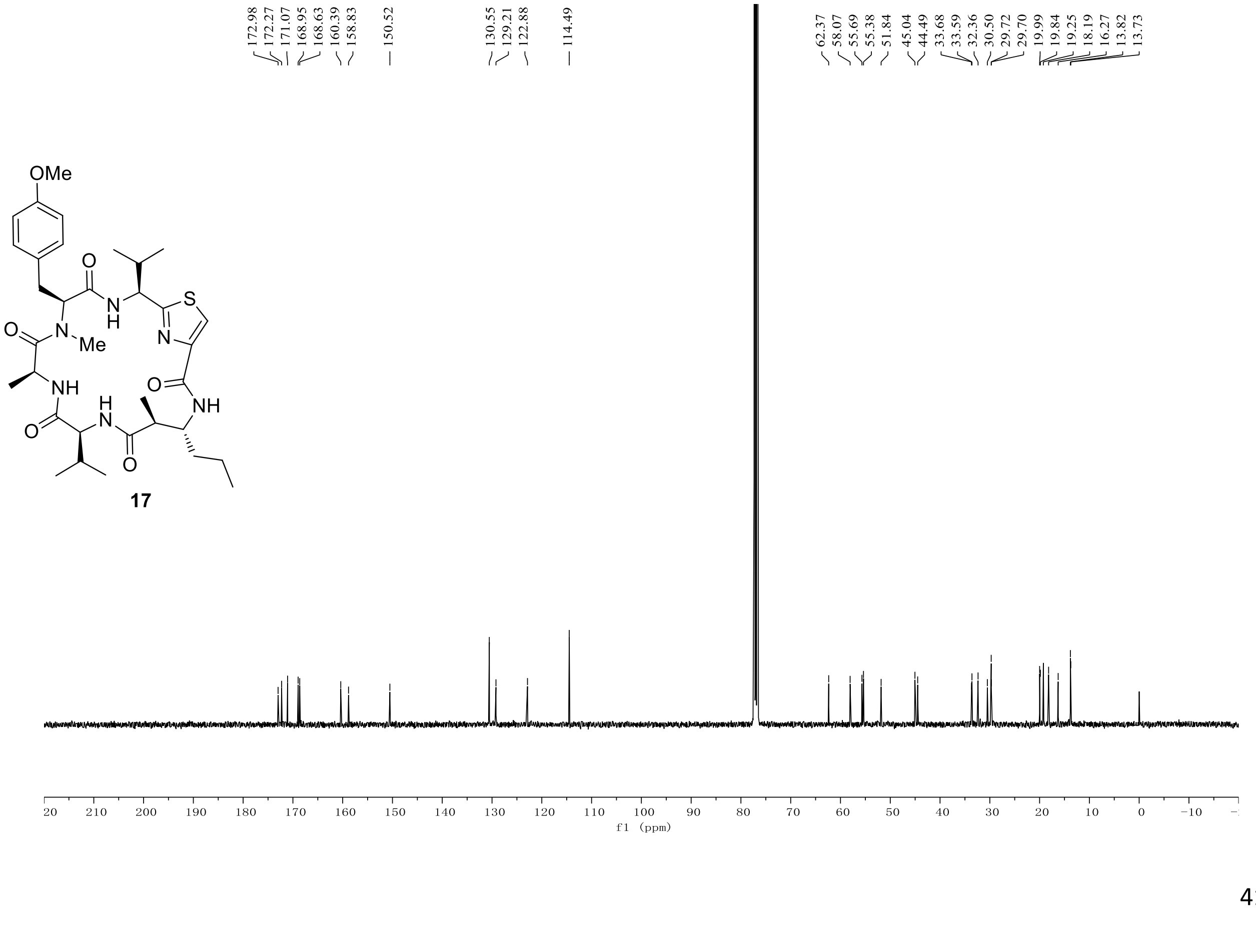

^{1}H NMR and ^{13}C NMR Spectra of Compound SI-8

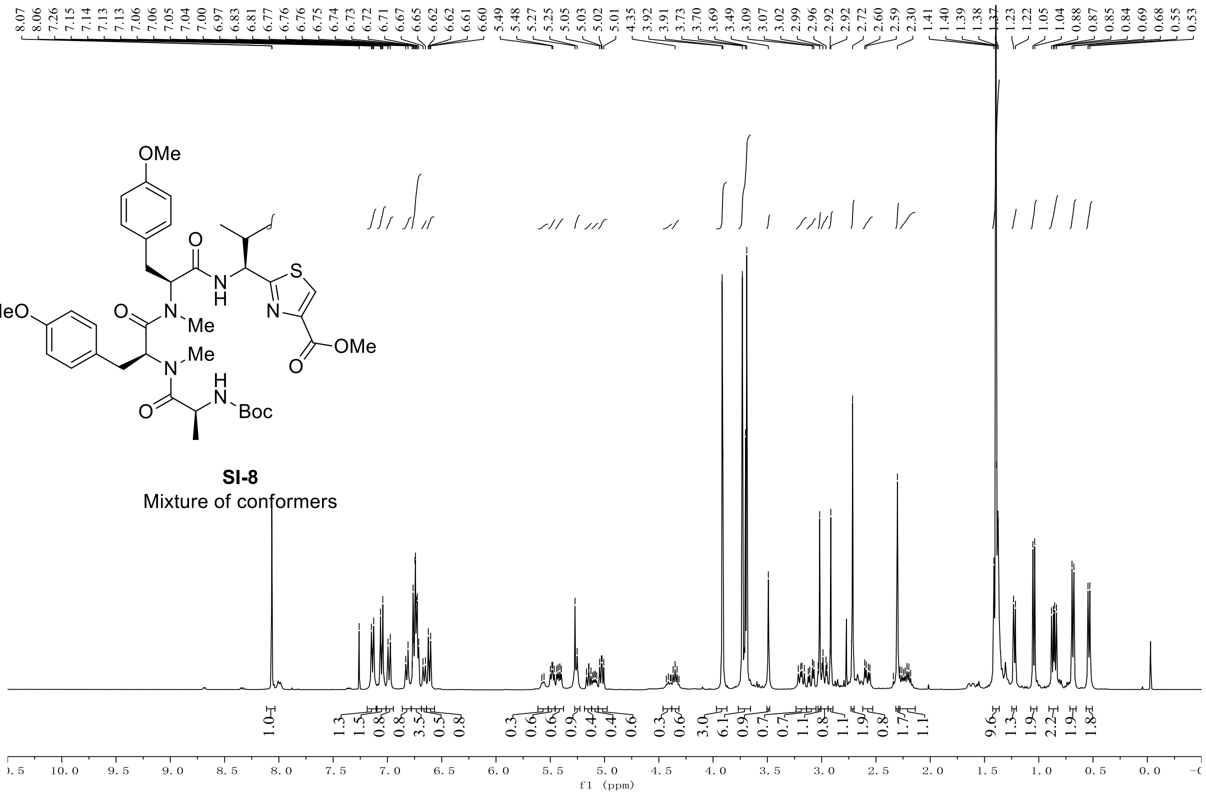

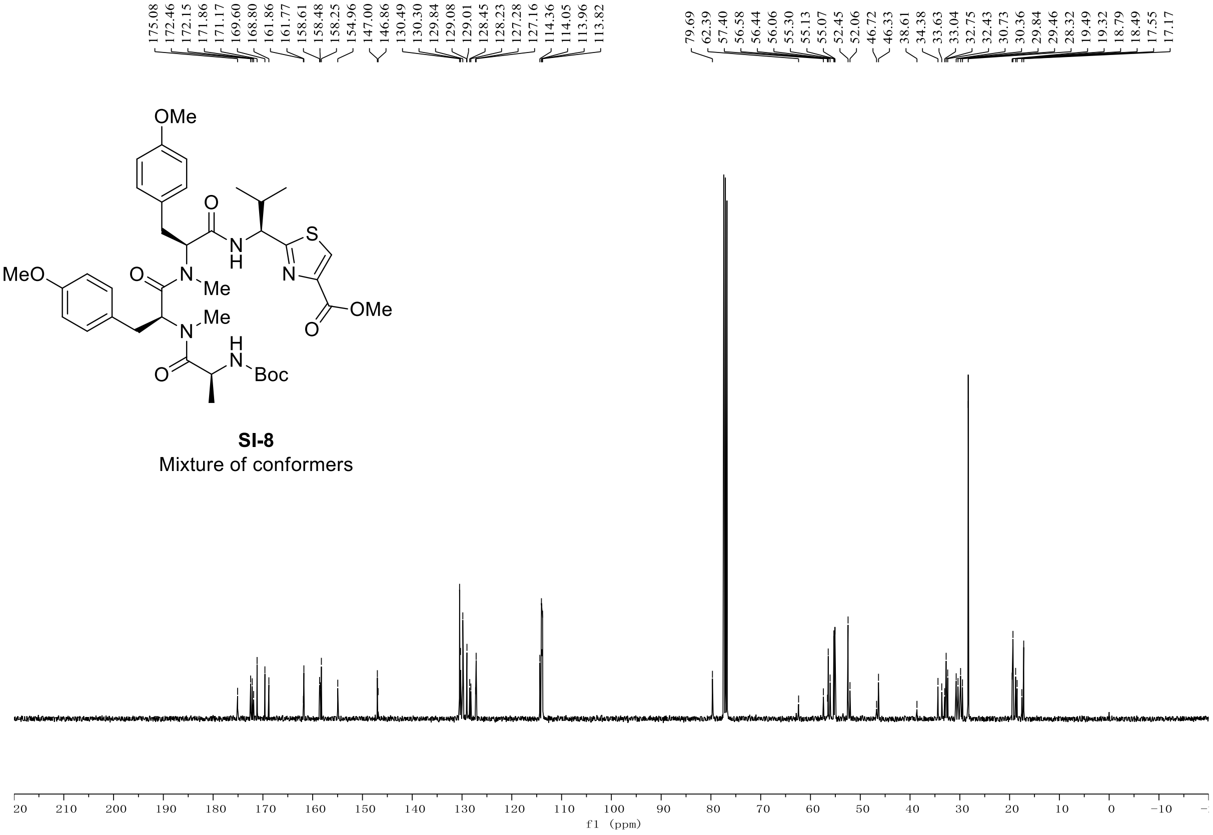

^{1}H NMR and ^{13}C NMR Spectra of Compound SI-9

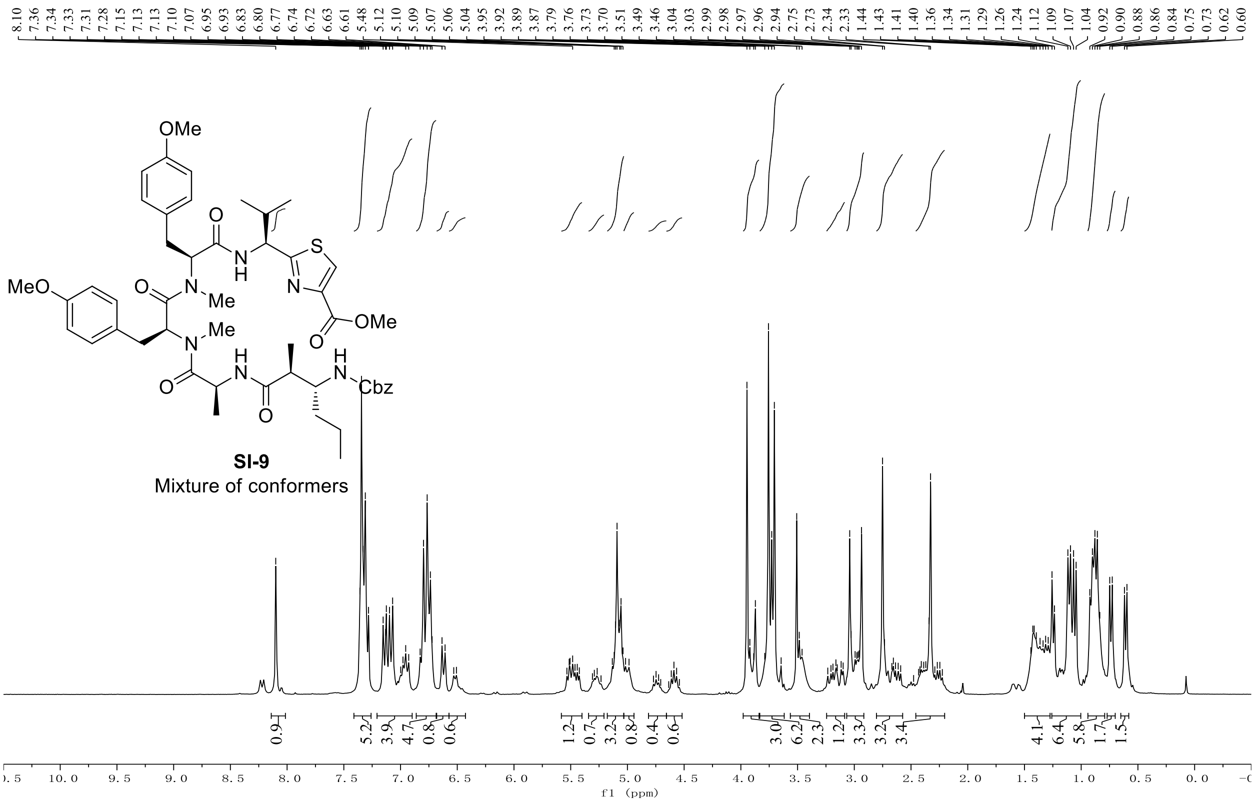

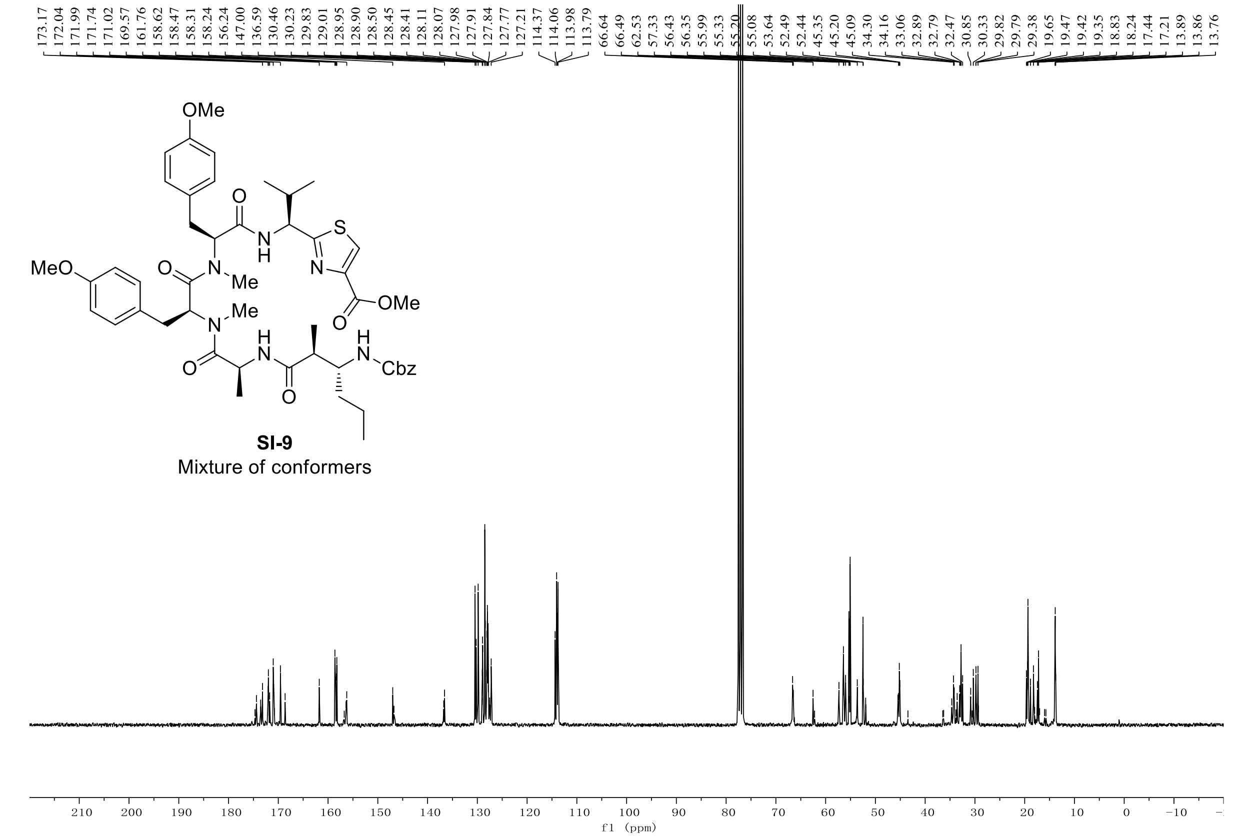

^{1}H NMR and ^{13}C NMR Spectra of Analogue 18

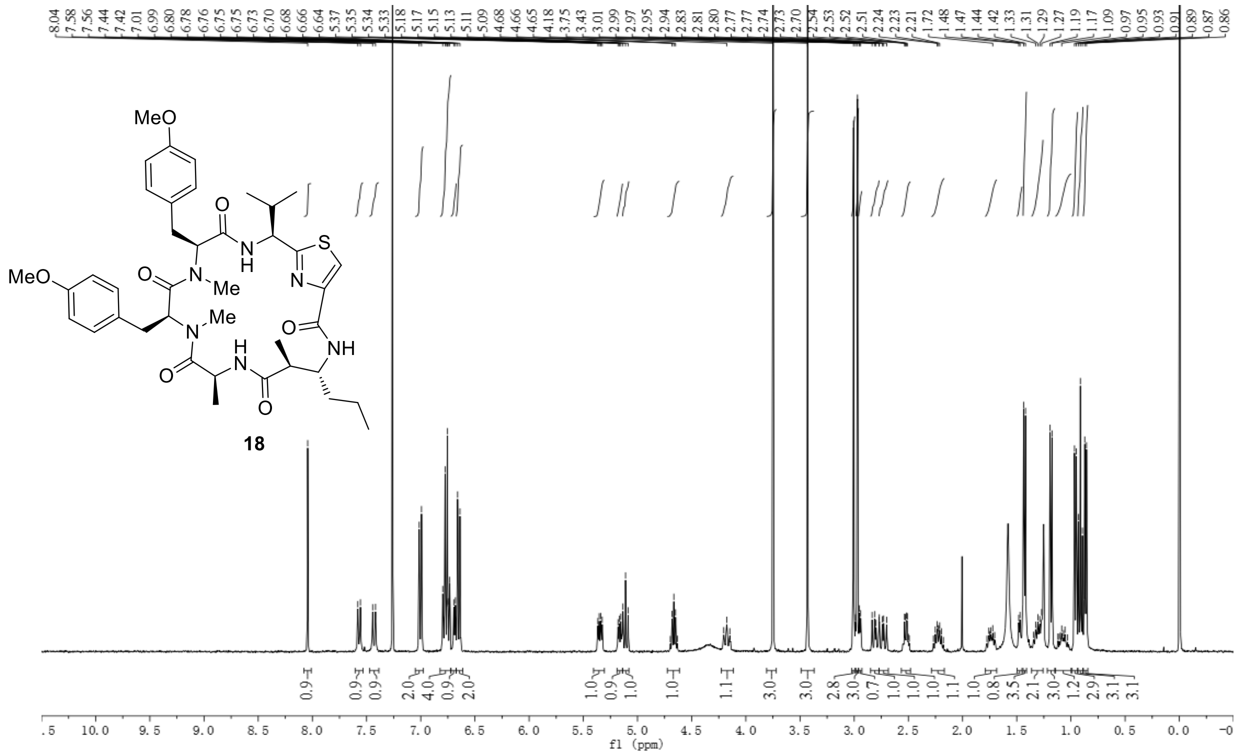

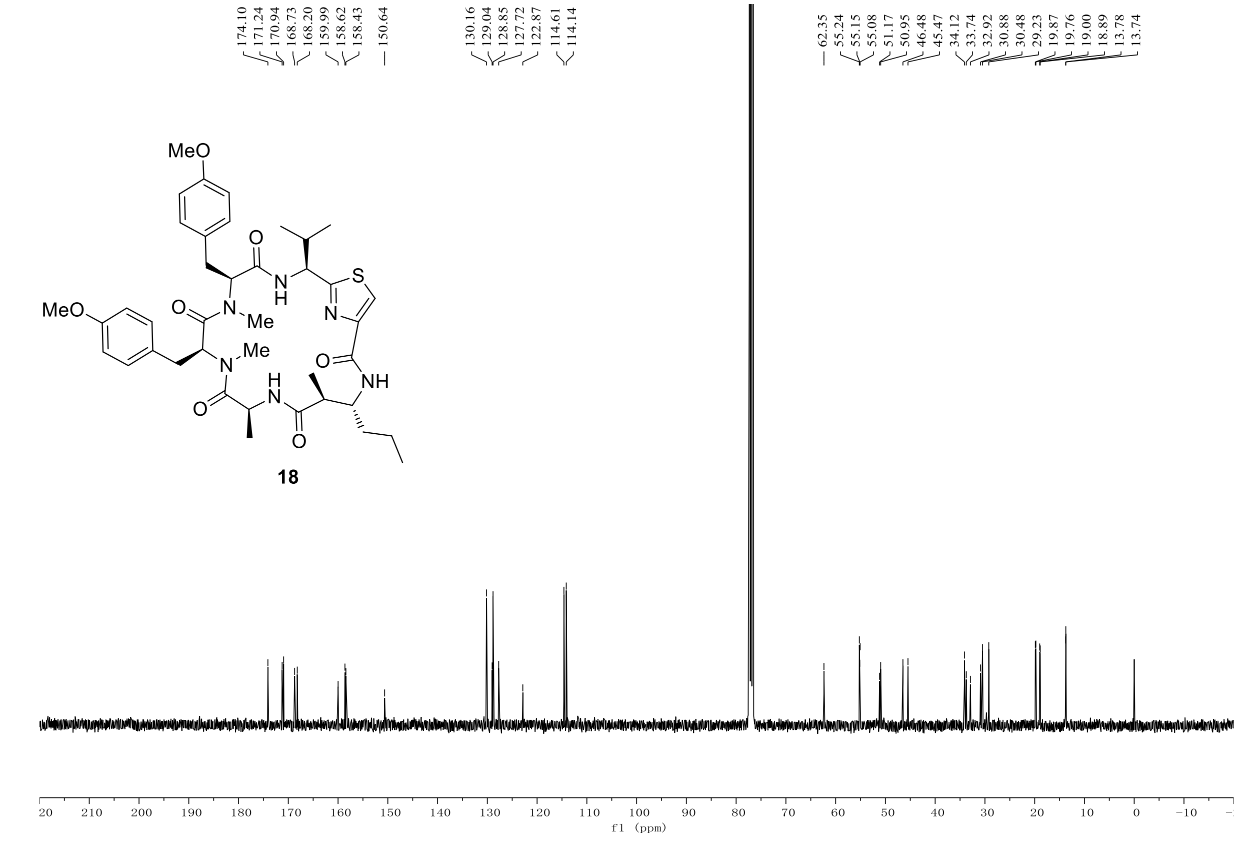

^{1}H NMR and ^{13}C NMR Spectra of Compound SI-10

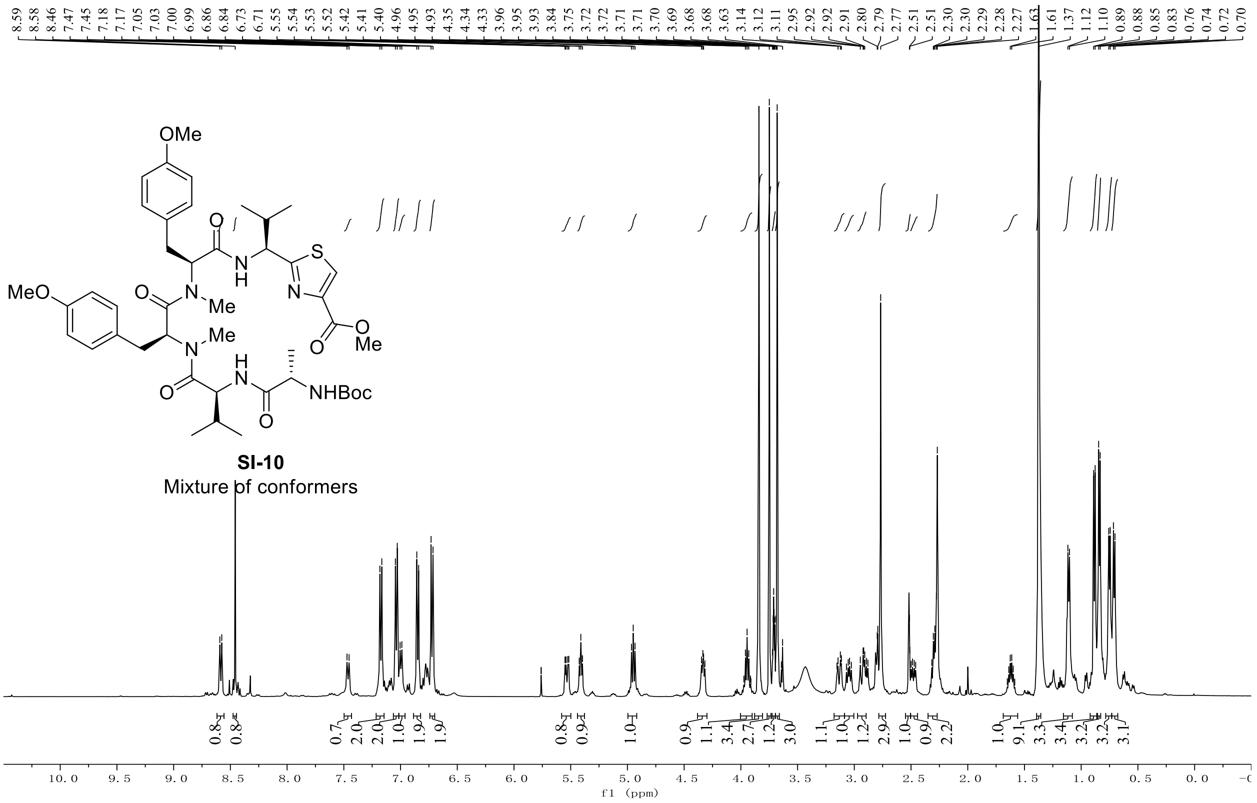

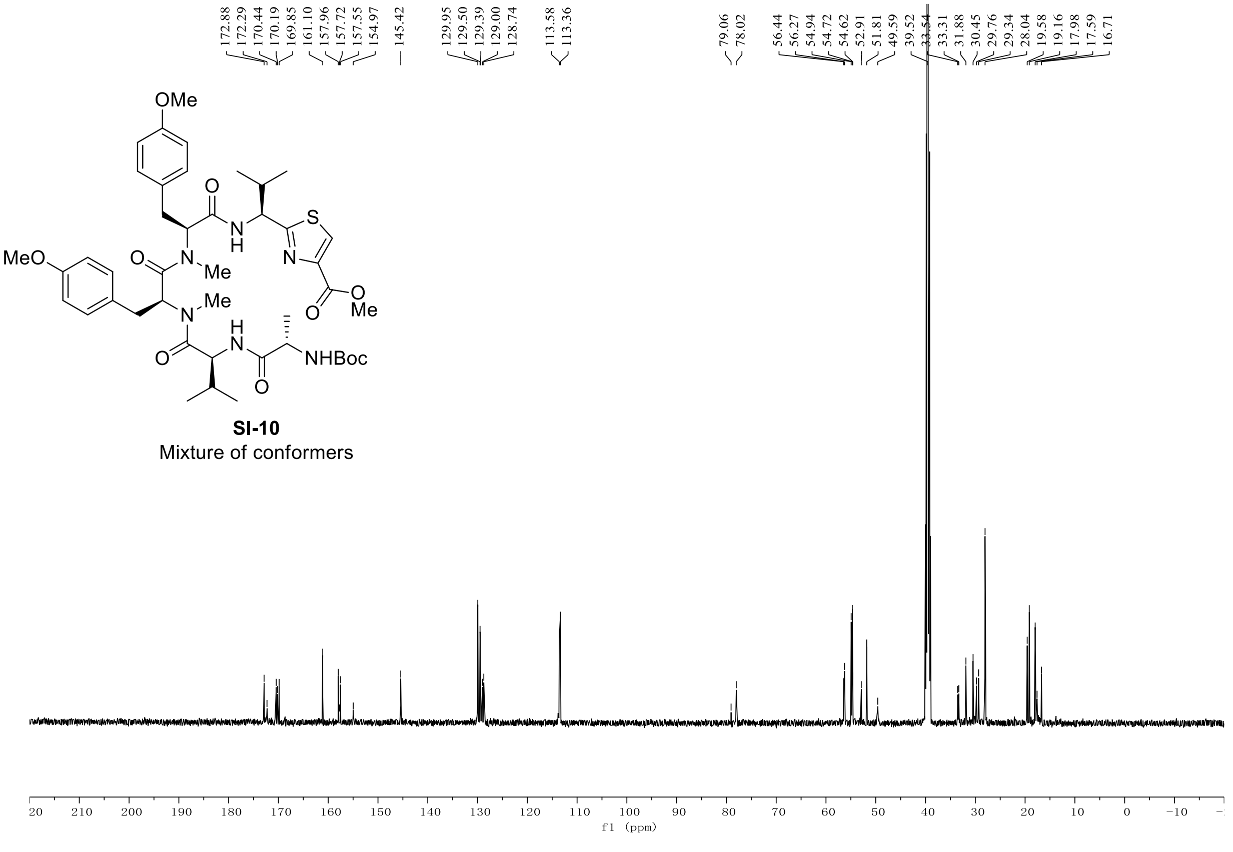

^{1}H NMR and ^{13}C NMR Spectra of Analogue 19

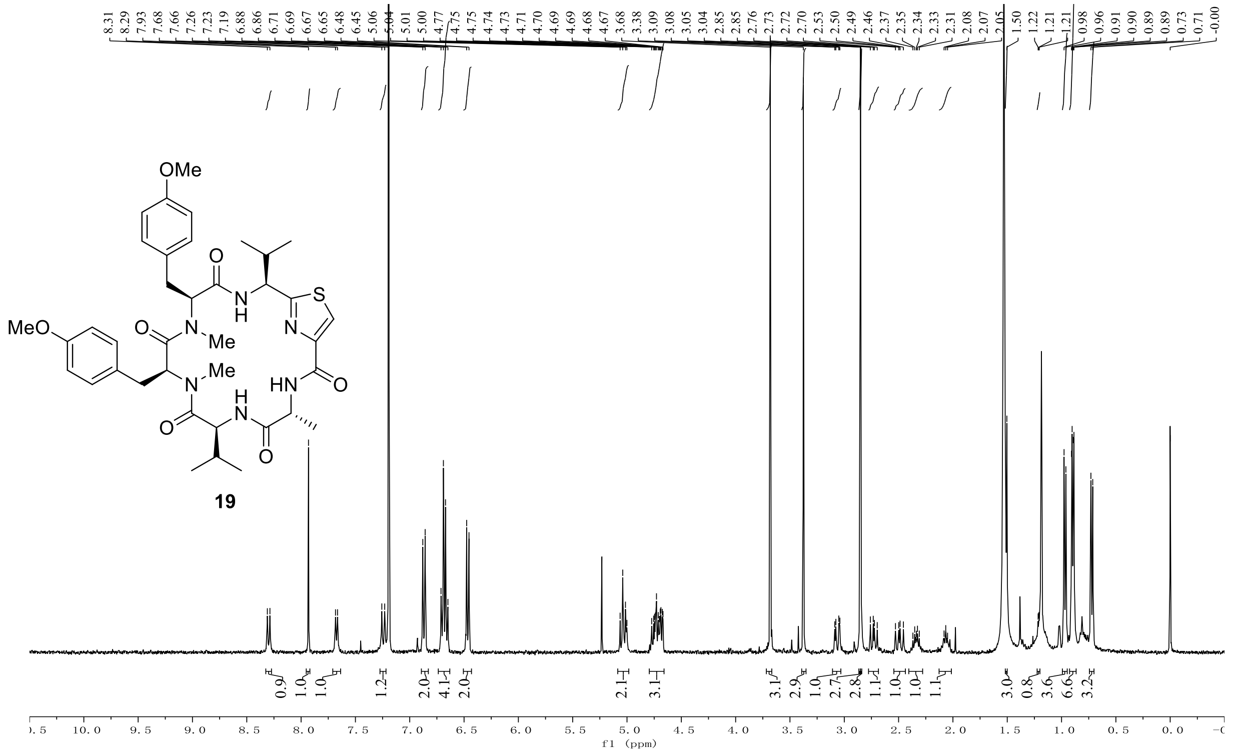

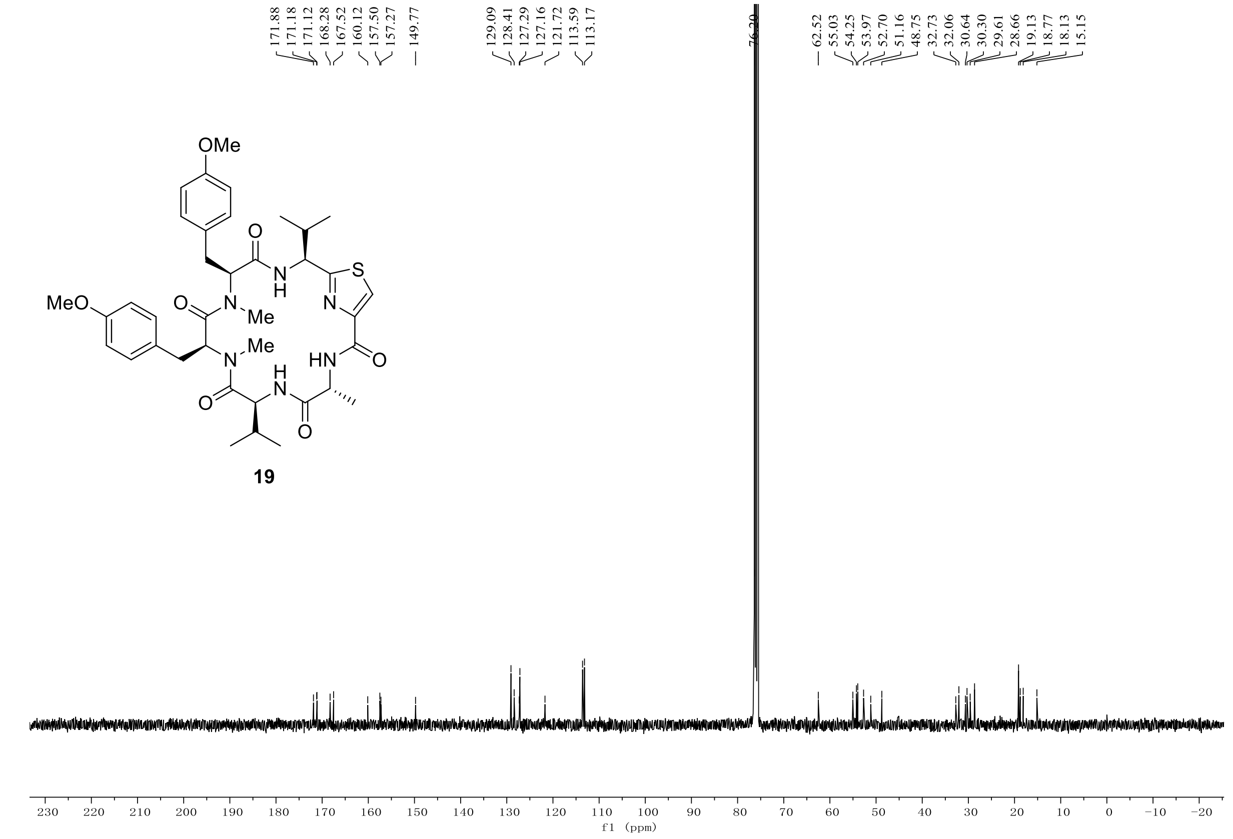

Supplement: Supplementary file 1 [file Data_Sheet_1.PDF]
